# Rh-Catalyzed Expansion of Azetidines to Pyrrolidines via Modulated Carbene Transfer

**DOI:** 10.1021/acs.orglett.6c03625

**Published:** 2026-10-17

**Authors:** Olivia M. Duke, Jensen J. Zerban, Xiang Lian, Jennifer M. Schomaker

**Affiliations:** Department of Chemistry, 5228 University of Wisconsin, 1101 University Avenue, Madison, Wisconsin 53706, United States

## Abstract

Reliable methods for accessing complex pyrrolidines without the need for extensive pre-functionalization or harsh reaction conditions are attractive. We report a Rh-catalyzed ring-expansion strategy, in which azetidines react with donor–acceptor carbene precursors to form azetidinium ylides that selectively rearrange to pyrrolidines. The protocol is operationally simple, proceeds under mild conditions, avoids slow addition of the carbene precursor, exhibits good functional group tolerance, and enables easy removal of the *N*-aryl group if desired. The resulting functional-group-rich *N*-aryl pyrrolidines are versatile intermediates for the efficient construction of complex, drug-like pyrrolidines. UV–Vis studies elucidate a key catalyst–substrate binding equilibrium dependent on the *N*-protecting group, providing mechanistic insight into the reaction’s unique behavior and informing future method development.

## Graphical abstract

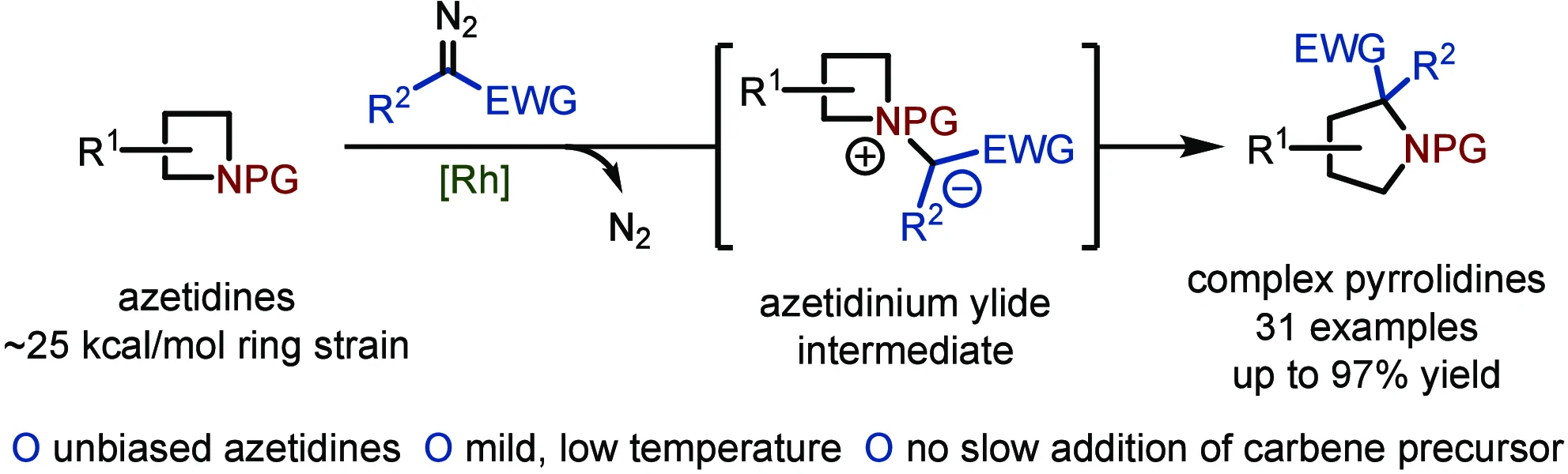

P yrrolidines are important motifs in drugs and many bioactive molecules (Figure 1A); thus, new methods for their synthesis are of interest.^1^ A recent review of FDA-approved pharmaceuticals showed pyrrolidines tied for the second-most prevalent motif,^2^ as their increased Fsp^3^ character compared to pyrroles can improve solubility and target binding.^3^ Common syntheses of pyrrolidines include 1,3-dipolar cycloadditions, cyclization of linear precursors, reduction of pyrrolidinones, and modifications to naturally occurring pyrrolidines.^4^ Skeletal editing^5^ in the form of a one-atom ring contraction of pyridines to pyrrolidines under visible light has also been used, but the products are limited to 2-azabicyclo[3.1.0]­hex-3-ene derivatives.^6^ In contrast, one-carbon additions into azetidines^7^ allow the incorporation of additional functionality during ring expansion.

**Figure 1:**
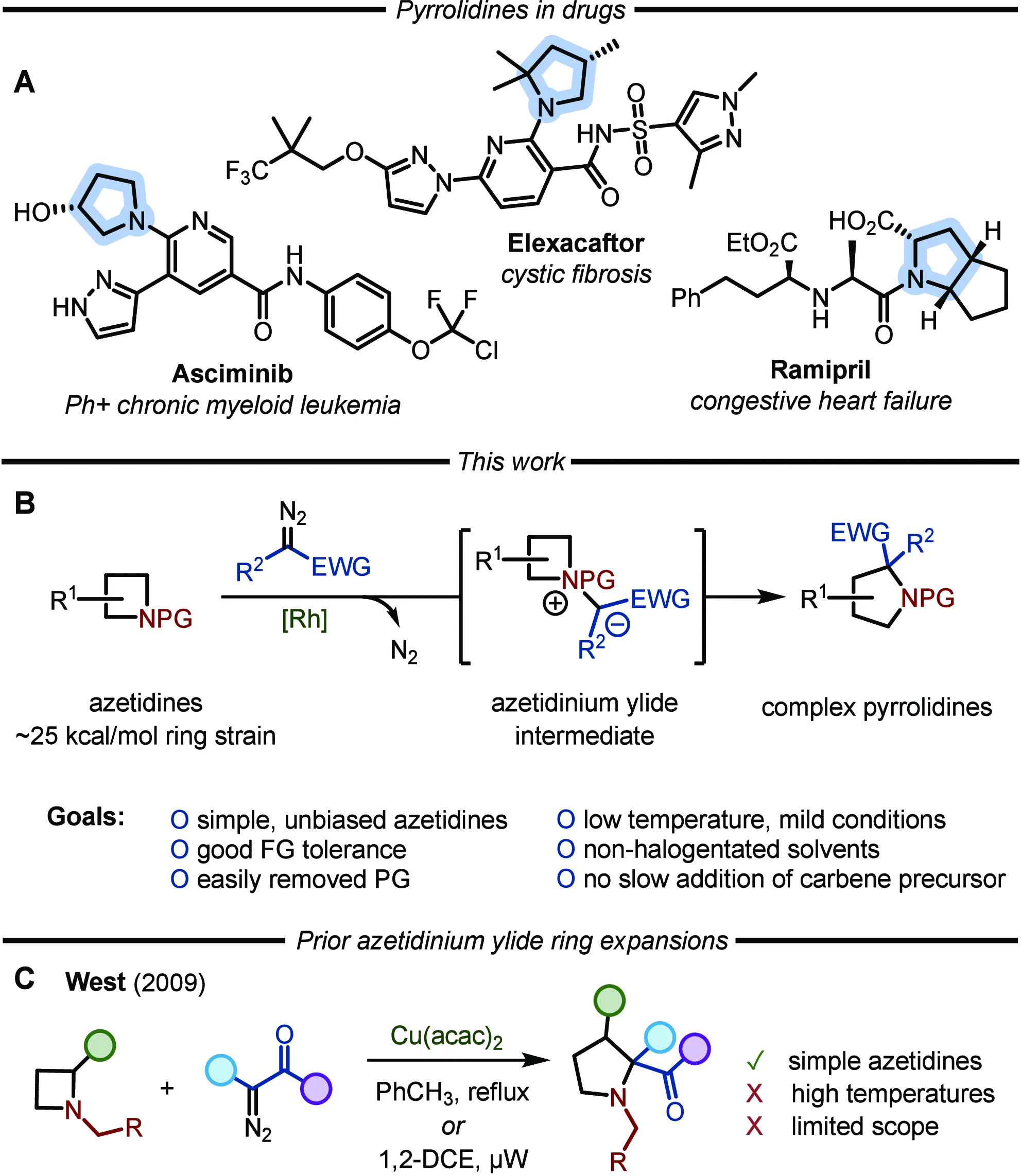
Background and goals for this work.

We have previously developed intermolecular expansions of aziridines to azetidines and piperidines via aziridinium ylide intermediates.^8,9^ Herein, we apply mechanistic insights gained from these studies to a simple one-carbon expansion of azetidines to highly functionalized pyrrolidines with good functional group tolerance. The reaction proceeds under mild conditions, employs an easily removable N-protecting group, and avoids the slow addition of the carbene precursor (Figure 1B).

The West group showed that the addition of *N*-alkyl azetidines to metal-supported acceptor/acceptor carbenoids^10,11^ could be catalyzed by Cu­(acac)_2_ ^10g,11^ in refluxing PhMe with a 12 h slow addition or microwaving to 180 °C in 1,2-DCE (Figure 1C). Koh reported Cu-catalyzed difluorocarbene insertion into *N*-alkyl azetidines to give γ-lactams, although azetidinium ylides were not involved.^12^ Rh-catalyzed [4 + 1] cycloadditions of methyleneazetidines furnished 4-methylene pyrrolidines,^13,14^ but an exocyclic alkene was needed to increase ring strain and stabilize key intermediates. Herein, we report an operationally convenient transformation of simple azetidines that requires no slow addition of the carbene precursor, displays good functional group tolerance, proceeds at room temperature, and transfers stereochemical information from chiral azetidines to products, which can be diversified into drug-like scaffolds.

The identity of the nitrogen substituent has a profound effect on the reaction outcome. Our previous work on aziridine expansions required sufficient nucleophilicity to attack the Rh-supported carbene but adequate fluxionality to prevent irreversible binding of the heterocycle to Rh.^8^ We evaluated *N*-substituted azetidines for similar modulated nucleophilicity and found a stark division in reactivity. *N*-Benzylazetidine binds irreversibly to Rh, preventing the formation of metal-bound carbenoid species (Figure 2A, entry 1),^10,11^ while *N*-carbonyl or *N*-sulfonyl azetidines fully consumed the diazoester but gave no **3aa** (Figure 2B, entries 2–4). However, *N*-phenylazetidine **1a** (Figure 2B, entry 5) resulted in a promising 37% yield of **3aa**. Further optimization using **1a** showed a surprising increase in the yield to 57% using Et_2_O as the solvent (Figure 2C, entry 2). The addition of the diazoester in a single aliquot at the beginning of the reaction gave results (61%, entry 3) comparable to those of the slow addition conditions typically required to avoid rapid carbene dimerization. Finally, a more robust^15^ Rh_2_(esp)_2_ catalyst increased the yield of **3aa** to 92% (entry 4); yields remained comparable (89%, entry 5) even when the catalyst loading was lowered to 2 mol % (1.5 mg at 0.1 mmol scale).

**Figure 2:**
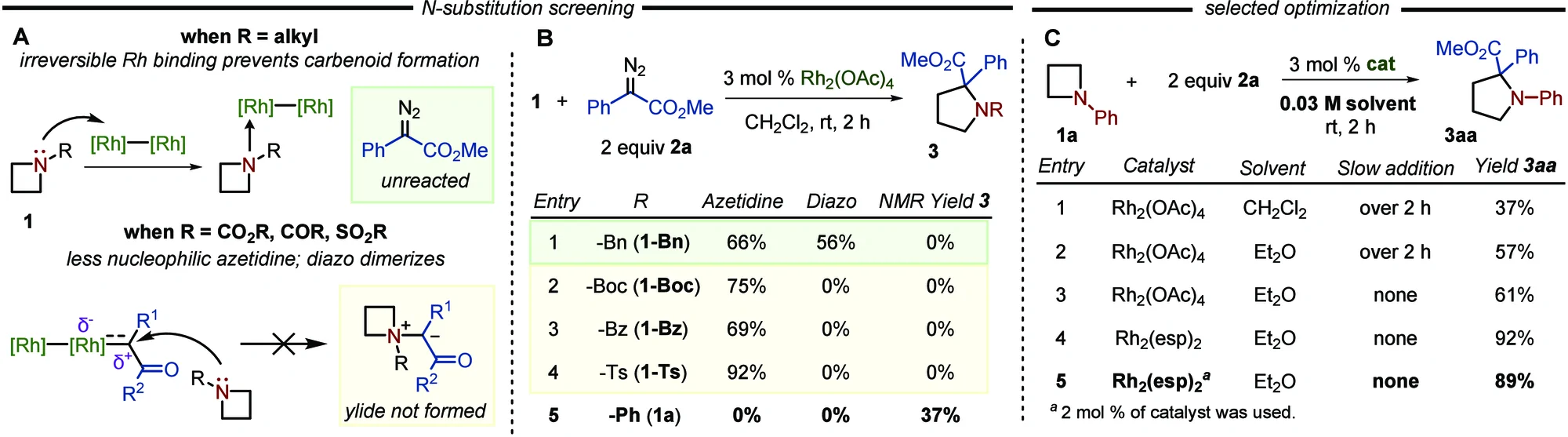
Optimization of azetidine *N*-substitution and reaction conditions.

Results from optimization studies, namely, the crucial *N*-Ph substitution, use of Et_2_O, and no need for slow addition, prompted further investigation into the reaction mechanism. Lewis basic additives,^16^ including structurally similar *N*,*N*-diethylanilines, are known to modulate reactivity in Rh-catalyzed carbene insertion reactions; we reasoned that the *N*-Ph substitution attenuates binding of the azetidine to Rh and prevents rapid carbenoid formation/dimerization.

Binding of **1a** to Rh_2_(esp)_2_ was next probed by UV–Vis. Dirhodium complexes supported by bridging carboxylates are known to form 1:1 and 2:1 adducts with Lewis bases, where free acid **A** (Figure 3A), 1:1 adduct **B**, and 2:1 adduct **C** can coexist in solution.^17–24^ Examination of the UV–vis spectrum for different concentrations of **1a** indicated that all three species coexist in solution (Figure S1-12).^17–24^ As the equilibria between Rh_2_(esp)_2_:**1a** adducts are likely key to the observed reactivity, we examined the binding of several azetidines to Rh_2_(esp)_2_ (Figure 3A). At higher [**1**], the 2:1 adduct is observed for *N*-Ph and *N*-Bn azetidines, while no adducts form with *N*-Boc or *N*-Ts azetidines. Surprisingly, a 1:1 adduct forms with *N*-Bz azetidine at high [**1**], although only dimerization of the diazoester was noted experimentally (Figure 2B, entry 3).^25^ Et_2_O was also observed to form a 1:1 adduct with Rh_2_(esp)_2_.

**Figure 3:**
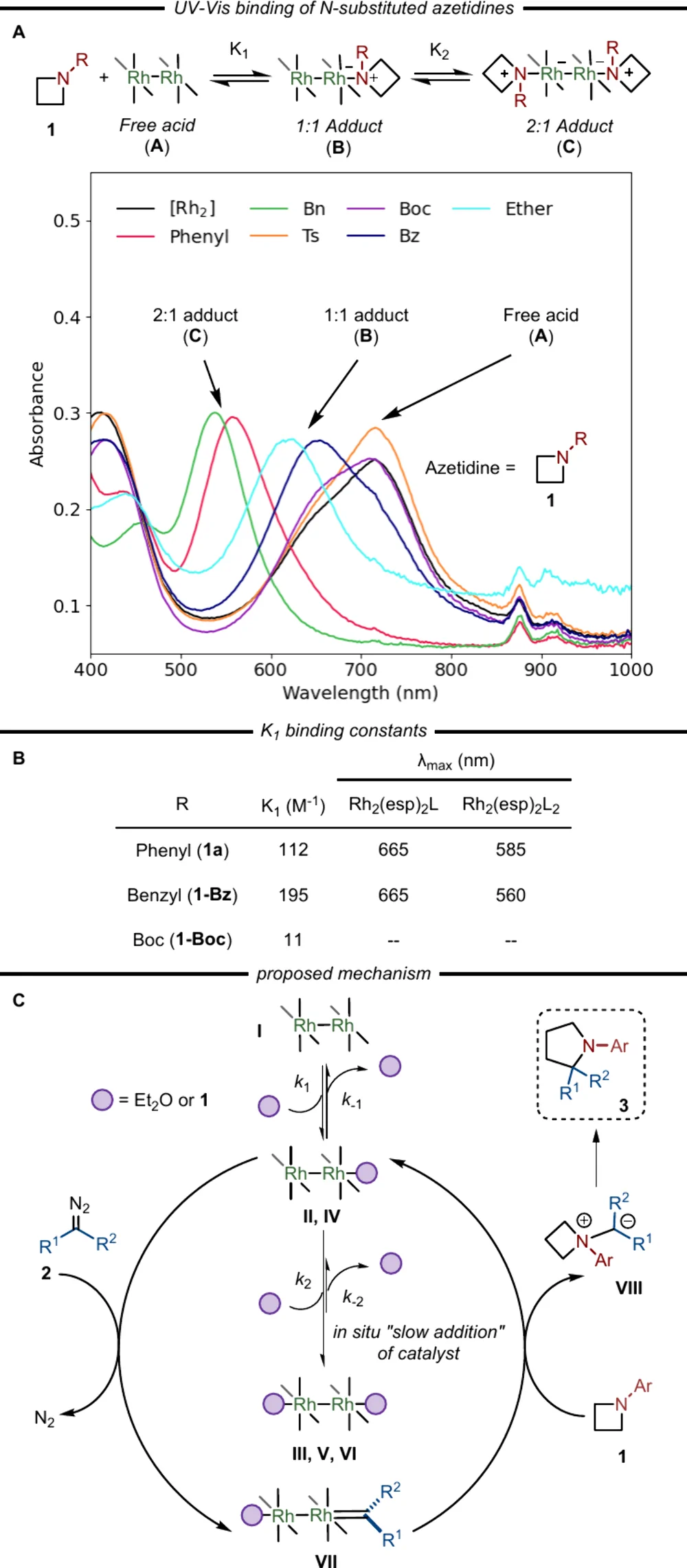
(A) UV-Vis spectrum of adducts of Rh_2_(esp)_2_ and protected azetidines. [Rh_2_(esp)_2_] = 1.1 mM and [azetidine] = 10.0 mM. (B) Calculated *K*_1_ values for complex formation between **1a**, 1-Bz, and 1-Boc with Rh_2_(esp)_2_ in PhMe at rt, with wavelengths (nm) of 2:1 and 1:1 Rh_2_(esp)_2_:azetidine adducts from UV–Vis titration. (C) Proposed mechanism.

Equilibrium constants (*K*_1_) for the association of Ph-, Bn-, and Boc-substituted azetidines with Rh_2_(esp)_2_ corroborate these findings, where *K*_1_ is a measure of the reactivity of Rh_2_(esp)_2_ toward **1a** (Figure 3B and Figures S1-12–S1-14).^17,18,20^ The *N*-Boc azetidine exhibits a low *K*_1_ (*K*_1_ = 11 M^–1^), indicating reduced azetidine basicity that suppresses catalyst coordination and permits unchecked carbene dimerization. In contrast, the increased basicity of the *N*-Bn azetidine leads to strong Rh binding (*K*_1_ = 195 M^–1^) that inhibits **2a** reactivity. Ph-protected azetidine **1a** lies between these two extremes, where strong, yetreversible Rh binding impedes competing carbene dimerization and promotes formation of the product.

Equipped with these insights, a potential mechanism was proposed to account for the success without slow carbene addition (Figure 3C). Rh_2_(esp)_2_ (**I**) undergoes a reversible binding to *N*-aryl azetidine **1** and/or Et_2_O; at high concentrations of **1**, strong **1**-Rh binding favors the diligated complexes **III**, **V**, and/or **VI**. Slow dissociation of **1** or Et_2_O furnishes active catalysts **II**/**IV**. We hypothesize that this equilibrium acts as an *in situ* “slow addition” of **II**/**IV**, where monoligated species coordinate with carbene precursor **2** and undergo N_2_ extrusion to furnish **VII**. The low concentrations of **I** and **II**/**IV** prevent dimerization of **VII**, a competing pathway that typically necessitates slow addition of **2**. Nucleophilic attack by azetidine **1** on carbenoid **VII** and dissociation of Rh give azetidinium ylide intermediate **VIII**, which undergoes ring expansion to pyrrolidine **3**.

With optimized conditions and a mechanistic understanding in hand, the scope of the reaction was explored (Table 1).^26^ An *N*-*p*-methoxyphenyl (PMP) group was employed on the azetidine throughout the scope, as this protecting group both facilitated purification and could be easily removed (*vide infra*). Aryl diazoesters bearing both electron-donating and -withdrawing substituents delivered pyrrolidines **3aa**–**3bg** in good yields. Diazoesters bearing heteroarenes also underwent successful ring expansion to give **3bh** and **3bi** in good yields. The donor group of the diazoester could be extended to alkenyl (**3bj**) and alkyl (**3bk**) substitutions, while the acceptor group could be modified to give ketone- (**3bl**) or trifluoromethyl- (**3bm**) substituted pyrrolidines. Finally, the use of cyclic diazoesters gave **3bn** and **3bo** in moderate to good yields, where the oxindole spiropyrrolidine motif of **3bo** has been shown to have bioactivity against cancer cell lines.^27^

**Table 1:**
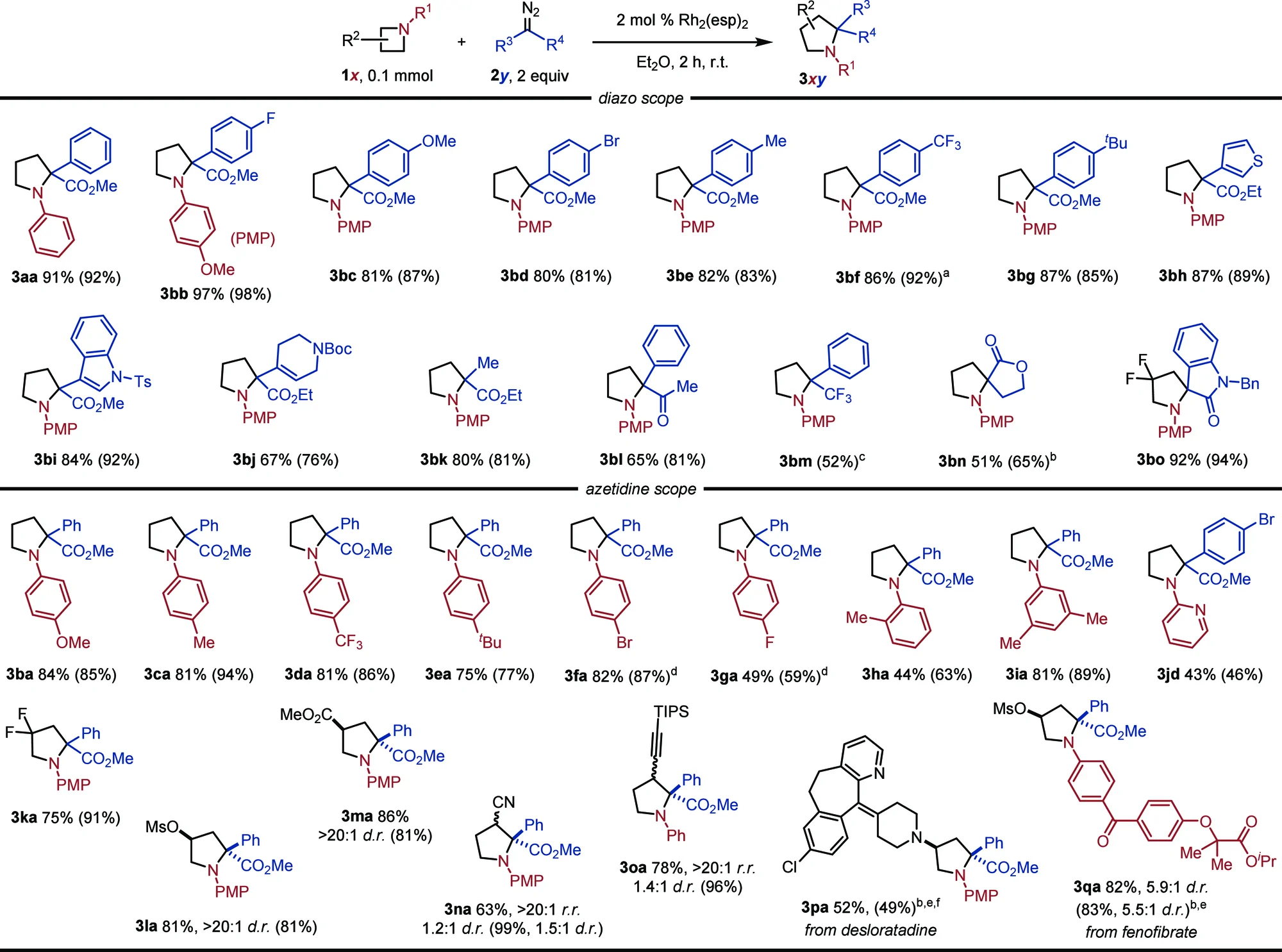
Scope of the Rh-Catalyzed Ring Expansion of Azetidines to Pyrrolidines^a^

* ^1^H NMR yields in parentheses were calculated using mesitylene as an internal standard. dr = diastereomeric ratio, and rr = regioisomeric ratio.

a Reaction time = 3 h.

b A total of 3 equiv of diazo.

c The product could not be isolated cleanly.

d The reaction was carried out in 1,4-dioxane to improve isolation.

e At 24 h of reaction time.

f *dr* was not determined due to peak overlap in the ^1^H NMR.

In terms of the azetidine scope, electronically diverse *N*-aryl azetidines delivered **3ba**–**3ga** in moderate to good yields. The reaction was sensitive to sterics, as an *o*-methylphenyl-substituted azetidine gave **3ha** in a moderate yield, while a 2,6-dimethylphenyl-substituted azetidine gave no reaction (see Figure S1-11 for further details). However, reactivity was restored with *N*-3,5-dimethylphenyl-substituted azetidine (**3ia**). An *N*-heterocyclic azetidine (**3jd**) was successful despite the potential for competitive 2-pyridyl catalyst binding. Gratifyingly, C3 azetidine substitutions were well-tolerated, and sensitive difluoro (**3ka**), mesylate (**3la**), and ester (**3ma**) groups all gave pyrrolidines in good yields and excellent *dr* for **3la** and **3ma**. While the reaction was sensitive to steric congestion in C2-substituted azetidines, nitrile (**3na**) and alkynyl (**3oa**) substituents at C2 gave pyrrolidines in good yields with excellent regioselectivity, albeit in low *dr*. Finally, azetidines derived from complex scaffolds underwent ring expansion in moderate to good yields. A pyrrolidine derived from the antihistamine desloratadine (**3pa**) was obtained despite the presence of two competitively binding *N*-heterocycles, while a substrate derived from fenofibrate yielded a pyrrolidine-modified structure (**3qa**) in a good yield and *dr*.

The products were amenable to diverse post-functionalizations (Figure 4A). For example, *N*-PMP pyrrolidine **3ba** was easily deprotected in 62% yield using ceric ammonium nitrate (CAN) and in 67% yield with trichloroisocyanuric acid (TCCA). A one-pot procedure using CAN gave **4ba** from **1b** in a comparable yield. Alkyne-containing **3oa** was easily modified for further reactivity by the removal of the TIPS protecting group to furnish **4oa**. Reduction of **3bd** gave **4bd** in a good yield. Mesylate-substituted **3la** was transformed to 4-azidopyrrolidine **4la** with perfect retention of stereochemical information. The ring expansion was suitably robust at 1 mmol scale to give **3ba** and **3la** in good yields using only 0.2 mol % catalyst (1.5 mg) (Figure 4B). Finally, to shed insights into the possible mechanism of the ring expansion, a chirality transfer experiment was conducted with enantioenriched (*S*)-1-(4-methoxyphenyl)-azetidine-2-carbonitrile **1l** (Figure 4C). A minor reduction in *ee* from 99 to 89% in **3la** was noted, a change consistent with a potential homolytic cleavage of the C2–N bond, followed by rapid recombination of solvent-caged radical intermediates that largely conserves the precursor *ee*, similar to reports on [1,2]-Stevens rearrangements.^11,28^

**Figure 4:**
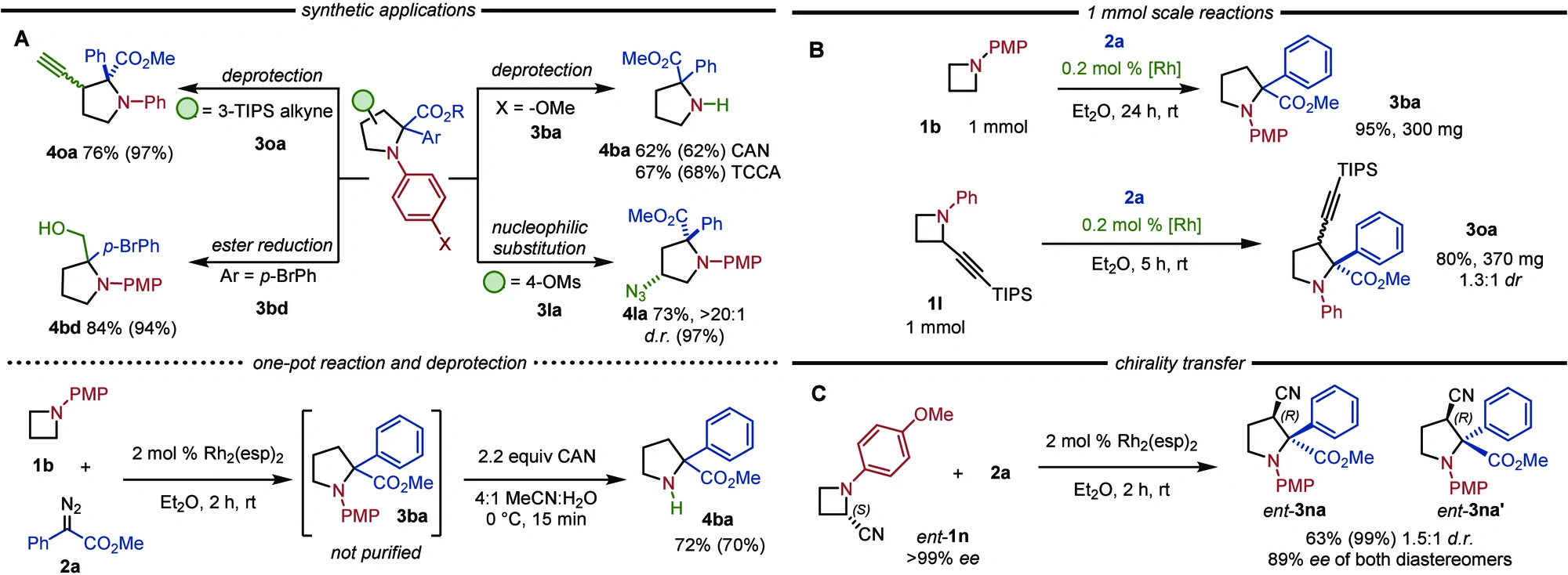
Derivatizations of pyrrolidines and stereochemical probe.

In conclusion, we have developed a modular method for the intermolecular syntheses of *N*-aryl pyrrolidines from azetidines and diverse carbene precursors. UV–vis studies suggest a key binding equilibrium between the azetidine and catalyst that prevents rapid dimerization of carbenoid intermediates and the need for slow addition of the diazoester. The reaction conditions are mild and tolerate a wide array of functional groups, including those in complex molecule settings. The products are easily derivatized to drug-like pyrrolidines. Stereochemical probe experiments highlight the potential diradical nature of the ring expansion, while demonstrating that chiral information can be transmitted from the precursor to the product. Potential applications of this *in situ* “catalyst-protection” strategy to other metal-catalyzed carbene transformations are underway, as additives capable of reversibly binding the catalyst and suppressing carbene dimerization could obviate the need for slow addition in a broader range of group transfer chemistries.

## Supplementary Material





## Data Availability

The data underlying this study are available in the published article and its .

## Notes

UV–vis adduct study (XLSX)
Copies of NMR spectra of all compounds, details of the kinetic experiments, and details of the chirality transfer experiments (DOCX)
