# Supplementary material for: Rh-Catalyzed Expansion of Azetidines to Pyrrolidines via Modulated Carbene Transfer
Source: Org Lett. 2026 Oct 17;28(43):13396–401. doi: 10.1021/acs.orglett.6c03625 (PMC13729706; doi:10.1021/acs.orglett.6c03625)
Supplement: Supplementary file 1 [file ol6c03625_si_002.docx]

**Supporting Information** **1**

**Rh-catalyzed expansion of azetidines to pyrrolidines via modulated carbene transfer**

Olivia M. Duke,^1^ Jensen J. Zerban,^1^ Xiang Lian,^1^ Jennifer M. Schomaker^1,^*

*^1^Department of Chemistry, University of Wisconsin, 1101 University Avenue, Madison, WI 53706*

Table of Contents

I. [General Information	S1-2](#_Toc237564806)
II. [Preparation and Characterization of Azetidine Starting Materials	S1-3](#_Toc237564807)
III. [Preparation and Characterization of Diazo Starting Materials	S1-16](#_Toc237564808)
IV. [Preparation and Characterization of Pyrrolidine Products	S1-16](#_Toc237564809)
V. [Procedures for Post-Functionalization and Characterization of Products	S1-38](#_Toc237564810)
VI. [Chirality Transfer Studies	S1-43](#_Toc237564811)
VII. [Details of Rh-Azetidine Binding UV-Vis Experiments	S1-48](#_Toc237564812)
VIII. [Equilibrium Constant Determination for Rh_2_(esp)_2_ and Protected Azetidines	S1-51](#_Toc237564813)
IX. [Detailed Proposed Mechanism	S1-57](#_Toc237564814)
X. [References	S1-58](#_Toc237564815)
XI. [NMR	S1-61](#_Toc237564816)

# I. General Information

Unless otherwise specified, all reactions were run under an inert atmosphere of N_2_. Glassware was either oven-dried overnight at 130 °C or flame-dried under a stream of dry nitrogen prior to use. Unless otherwise specified, reagents were used as obtained from the vendor without further purification. Procedures for compounds requiring purification were obtained from “Purification of Laboratory Chemicals”.^1^ All standard solvents (diethyl ether, 1,4-dioxane, acetonitrile, dichloromethane, toluene) were obtained from a PureSolv MD5 5 column solvent purification system. All other solvents were also purified in accordance with methods reported in "Purification of Laboratory Chemicals”.^1^ Analytical thin layer chromatography (TLC) was performed utilizing pre-coated silica gel 60 F_254_ plates containing a fluorescent indicator, while preparative chromatography was performed using SilicaFlash P60 silica gel (230-400 mesh) via Still’s method.^2^ The mobile phases for column chromatography varied depending on the substrate; however, hexanes/diethyl ether or hexanes/ethyl acetate were commonly employed. Columns were typically run using a gradient method, beginning with 100% of the less polar eluent and gradually increasing the polarity with the other solvent. For reactions producing products without a UV signature, a potassium permanganate or phosphomolybdic acid (PMA) stain was employed to visualize the reaction products.

^1^H NMR, ^13^C NMR, and ^19^F spectra were obtained using Bruker Avance-**400**, Bruker Avance-500, or Varian Inova-600 NMR spectrometers. For ^1^H NMR, chemical shifts are reported relative to either tetramethylsilane (TMS) or residual protiated solvent peaks (δ 7.26, 2.49, 7.15 and 4.80 ppm for CDCl_3_, (CD_3_)_2_SO, C_6_D_6_ and CD_3_OD respectively). ^13^C NMR spectra were measured at either 125 MHz or 150 MHz on the same instruments noted above for recording ^1^H NMR spectra. Chemical shifts were again reported in accordance with residual solvent peaks (δ 77.1, 39.5, 128.0 and 49.0 ppm for CDCl_3_, (CD_3_)_2_SO, C_6_D_6,_ and CD_3_OD, respectively). ^19^F NMR spectra were measured at 377 MHz on a Bruker Avance-400 spectrometer. Accurate mass measurements were acquired at the University of Wisconsin, Madison using a Thermo Q Exactive™ Plus (electrospray ionization, time-of-flight analyzer, or electron impact methods). The Paul Bender Chemistry Instrumentation Center (NMR and mass spectrometry) is funded by the NSF (CHE-1048642), the NIH (1S10 OD020022-1 & S10 OD012245), the Bender Fund, UW2020, and the University of Wisconsin-Madison.

# II. Preparation and Characterization of Azetidine Starting Materials

**Figure S1-1**. Azetidine precursors.

*General Procedure A:* Azetidines were prepared through a copper-catalyzed cross-coupling adapted from a reported literature procedure.^3^ A flame-dried two-neck round bottom flask was fitted with a stir bar, reflux condenser, and septa, and cooled under vacuum. The flask was flushed with N_2_, and CuI (0.1 equiv) and K_3_PO_4_ (3 equiv) were added. The flask was flushed with N_2_ three times, followed by the addition of butanol (0.7 M), ethylene glycol (2.5 equiv), azetidine (1.1 equiv), and iodobenzene (13.0 mmol) via syringe. The mixture was heated to 100 °C in a bead or sand bath and stirred for 24 h. After 24 h, the reaction was cooled to room temperature and quenched with saturated NH_4_Cl, then extracted with Et_2_O three times. The organic layers were combined, dried with MgSO_4_, and filtered. The filtrate was concentrated in vacuo and further purified through silica gel column chromatography in a gradient from 100% hexanes to 10% Et_2_O in hexanes.

*General Procedure B:* Azetidines were prepared through a copper-catalyzed cross-coupling adapted from reported literature procedures.^4,5^ A flame-dried two-neck round bottom flask was fitted with a stir bar, reflux condenser, and septa, and cooled under vacuum. The flask was flushed with N_2_, and CuI (0.17 equiv), K_2_CO_3_ (4 equiv), and L-proline (0.34 equiv) were added. The flask was flushed with N_2_ three times, followed by the addition of DMSO (0.44 M), azetidine (10.0 mmol), and 2-iodotoluene (1.25 equiv) via syringe. The mixture was heated to 90 °C in a bead or sand bath and stirred overnight. The reaction was cooled to room temperature and extracted with CH_2_Cl_2_ three times. The organic layers were combined, dried with MgSO_4_, and filtered. The filtrate was concentrated in vacuo and purified through silica gel column chromatography in a gradient from 100% hexanes to 10% Et_2_O in hexanes.

**1a 1-phenylazetidine:** Synthesis of **1a** was conducted on 13 mmol scale according to *General Procedure A* to give **1a** as a pale yellow oil (1.1 g, 60%). ^1^H NMR (CDCl_3_, 500 MHz): δ 7.25 (t, *J* = 7.9 Hz, 2H), 6.77 (t, *J* = 7.3 Hz, 1H), 6.50 (d, *J* = 8.5 Hz, 2H), 3.92 (t, *J* = 7.2 Hz, 4H), 2.40 (p, *J* = 7.2 Hz, 2H). ^13^C NMR (CDCl_3_, 125 MHz): δ 152.3, 128.8, 120.4, 117.3, 111.3, 52.4, 17.0. HRMS (ESI) *m/z* calculated for C_9_H_12_N^+^ [M+H]^+^ 134.0964; found: 134.0965.

**1b 1-(4-methoxyphenyl)azetidine:** Synthesis of **1b** was conducted on 10 mmol scale according to *General Procedure A* to give **1b** as a pale yellow oil (1.29 g, 79%). ^1^H NMR (CDCl_3_, 500 MHz): δ 6.84 – 6.77 (m, 2H), 6.46 – 6.39 (m, 2H), 3.80 (t, *J* = 7.1 Hz, 4H), 3.75 (s, 3H), 2.32 (p, *J* = 7.2 Hz, 2H). ^13^C NMR (CDCl_3_, 125 MHz): δ 152.1, 147.2, 114.6, 112.6, 55.8, 53.1, 17.1. HRMS (ESI) *m/z* calculated for C_10_H_14_NO^+^ [M+H]^+^ 164.1070; found: 164.1070.

**1c 1-(p-tolyl)azetidine:** Synthesis of **1c** was conducted on a 5 mmol scale according to *General Procedure A* to give **1c** as a pale yellow oil (0.46 g, 63%). **^1^H NMR (CDCl_3_, 500 MHz): δ 7.15 – 6.87 (m, 2H), 6.51 – 6.20 (m, 2H), 3.83 (t, *J* = 7.1 Hz, 4H), 2.33 (p, *J* = 7.1 Hz, 2H), 2.25 (s, 3H).** ^13^C NMR (CDCl_3_, 125 MHz): δ 150.4, 129.4, 126.5, 111.6, 52.8, 20.4, 17.1. HRMS (ESI) *m/z* calculated for C_10_H_14_N^+^ [M+H]^+^ 148.1121; found: 148.1119.

**1d 1-(4-(trifluoromethyl)phenyl)azetidine:** Synthesis of **1d** was conducted on 5 mmol scale according to *General Procedure A* to give **1d** as a white solid (0.35 g, 35%). ^1^H NMR (CDCl_3_, 500 MHz): δ 7.25 (d, *J* = 9.0 Hz, 2H), 6.24 (d, *J* = 8.6 Hz, 2H), 3.77 (t, *J* = 7.3 Hz, 4H), 2.24 (p, *J* = 7.6, 7.4 Hz, 2H). ^13^C NMR (CDCl_3_, 125 MHz): δ 153.8, 126.2 (q, *J* = 3.8 Hz), 125.1 (q, *J* = 270.4 Hz), 118.4 (q, *J* = 32.5 Hz), 110.2, 51.9, 16.7. ^19^F NMR (CDCl_3_, 377 MHz): δ -60.9. HRMS (ESI) *m/z* calculated for C_10_H_11_F_3_N^+^ [M+H]^+^ 202.0838; found: 202.0838.

**1e 1-(4-(tert-butyl)phenyl)azetidine:** Synthesis of **1e** was conducted on 5 mmol scale according to *General Procedure A* to give **1e** as a white solid (0.40 g, 47%). ^1^H NMR (CDCl_3_, 500 MHz): δ 7.27 – 7.23 (m, 2H), 6.46 – 6.40 (m, 2H), 3.86 (t, *J* = 7.2 Hz, 4H), 2.34 (p, *J* = 7.1 Hz, 2H), 1.29 (s, 9H). ^13^C NMR (CDCl_3_, 125 MHz): δ 150.2, 140.0, 125.6, 125.4, 111.1, 52.6, 33.9, 31.6, 17.1. HRMS (ESI) *m/z* calculated for C_13_H_20_N^+^ [M+H]^+^ 190.1590; found: 190.1590.

**1f 1-(4-bromophenyl)azetidine:** Synthesis of **1f** was conducted on 5 mmol scale according to *General Procedure A* to give **1f** as a white solid (0.41 g, 39%). ^1^H NMR (CDCl_3_, 500 MHz): ^1^H NMR (CDCl_3_, 500 MHz): δ 7.28 – 7.22 (m, 2H), 6.32 – 6.25 (m, 2H), 3.82 (t, *J* = 7.2 Hz, 4H), 2.34 (p, *J* = 7.2, 7.0, 6.9 Hz, 2H). ^13^C NMR (CDCl_3_, 125 MHz): δ 151.1, 131.6, 112.9, 109.2, 52.4, 16.8. HRMS (ESI) *m/z* calculated for C_9_H_10_BrN^+^ [M+H]^+^ 212.0069; found: 212.0071.

**1g 1-(4-fluorophenyl)azetidine:** Synthesis of **1g** was conducted on 5 mmol scale according to *General Procedure A* to give **1g** as a pale yellow oil (0.45 g, 59%). ^1^H NMR (CDCl_3_, 500 MHz): δ 6.97 – 6.81 (m, 2H), 6.43 – 6.29 (m, 2H), 3.82 (t, *J* = 7.1 Hz, 4H), 2.34 (p, *J* = 7.1 Hz, 2H). ^13^C NMR (CDCl_3_, 125 MHz): δ 156.0 (d, *J* = 235 Hz), 149.0 (d, *J* = 1.8 Hz), 115.3 (d, *J* = 22.5 Hz), 112.2 (d, *J* = 7.7 Hz), 52.9, 16.9. ^19^F NMR (CDCl_3_, 377 MHz): δ -128.2. HRMS (ESI) *m/z* calculated for C_9_H_11_FN^+^ [M+H]^+^ 152.0870; found: 152.0870.

**1h 1-(o-tolyl)azetidine:** Synthesis of **1h** was conducted on 10 mmol scale according to *General Procedure A* to give **1h** as a pale yellow oil (0.33 g, 23%). ^1^H NMR (CDCl_3_, 500 MHz): δ 7.14 – 7.07 (m, 1H), 7.05 – 6.99 (m, 1H), 6.75 (td, *J* = 7.3, 1.2 Hz, 1H), 6.49 (dd, *J* = 8.0, 1.2 Hz, 1H), 3.91 (t, *J* = 7.1 Hz, 4H), 2.27 (p, *J* = 7.1 Hz, 2H), 2.22 (s, 3H). ^13^C NMR (CDCl_3_, 125 MHz): δ 150.6, 131.3, 126.4, 124.9, 119.2, 112.8, 53.9, 19.6, 17.2. HRMS (ESI) *m/z* calculated for C_10_H_14_N^+^ [M+H]^+^ 148.1121; found: 148.1121.

**1i 1-(3,5-dimethylphenyl)azetidine:** Synthesis of **1i** was conducted on 10 mmol scale according to *General Procedure B* to give **1i** as a pale yellow oil (0.98 g, 61%). ^1^H NMR (CDCl_3_, 500 MHz): δ 6.41 (s, 1H), 6.12 – 6.08 (m, 2H), 3.85 (t, *J* = 7.1 Hz, 4H), 2.34 (p, *J* = 7.1 Hz, 2H), 2.27 (s, 6H). ^13^C NMR (CDCl_3_, 125 MHz): δ 152.5, 138.5, 119.4, 109.2, 52.5, 21.5, 17.0. HRMS (ESI) *m/z* calculated for C_11_H_15_N^+^ [M+H]^+^ 162.1277; found: 162.1278.

**1j 2-(azetidin-1-yl)pyridine:** Synthesis of **1j** was conducted on 2.5 mmol scale according to an adapted literature procedure^6^ to give **1j** as a pale yellow oil (0.20 g, 61%). ^1^H NMR (CDCl_3_, 500 MHz): δ 8.14 (d, *J* = 5.0 Hz, 1H), 7.42 (ddd, *J* = 8.6, 7.0, 1.7 Hz, 1H), 6.56 (dd, *J* = 7.1, 5.1 Hz, 1H), 6.25 (d, *J* = 8.3 Hz, 1H), 4.06 – 3.99 (m, 4H), 2.43 – 2.33 (m, 2H). ^13^C NMR (CDCl_3_, 125 MHz): δ 160.9, 148.2, 136.9, 112.5, 105.6, 50.7, 16.7. HRMS (ESI) *m/z* calculated for C_8_H_11_N_2_^+^ [M+H]^+^ 135.0917; found: 135.0917.

**1k 3,3-difluoro-1-(4-methoxyphenyl)azetidine:** Synthesis of **1k** was conducted on 5 mmol scale from 3,3-difluoroazetidine hydrochloride according to an adapted literature procedure^7^ to give **1k** as a white solid (0.89 g, 89%). ^1^H NMR (CDCl_3_, 500 MHz): δ 6.84 (d, *J* = 8.8 Hz, 2H), 6.47 (d, *J* = 8.8 Hz, 2H), 4.15 (t, *J* = 11.7 Hz, 4H), 3.76 (s, 3H). ^13^C NMR (CDCl_3_, 125 MHz): δ 153.2, 144.3 (t, *J* = 2.5 Hz), 115.9 (t, *J* = 274.7 Hz), 114.8, 113.6, 63.8 (t, *J* = 24.9 Hz), 55.8. ^19^F NMR (CDCl_3_, 377 MHz): δ -99.2. HRMS (ESI) *m/z* calculated for C_10_H_12_F_2_NO^+^ [M+H]^+^ 200.0882; found: 200.0883.

Azetidine **1l** was synthesized in two steps according to adapted literature procedures.

**S1-1l 1-(4-methoxyphenyl)azetidin-3-ol:** Synthesis of **S1-1l** was conducted on 30 mmol scale from azetidin-3-ol hydrochloride according to *General Procedure B* to give S1-**1l** as a white solid (3.6 g, 66%). ^1^H NMR (CDCl_3_, 500 MHz): δ 6.84 – 6.78 (m, 2H), 6.48 – 6.41 (m, 2H), 4.71 (br s, 1H), 4.13 (t, *J* = 6.7 Hz, 2H), 3.75 (s, 3H), 3.59 (dd, *J* = 8.4, 4.6 Hz, 2H), 2.00 (br s, 1H). ^13^C NMR (CDCl_3_, 125 MHz): δ 152.5, 146.3, 114.7, 113.2, 62.9, 62.5, 55.8. HRMS (ESI) *m/z* calculated for C_10_H_13_NO_2_^+^, (M+H)^+^ 180.1019; found 180.1021.

**1l 1-(4-methoxyphenyl)azetidin-3-yl methanesulfonate:** Synthesis of **1l** was conducted on 3 mmol scale from 1-(4-methoxyphenyl)azetidin-3-ol S1**-1l** according to an adapted literature procedure^8^ to give **1l** as a white solid (0.63 g, 80%). ^1^H NMR (CDCl_3_, 500 MHz): δ 6.86 – 6.79 (m, 2H), 6.47 – 6.41 (m, 2H), 5.36 (tt, *J* = 6.1, 4.5 Hz, 1H), 4.22 (ddd, *J* = 7.5, 6.2, 1.2 Hz, 2H), 3.91 (ddd, *J* = 8.0, 4.6, 1.2 Hz, 2H), 3.75 (s, 3H), 3.06 (s, 3H). ^13^C NMR (CDCl_3_, 125 MHz): δ 153.0, 145.4, 114.8, 113.2, 68.2, 59.6, 55.8, 38.4. HRMS (ESI) *m/z* calculated for C_11_H_16_NO_4_S^+^ [M+H]^+^ 258.0795; found: 258.0792.

Azetidine **1m** was synthesized in two steps according to an adapted literature procedure and *General Procedure B*.^9^

**S1-1m methyl azetidine-3-carboxylate hydrochloride:** Synthesis of S1-**1m** was conducted on 5 mmol scale from azetidine-3-carboxylic acid according to an adapted literature procedure^9^ to give S1-**1m** as a sticky yellow solid. The crude product was used without further purification, and the yield was presumed to be quantitative for the following reaction.

**1m methyl 1-(4-methoxyphenyl)azetidine-3-carboxylate:** Synthesis of **1m** was conducted on 5 mmol scale from methyl azetidine-3-carboxylate hydrochloride **S1-1m** according to *General Procedure B* to give **1m** as a white solid (0.47 g, 42%). ^1^H NMR (CDCl_3_, 500 MHz): δ 6.85 – 6.78 (m, 2H), 6.47 – 6.40 (m, 2H), 4.03 (dd, *J* = 8.3, 6.8 Hz, 2H), 3.95 (t, *J* = 6.5 Hz, 2H), 3.74 (d, *J* = 4.0 Hz, 6H), 3.53 (tt, *J* = 8.3, 6.2 Hz, 1H). ^13^C NMR (CDCl_3_, 125 MHz): δ 173.3, 152.6, 146.1, 114.7, 112.8, 55.8, 55.1, 52.1, 33.6. HRMS (ESI) *m/z* calculated for C_12_H_16_NO_3_^+^ [M+H]^+^ 222.1125; found: 222.1123.

**1n 1-(4-methoxyphenyl)azetidine-2-carbonitrile:** Azetidine **1n** was synthesized according to reported literature procedure^10^ on 2.3 mmol scale to give **1n** as a white solid (38 mg, 9%). Product characterization matched reported literature values.^10^

Azetidine **1o** was synthesized in four steps according to published literature procedures.^11,12^

**S1-1o 3-(triisopropylsilyl)propiolaldehyde:** S1-**1o** was synthesized according to published literature procedure^11^ on 5 mmol scale and purified by flash column chromatography (gradient from 100% hexanes to 50% ethyl acetate:hexanes) to give S1-**1o** as a colorless oil (0.68 g, 65%). Product characterization matched reported literature values.^11^

**S2-1o 3-hydroxy-*N*-phenyl-5-(triisopropylsilyl)pent-4-ynamide:** S2-**1o** was synthesized according to an adapted literature procedure^12^ on 3.2 mmol scale and purified through column chromatography (gradient from 99% hexanes:1% Et_3_N to 59% hexanes:40% EtOAc:1% Et_3_N) to give S2-**1o** as a white solid **(**0.73 g, 66%). ^1^H NMR (CDCl_3_, 500 MHz): δ 7.59 (br s, 1H), 7.43 (d, *J* = 7.9 Hz, 2H), 7.26 (t, *J* = 7.9 Hz, 2H), 7.05 (t, *J* = 7.4 Hz, 1H), 4.81 (dd, *J* = 7.5, 3.6 Hz, 1H), 3.21 (br s, 1H), 2.78 (dd, *J* = 15.4, 3.7 Hz, 1H), 2.70 (dd, *J* = 15.4, 7.4 Hz, 1H), 0.99 – 0.95 (m, 21H). ^13^C NMR (CDCl_3_, 125 MHz): δ 168.6, 129.0, 124.6, 120.1, 106.4, 86.9, 59.8, 44.6, 18.5, 11.0. HRMS (ESI) *m/z* calculated for C_20_H_32_NO_2_Si^+^, (M+H)^+^ 346.2197; found 346.2197.

**S3-1o 1-phenyl-4-((triisopropylsilyl)ethynyl)azetidin-2-one:** S3-**1o** was synthesized according to an adapted literature procedure^12^ on 2.1 mmol scale and purified through silica gel column chromatography (gradient 100% petroleum ether to 10% ether:petroleum ether) to give S3-**1o** as a white solid (0.50 g, 71%). ^1^H NMR (CDCl_3_, 500 MHz): δ 7.56 – 7.50 (m, 2H), 7.36 – 7.29 (m, 2H), 7.10 (tt, *J* = 7.4, 1.1 Hz, 1H), 4.60 (dd, *J* = 5.6, 2.7 Hz, 1H), 3.43 (dd, *J* = 14.8, 5.6 Hz, 1H), 3.16 (dd, *J* = 14.8, 2.8 Hz, 1H), 1.09 – 0.98 (m, 21H). ^13^C NMR (CDCl_3_, 125 MHz): δ 163.2, 137.4, 129.0, 124.1, 116.7, 102.8, 89.0, 44.9, 41.1, 18.5, 11.0. HRMS (ESI) *m/z* calculated for C_20_H_30_NOSi^+^, (M+H)^+^ 328.2091; found 328.2090.

**1o 1-phenyl-2-((triisopropylsilyl)ethynyl)azetidine: 1o** was synthesized according to an adapted literature procedure^12^ on 1.5 mmol scale to give **1o** as a white solid (0.48 g, quant.). ^1^H NMR (CDCl_3_, 500 MHz): δ 7.21 (t, *J* = 8.4, 7.4 Hz, 2H), 6.78 (tt, *J* = 7.3, 1.1 Hz, 1H), 6.66 (d, *J* = 7.8 Hz, 2H), 4.53 (t, *J* = 7.6 Hz, 1H), 3.90 (td, *J* = 7.1, 5.0 Hz, 1H), 3.62 (td, *J* = 7.9, 6.6 Hz, 1H), 2.59 – 2.49 (m, 2H), 1.08 – 1.04 (m, 21H). ^13^C NMR (CDCl_3_, 125 MHz): δ 151.3, 128.7, 118.4, 112.4, 107.6, 86.4, 54.0, 49.8, 25.6, 18.6, 11.2. HRMS (ESI) *m/z* calculated for C_20_H_32_NSi^+^, (M+H)^+^ 314.2298; found 314.2298.

Azetidine **1p** was synthesized according to *General Procedure B* and adapted literature procedures.^8,13^

**1p 8-chloro-11-(1-(1-(4-methoxyphenyl)azetidin-3-yl)piperidin-4-ylidene)-6,11-dihydro-5H-benzo[5,6]cyclohepta[1,2-b]pyridine:** Synthesis of **1p** was conducted on 0.6 mmol scale from 1-(4-methoxyphenyl)azetidin-3-yl methanesulfonate **1l** according to an adapted literature procedure^13^ to give **1p** as a pink solid (0.14 g, 49%). ^1^H NMR (CDCl_3_, 500 MHz): δ 8.39 (dd, *J* = 4.7, 1.6 Hz, 1H), 7.42 (dd, *J* = 7.6, 1.6 Hz, 1H), 7.17 – 7.10 (m, 3H), 7.08 (dd, *J* = 7.6, 4.7 Hz, 1H), 6.81 – 6.74 (m, 2H), 6.44 – 6.38 (m, 2H), 3.90 (q, *J* = 6.5, 6.3 Hz, 2H), 3.73 (s, 3H), 3.62 (t, *J* = 6.3 Hz, 2H), 3.45 – 3.31 (m, 2H), 3.27 (p, *J* = 6.2 Hz, 1H), 2.88 – 2.74 (m, 2H), 2.69 (tt, *J* = 10.8, 4.6 Hz, 2H), 2.53 (ddd, *J* = 14.0, 9.7, 4.2 Hz, 1H), 2.47 – 2.30 (m, 3H), 2.15 – 2.05 (m, 2H). ^13^C NMR (CDCl_3_, 125 MHz): δ 157.5, 152.2, 146.7, 146.6, 139.5, 138.4, 137.8, 137.3, 133.4, 133.0, 132.7, 130.8, 129.0, 126.0, 122.1, 114.6, 112.8, 56.8, 56.8, 55.8, 55.1, 51.4, 51.3, 31.8, 31.5, 30.6, 30.4, 29.7. HRMS (ESI) *m/z* calculated for C_29_H_31_ClN_3_O^+^, (M+H)^+^ 472.2150; found 472.2152.

Azetidine **1q** was synthesized according to adapted literature procedures.^8,14^

**S1-1q isopropyl 2-(4-(4-(3-hydroxyazetidin-1-yl)benzoyl)phenoxy)-2-methylpropanoate:** Synthesis of S1-**1q** was conducted on 2 mmol scale from azetidin-3-ol hydrochloride according to an adapted literature procedure^14^ and purified through silica gel column chromatography (gradient 100% hexanes to 40% ethyl acetate:hexanes) to give S1-**1q** as a yellow oil (0.29 g, 36%). Only 0.05 equiv of the palladium catalyst was used instead of 0.2 equiv as reported in the literature. ^1^H NMR (CDCl_3_, 500 MHz): δ 7.73 – 7.68 (m, 2H), 7.68 – 7.64 (m, 2H), 6.87 – 6.81 (m, 2H), 6.41 – 6.35 (m, 2H), 5.08 (hept, *J* = 6.2 Hz, 1H), 4.83 – 4.77 (m, 1H), 4.28 – 4.21 (m, 2H), 3.81 (dd, *J* = 8.7, 4.3 Hz, 2H), 2.53 (s, 1H), 1.64 (s, 6H), 1.20 (d, *J* = 6.2 Hz, 6H). ^13^C NMR (CDCl_3_, 125 MHz): δ 194.4, 173.4, 158.6, 153.7, 132.3, 132.1, 131.4, 126.5, 117.3, 110.1, 79.3, 69.2, 62.5, 61.1, 25.4, 21.5. HRMS (ESI) *m/z* calculated for C_23_H_28_NO_5_^+^, (M+H)^+^ 398.1962; found 398.1962.

**1q isopropyl 2-methyl-2-(4-(4-(3-((methylsulfonyl)oxy)azetidin-1-yl)benzoyl)phenoxy)propanoate:** Synthesis of **1q** was conducted on 0.4 mmol scale from isopropyl 2-(4-(4-(3-hydroxyazetidin-1-yl)benzoyl)phenoxy)-2-methylpropanoate S1**-1q** according to an adapted literature procedure^8^ to give **1q** as a sticky yellow oil (0.15 g, 78%). ^1^H NMR (CDCl_3_, 500 MHz): δ 7.77 – 7.70 (m, 2H), 7.70 – 7.64 (m, 2H), 6.89 – 6.82 (m, 2H), 6.47 – 6.41 (m, 2H), 5.42 (tt, *J* = 6.3, 4.1 Hz, 1H), 5.08 (hept, *J* = 6.2, 6.1 Hz, 1H), 4.38 (dd, *J* = 9.4, 7.1 Hz, 2H), 4.13 (dd, *J* = 9.1, 3.9 Hz, 2H), 3.10 (s, 3H), 1.65 (s, 6H), 1.20 (d, *J* = 6.1 Hz, 6H). ^13^C NMR (CDCl_3_, 125 MHz): δ 194.2, 173.3, 158.8, 152.9, 132.2, 131.8, 131.5, 127.7, 117.2, 110.4, 79.3, 69.2, 67.7, 58.7, 38.4, 25.4, 21.5. HRMS (ESI) *m/z* calculated for C_24_H_30_NO_7_S^+^, (M+H)^+^ 476.1737; found 476.1741.

**1r 1-(2,6-dimethylphenyl)azetidine:** Synthesis of **1r** was conducted on 4.3 mmol scale according to *General Procedure A* to give **1r** as a pale yellow oil (0.43 g, 62%). ^1^H NMR (CDCl_3_, 500 MHz): δ 6.86 (d, *J* = 7.3 Hz, 2H), 6.66 (t, *J* = 7.3 Hz, 1H), 4.18 – 4.12 (m, 4H), 2.30 (s, 6H), 2.21 – 2.15 (m, 2H). ^13^C NMR (CDCl_3_, 125 MHz): δ 150.6, 130.2, 125.2, 119.5, 57.2, 21.2, 17.6. HRMS (ESI) *m/z* calculated for C_11_H_16_N^+^ [M+H]^+^ 162.1277; found: 162.1277.

**1-Boc tert-butyl azetidine-1-carboxylate:** Synthesis of **1-Boc** was conducted on 10 mmol scale from azetidine according to a reported literature procedure^15^ to give **1-Boc** as a clear oil (1.6 g, quant.). Product characterization matched reported literature values.^15^

**1-Bn 1-benzylazetidine:** Synthesis of **1-Bn** was conducted on 10 mmol scale according to a reported literature procedure^16^ to give **1-Bn** a clear liquid (0.76 g, 52% over two steps). Product characterization matched reported literature values.^17^

**1-Ts 1-tosylazetidine:** Synthesis of **1-Ts** was conducted on 5 mmol scale according to a reported literature procedure^18^ to give **1-Ts** as a white solid (0.25 g, 24%). Product characterization matched reported literature values.^18^

**1-Bz azetidin-1-yl(phenyl)methanone:** Synthesis of **1-Bz** was conducted on 5 mmol scale according to a reported literature procedure^19^ to give **1-Bz** as a clear oil (0.24 g, 30%). Product characterization matched reported literature values.^19^

# III. Preparation and Characterization of Diazo Starting Materials

**Figure S1-2.** Diazoesters and related carbene precursors prepared in these studies.

Known compounds **2a-2g**,^20^ **2h**,^21^ **2i**,^22^ **2j**,^23^ **2k**,^24^ **2l**,^25^ **2n**,^26^ and **2o**^27^ were prepared according to published literature procedures. Product characterization matched the spectral data previously reported. Compound **2m** was prepared according to a reported literature procedure.^28^ Due to observed product volatility, the product was used as an 88% w/w solution in diethyl ether determined through ^1^H NMR.

# IV. Preparation and Characterization of Pyrrolidine Products

*General Procedure C – Carbene Transfer:* A flame-dried 2-dram vial with stirbar and septum was evacuated and refilled with nitrogen, then charged with Rh_2_(esp)_2_ (2 mol %). The azetidine (0.1 mmol) was then added and stirring was begun. The solid azetidines were added by briefly removing the septum, followed by the addition of 1 mL of anhydrous diethyl ether. Oil or liquid azetidines were added as a solution in 1 mL of anhydrous diethyl ether. A solution of the diazo compound (0.2 mmol) in anhydrous diethyl ether (0.1 M) was then added via syringe. The reaction was stirred under positive nitrogen pressure for two hours at room temperature. The crude reaction was then either concentrated under reduced pressure or worked up following *General Procedure D – Silica Catch Workup* (see below). After concentration of the crude reaction, mesitylene was added as internal standard for determination of NMR yields and diastereomeric ratio. The crude residues were then purified through column chromatography.

*Note:* upon addition of the azetidine solution, the reaction often turns a purple color as the catalyst binds to the azetidine. Addition of the diazo compound typically alters the reaction to a color similar to that of the diazo compound. As the reaction progresses, the reaction color often changes, usually with accompanying bubbling signifying the extrusion of N_2_, and typically ends as a light green color. Occasionally, reactions were found to require additional time or equiv of diazo to reach full conversion, or were run in 1,4-dioxane to improve the reaction profile for purification; these cases are noted as they occur in the SI.

*General Procedure D – Silica Catch Workup:* Many of the pyrrolidine products described were found to coelute with the C-H insertion byproduct resulting from excess diazo inserting into diethyl ether, resulting in inseparable mixtures or low isolated yields. Wherever possible, purification of pyrrolidine products was simplified by the use of a ‘silica catch’ workup adapted from Aggarwal and coworkers.^29^

Upon completion of the reaction, TsOH•H_2_O (3 equiv) was added to the reaction vial. Often, formation of a cloudy white or brown oily precipitate was observed. After stirring for 30 min, 3 mL of pentane was added to aid in further precipitation. A Büchner funnel was loaded with 10 mL silica gel and wet with hexanes (**Figure S1-3-A**). The crude reaction mixture was gently pipetted on top of the silica and the aspirator opened. The vial was rinsed with ethyl acetate to complete the addition of the crude onto the silica. The silica was then washed with 10-15 mL of ethyl acetate. Often, a blue band eluted first, while the ‘caught’ product remained on top of the silica (**Figure S1-3-B**, green layer). The silica was allowed to dry on the funnel and then was transferred to a flask with 15 mL of diethyl ether (**Figure S1-3-C**). Triethylamine (4 equiv) was added to this flask and gently swirled to deprotonate the ‘caught’ pyrrolidine product, and a color change was typically observed (colors varied, but often from an orange or green to pale yellow or white, **Figure S1-3-D and E**). The silica was filtered and the filtrate concentrated to give the nearly-clean pyrrolidine product (**Figure S1-3-F**).

**Figure S1-3**. Pictorial description of a ‘silica catch’ purification of a pyrrolidine product.

**3aa methyl 1,2-diphenylpyrrolidine-2-carboxylate:** Following *General Procedure C*, synthesis of **3aa** was conducted on 0.1 mmol scale. The product was purified by silica gel column chromatography (0% to 10% diethyl ether:hexanes) to give **3aa** as an off-white solid (25.7 mg, 91%). ^1^H NMR (CDCl_3_, 500 MHz): δ 7.39 (d, *J* = 7.4 Hz, 2H), 7.26 (t, *J* = 7.3 Hz, 2H), 7.22 – 7.18 (m, 1H), 7.07 (t, *J* = 8.5, 7.7 Hz, 2H), 6.63 (t, *J* = 7.2 Hz, 1H), 6.41 (d, *J* = 8.0 Hz, 2H), 3.73 – 3.67 (m, 1H), 3.66 – 3.58 (m, 1H), 3.55 (s, 3H), 2.73 (ddd, *J* = 12.0, 10.6, 6.7 Hz, 1H), 2.26 (ddd, *J* = 11.9, 6.7, 3.5 Hz, 1H), 1.99 (dtt, *J* = 13.0, 6.5, 3.1 Hz, 1H), 1.90 – 1.77 (m, 1H). ^13^C NMR (CDCl_3_, 125 MHz): δ 175.0, 146.1, 140.5, 128.7, 128.0, 127.7, 127.0, 116.6, 113.3, 73.7, 52.4, 50.1, 44.8, 23.0. HRMS (ESI) *m/z* calculated for C_18_H_20_NO_2_^+^, (M+H)^+^ 282.1489; found 282.1484.

**3ba methyl 1-(4-methoxyphenyl)-2-phenylpyrrolidine-2-carboxylate:** Following *General Procedure C*, synthesis of **3ba** was conducted on 0.1 mmol scale. The reaction was worked up according to *General Procedure D*, followed by silica gel column chromatography (0% to 10% diethyl ether:hexanes) to give **3ba** as a white solid (26.2 mg, 84%). ^1^H NMR (CDCl_3_, 500 MHz): δ 7.43 – 7.36 (m, 2H), 7.29 – 7.17 (m, 3H), 6.70 – 6.63 (m, 2H), 6.39 – 6.32 (m, 2H), 3.66 (s, 4H), 3.58 (td, *J* = 8.8, 6.5 Hz, 1H), 3.54 (s, 3H), 3.52 (s, 0H), 2.74 (ddd, *J* = 11.9, 10.4, 6.7 Hz, 1H), 2.23 (ddd, *J* = 11.8, 6.8, 3.6 Hz, 1H), 2.03 – 1.93 (m, 1H), 1.90 – 1.77 (m, 1H). ^13^C NMR (CDCl_3_, 125 MHz): δ 175.1, 151.2, 141.0, 140.6, 128.0, 127.7, 126.9, 114.3, 114.1, 73.9, 55.6, 52.3, 50.5, 44.9, 23.1. HRMS (ESI) *m/z* calculated for C_19_H_22_NO_3_^+^, (M+H)^+^ 312.1594; found 312.1592.

Synthesis of **3ba** was additionally performed on 1.0 mmol scale according to the following procedure. A flame-dried 100 mL round bottom flask with stirbar and septum was evacuated and refilled with nitrogen, then charged with Rh_2_(esp)_2_ (0.2 mol %, 1.5 mg). The solid azetidine (1.0 mmol) was then added, followed by 10 mL anhydrous diethyl ether, and stirring was begun. A solution of the diazo compound (2.0 mmol) in anhydrous diethyl ether (20 mL) was then added via syringe in one portion. The reaction was stirred under positive nitrogen pressure at room temperature for 24 hours. The crude reaction was then concentrated under reduced pressure and purified through silica gel column chromatography (0% to 20% diethyl ether:petroleum ether) to give **3ba** as a white solid (300 mg, 95%). Characterization matched that reported for the 0.1 mmol scale reaction above.

**3bb methyl 2-(4-fluorophenyl)-1-(4-methoxyphenyl)pyrrolidine-2-carboxylate:** Following *General Procedure C*, synthesis of **3bb** was conducted on 0.1 mmol scale. The reaction was worked up according to *General Procedure D*, followed by silica gel column chromatography (0% to 10% diethyl ether:hexanes) to give **3bb** as a white solid (31.9 mg, 97%). ^1^H NMR (CDCl_3_, 500 MHz): δ 7.45 – 7.37 (m, 2H), 6.98 (t, *J* = 8.7 Hz, 2H), 6.74 – 6.68 (m, 2H), 6.40 – 6.33 (m, 2H), 3.73 – 3.65 (m, 4H), 3.60 (td, *J* = 8.9, 8.8, 6.4 Hz, 1H), 3.56 (s, 3H), 2.74 (td, *J* = 11.3, 6.7 Hz, 1H), 2.22 (ddd, *J* = 11.8, 6.7, 3.3 Hz, 1H), 2.03 (dtt, *J* = 12.7, 6.4, 2.8 Hz, 1H), 1.92 – 1.81 (m, 1H). ^13^C NMR (CDCl_3_, 125 MHz): δ 175.1, 161.8 (d, *J* = 245.6 Hz), 151.4, 140.5, 136.6 (d, *J* = 3.3 Hz), 129.7 (d, *J* = 8.0 Hz), 114.4 (d, *J* = 21.3 Hz), 114.4, 114.0, 73.4, 55.6, 52.4, 50.5, 44.8, 23.0. ^19^F NMR (CDCl_3_, 377 MHz): δ -116.3. HRMS (ESI) *m/z* calculated for C_19_H_21_FNO_3_^+^, (M+H)^+^ 330.1500; found 330.1496.

**3bc methyl 1,2-bis(4-methoxyphenyl)pyrrolidine-2-carboxylate:** Following *General Procedure C*, synthesis of **3bc** was conducted on 0.1 mmol scale. The reaction was worked up according to *General Procedure D*, followed by silica gel column chromatography (0% to 30% diethyl ether:hexanes) to give **3bc** as an off-white solid (27.7 mg, 81%). ^1^H NMR (CDCl_3_, 500 MHz): δ 7.38 – 7.32 (m, 2H), 6.91 – 6.78 (m, 2H), 6.75 – 6.68 (m, 2H), 6.43 – 6.36 (m, 2H), 3.79 (s, 3H), 3.73 – 3.65 (m, 4H), 3.64 – 3.58 (m, 1H), 3.57 (s, 3H), 2.73 (ddd, *J* = 11.8, 10.6, 6.7 Hz, 1H), 2.23 (ddd, *J* = 11.8, 6.7, 3.5 Hz, 1H), 2.01 (ddq, *J* = 12.9, 6.5, 3.2, 3.1 Hz, 1H), 1.94 – 1.81 (m, 1H). ^13^C NMR (CDCl_3_, 125 MHz): δ 175.4, 158.5, 151.2, 140.7, 132.9, 129.1, 114.3, 114.0, 113.0, 73.4, 55.6, 55.2, 52.3, 50.4, 44.8, 23.1. HRMS (ESI) *m/z* calculated for C_20_H_24_NO_4_^+^, (M+H)^+^ 342.1700; found 342.1697.

**3bd methyl 2-(4-bromophenyl)-1-(4-methoxyphenyl)pyrrolidine-2-carboxylate:** Following *General Procedure C*, synthesis of **3bd** was conducted on 0.1 mmol scale. The reaction was worked up according to *General Procedure D*, followed by silica gel column chromatography (0% to 10% diethyl ether:hexanes) to give **3bd** as a white solid (31.3 mg, 80%). ^1^H NMR (CDCl_3_, 500 MHz): δ 7.42 (d, *J* = 8.5 Hz, 2H), 7.33 (d, *J* = 8.4 Hz, 2H), 6.71 (d, *J* = 8.9 Hz, 2H), 6.37 (d, *J* = 9.0 Hz, 2H), 3.71 (s, 3H), 3.70 – 3.67 (m, 1H), 3.63 – 3.59 (m, 1H), 3.56 (s, 3H), 2.75 (td, *J* = 11.4, 6.8 Hz, 1H), 2.25 – 2.17 (m, 1H), 2.08 – 1.98 (m, 1H), 1.92 – 1.81 (m, 1H). ^13^C NMR (CDCl_3_, 125 MHz): δ 174.8, 151.5, 140.4, 140.2, 130.8, 129.9, 121.1, 114.4, 114.0, 73.5, 55.6, 52.4, 50.5, 44.7, 23.1. HRMS (ESI) *m/z* calculated for C_19_H_21_BrNO_3_^+^, (M+H)^+^ 390.0699; found 390.0695.

**3be methyl 1-(4-methoxyphenyl)-2-(p-tolyl)pyrrolidine-2-carboxylate:** Following *General Procedure C*, synthesis of **3be** was conducted on 0.1 mmol scale. The reaction was worked up according to *General Procedure D*, followed by silica gel column chromatography (0% to 10% diethyl ether:hexanes) to give **3be** as a white solid (26.7 mg, 82%). ^1^H NMR (CDCl_3_, 500 MHz): δ 7.32 (d, *J* = 8.1 Hz, 2H), 7.11 (d, *J* = 7.9 Hz, 2H), 6.71 (d, *J* = 8.9 Hz, 2H), 6.40 (d, *J* = 8.9 Hz, 2H), 3.71 (s, 3H), 3.70 – 3.65 (m, 1H), 3.65 – 3.59 (m, 1H), 3.58 (s, 3H), 2.80 – 2.72 (m, 1H), 2.34 (s, 3H), 2.25 (ddd, *J* = 10.8, 6.8, 3.6 Hz, 1H), 2.02 (ddq, *J* = 12.7, 6.7, 3.3, 3.1 Hz, 1H), 1.94 – 1.83 (m, 1H). ^13^C NMR (CDCl_3_, 125 MHz): δ 175.3, 153.3, 151.2, 140.7, 138.0, 136.6, 128.4, 127.9, 114.3, 114.1, 73.7, 55.6, 52.3, 50.5, 44.9, 23.1, 21.0. HRMS (ESI) *m/z* calculated for C_20_H_24_NO_3_^+^, (M+H)^+^ 326.1751; found 326.1747.

**3bf methyl 1-(4-methoxyphenyl)-2-(4-(trifluoromethyl)phenyl)pyrrolidine-2-carboxylate:** Following *General Procedure C*, synthesis of **3bf** was conducted on 0.1 mmol scale, taking three hours to reach full conversion. The reaction was worked up according to *General Procedure D*, followed by silica gel column chromatography (0% to 10% diethyl ether:hexanes) to give **3bf** as a white solid (32.6 mg, 86%). ^1^H NMR (CDCl_3_, 500 MHz): δ 7.61 – 7.53 (m, 4H), 6.72 (d, *J* = 8.9 Hz, 2H), 6.37 (d, *J* = 8.9 Hz, 2H), 3.76 – 3.69 (m, 4H), 3.63 (td, *J* = 8.9, 8.7, 6.3 Hz, 1H), 3.57 (s, 3H), 2.79 (ddd, *J* = 12.1, 10.7, 6.8 Hz, 1H), 2.25 (ddd, *J* = 12.1, 6.8, 3.3 Hz, 1H), 2.10 – 2.02 (m, 1H), 1.92 – 1.82 (m, 1H). ^13^C NMR (CDCl_3_, 125 MHz): δ 174.7, 151.6, 145.3, 140.3, 129.2 (q, *J* = 31.7 Hz), 128.5, 124.6 (q, *J* = 3.8 Hz), 124.2 (q, *J* = 272.1 Hz), 114.5, 114.1, 73.7, 55.6, 52.5, 50.6, 44.7, 23.1. ^19^F NMR (CDCl_3_, 377 MHz): δ -62.4. HRMS (ESI) *m/z* calculated for C_20_H_21_F_3_NO_3_^+^, (M+H)^+^ 380.1468; found 380.1467.

**3bg methyl 2-(4-(*tert*-butyl)phenyl)-1-(4-methoxyphenyl)pyrrolidine-2-carboxylate:** Following *General Procedure C*, synthesis of **3bg** was conducted on 0.1 mmol scale. The reaction was worked up according to *General Procedure D*, followed by silica gel column chromatography (0% to 10% diethyl ether:hexanes) to give **3bg** as a white solid (31.8 mg, 87%). ^1^H NMR (CDCl_3_, 500 MHz): δ 7.31 – 7.19 (m, 4H), 6.67 – 6.60 (m, 2H), 6.36 – 6.29 (m, 2H), 3.63 (s, 3H), 3.62 – 3.58 (m, 1H), 3.54 (dd, *J* = 8.9, 6.4 Hz, 1H), 3.49 (s, 3H), 2.66 (ddd, *J* = 12.0, 10.5, 6.8 Hz, 1H), 2.19 (ddd, *J* = 11.9, 6.8, 3.5 Hz, 1H), 1.93 (dtt, *J* = 13.0, 6.5, 3.2 Hz, 1H), 1.86 – 1.74 (m, 1H), 1.23 (s, 9H). ^13^C NMR (CDCl_3_, 125 MHz): δ 175.4, 151.1, 149.6, 140.8, 137.8, 127.6, 124.6, 114.3, 114.0, 73.6, 55.6, 52.3, 50.5, 44.8, 34.4, 31.4, 23.1. HRMS (ESI) *m/z* calculated for C_23_H_30_NO_3_^+^, (M+H)^+^ 368.2220; found 368.2216.

**3bh ethyl 1-(4-methoxyphenyl)-2-(thiophen-3-yl)pyrrolidine-2-carboxylate:** Following *General Procedure C*, synthesis of **3bh** was conducted on 0.1 mmol scale. The reaction was worked up according to *General Procedure D*, followed by silica gel column chromatography (0% to 10% diethyl ether:hexanes) to give **3bh** as a tan solid (28.7 mg, 87%). ^1^H NMR (CDCl_3_, 500 MHz): δ 7.30 (dd, *J* = 3.1, 1.3 Hz, 1H), 7.24 (dd, *J* = 5.0, 3.0 Hz, 1H), 7.17 (dd, *J* = 5.0, 1.3 Hz, 1H), 6.75 – 6.68 (m, 2H), 6.46 – 6.39 (m, 2H), 4.16 – 4.01 (m, 2H), 3.74 – 3.66 (m, 4H), 3.57 (td, *J* = 8.6, 8.5, 6.6 Hz, 1H), 2.61 (ddd, *J* = 12.1, 9.8, 7.0 Hz, 1H), 2.25 (ddd, *J* = 11.7, 7.0, 4.1 Hz, 1H), 2.10 – 1.92 (m, 2H), 1.03 (t, *J* = 7.0 Hz, 3H). ^13^C NMR (CDCl_3_, 125 MHz): δ 174.5, 151.3, 143.1, 140.7, 127.9, 124.8, 122.8, 114.3, 114.2, 114.2, 71.4, 61.2, 55.7, 55.6, 50.4, 43.9, 23.1, 14.0. HRMS (ESI) *m/z* calculated for C_18_H_22_NO_3_S^+^, (M+H)^+^ 332.1315; found 332.1313.

**3bi methyl 1-(4-methoxyphenyl)-2-(1-tosyl-1H-indol-3-yl)pyrrolidine-2-carboxylate:** Following *General Procedure C*, synthesis of **3bi** was conducted on 0.1 mmol scale. The reaction was worked up according to *General Procedure D*, followed by silica gel column chromatography (0% to 30% ethyl acetate:hexanes) to give **3bi** as a tan solid (42.3 mg, 84%). ^1^H NMR (CDCl_3_, 500 MHz): δ 8.05 (d, *J* = 8.3 Hz, 1H), 7.74 (d, *J* = 8.3 Hz, 2H), 7.64 (s, 1H), 7.52 (d, *J* = 7.9 Hz, 1H), 7.37 – 7.30 (m, 1H), 7.30 – 7.22 (m, 3H), 6.76 – 6.69 (m, 2H), 6.58 – 6.51 (m, 2H), 3.85 – 3.76 (m, 4H), 3.67 – 3.61 (m, 4H), 2.68 (ddd, *J* = 12.2, 9.4, 6.9 Hz, 1H), 2.53 (ddd, *J* = 12.0, 7.1, 4.3 Hz, 1H), 2.42 (s, 3H), 2.15 (ddt, *J* = 16.7, 7.2, 3.5 Hz, 1H), 2.08 – 1.95 (m, 1H). ^13^C NMR (CDCl_3_, 125 MHz): δ 174.7, 151.5, 144.8, 140.5, 136.0, 135.1, 129.8, 128.9, 126.9, 126.1, 124.4, 123.7, 123.1, 121.9, 114.4, 113.9, 113.7, 69.8, 55.6, 52.4, 50.2, 40.8, 23.3, 21.6. HRMS (ESI) *m/z* calculated for C_28_H_29_N_2_O_5_S^+^, (M+H)^+^ 505.1792; found 505.1792.

**3bj tert-butyl 4-(2-(ethoxycarbonyl)-1-(4-methoxyphenyl)pyrrolidin-2-yl)-3,6-dihydropyridine-1(2H)-carboxylate:** Following *General Procedure C*, synthesis of **3bj** was conducted on 0.1 mmol scale. The reaction was concentrated under reduced pressure, followed by silica gel column chromatography (0% to 30% diethyl ether:hexanes) to give **3bj** as a colorless oil (28.8 mg, 67%).^1^H NMR (CDCl_3_, 500 MHz): δ 6.76 – 6.70 (m, 2H), 6.62 – 6.54 (m, 2H), 5.72 (br s, 1H), 4.07 – 3.93 (m, 3H), 3.87 – 3.77 (m, 1H), 3.72 (s, 3H), 3.66 (br s, 1H), 3.52 – 3.42 (m, 2H), 3.20 (ddd, *J* = 12.3, 7.9, 3.8 Hz, 1H), 2.55 – 2.47 (m, 1H), 2.44 (ddd, *J* = 12.2, 9.0, 6.9 Hz, 1H), 2.16 – 2.07 (m, 1H), 2.03 – 1.85 (m, 3H), 1.46 (s, 9H), 0.99 (t, *J* = 7.0 Hz, 3H). ^13^C NMR (CDCl_3_, 125 MHz): δ 173.8, 154.9, 151.4, 140.8, 136.2, 117.7, 114.3, 114.2, 79.5, 74.9, 60.8, 55.7, 51.2, 50.6, 43.6, 40.1, 38.4, 28.5, 26.6, 22.9, 14.0. HRMS (ESI) *m/z* calculated for C_24_H_35_N_2_O_5_^+^, (M+H)^+^ 431.2540; found 431.2543.

**3bk ethyl 1-(4-methoxyphenyl)-2-methylpyrrolidine-2-carboxylate:** Following *General Procedure C*, synthesis of **3bk** was conducted on 0.1 mmol scale. The reaction was worked up according to *General Procedure D*, followed by silica gel column chromatography (0% to 10% diethyl ether:hexanes) to give **3bk** as a colorless oil (21.0 mg, 80%). ^1^H NMR (CDCl_3_, 500 MHz): δ 6.82 – 6.71 (m, 2H), 6.51 – 6.45 (m, 2H), 4.12 (qq, *J* = 10.7, 7.0 Hz, 2H), 3.73 (s, 3H), 3.46 (ddt, *J* = 32.1, 8.7, 6.4 Hz, 2H), 2.36 – 2.25 (m, 1H), 2.13 – 1.94 (m, 3H), 1.54 (s, 3H), 1.13 (t, *J* = 7.1 Hz, 3H). ^13^C NMR (CDCl_3_, 125 MHz): δ 176.7, 151.2, 140.4, 114.6, 114.4, 114.2, 66.0, 60.9, 55.8, 50.4, 41.5, 23.0, 21.8, 14.1. HRMS (ESI) *m/z* calculated for C_15_H_22_NO_3_^+^, (M+H)^+^ 264.1594; found 264.1595.

**3bl 1-(1-(4-methoxyphenyl)-2-phenylpyrrolidin-2-yl)ethan-1-one:** Following *General Procedure C*, synthesis of **3bl** was conducted on 0.1 mmol scale. The reaction was worked up according to *General Procedure D*, followed by silica gel column chromatography (0% to 20% diethyl ether:hexanes) to give **3bl** as a white solid (19.1 mg, 65%). ^1^H NMR (CDCl_3_, 500 MHz): δ 7.36 – 7.27 (m, 4H), 7.26 – 7.20 (m, 1H), 6.73 (d, *J* = 8.9 Hz, 2H), 6.34 (d, *J* = 8.9 Hz, 2H), 3.73 – 3.68 (m, 5H), 2.53 (td, *J* = 12.3, 6.7 Hz, 1H), 2.17 (dd, *J* = 12.2, 6.4 Hz, 1H), 2.11 – 2.01 (m, 1H), 1.97 (s, 3H), 1.87 (ttd, *J* = 12.3, 9.3, 6.7 Hz, 1H), 1.43 (d, *J* = 0.8 Hz, 0H). ^13^C NMR (CDCl_3_, 125 MHz): δ 209.5, 151.4, 140.1, 139.5, 130.3, 128.7, 127.4, 126.8, 114.8, 113.4, 78.2, 55.6, 50.1, 42.3, 25.3, 23.1. HRMS (ESI) *m/z* calculated for C_19_H_22_NO_2_^+^, (M+H)^+^ 296.1645; found 296.1645.

**3bm 1-(4-methoxyphenyl)-2-phenyl-2-(trifluoromethyl)pyrrolidine:** Following *General Procedure C*, synthesis of **3bm** was conducted on 0.1 mmol scale. The reaction was concentrated under reduced pressure, after which 10 μL mesitylene was added to give an NMR yield of 52%. Purification of the crude residue was attempted by silica gel column chromatography (0% to 10% diethyl ether:hexanes), but the product ultimately could not be isolated cleanly. Characterization data is based on the impure, columned product. ^1^H NMR (CDCl_3_, 500 MHz): δ 7.48 (d, *J* = 7.8 Hz, 2H), 7.40 – 7.31 (m, 3H), 6.72 – 6.63 (m, 2H), 6.56 – 6.49 (m, 2H), 3.84 – 3.78 (m, 1H), 3.71 (s, 4H), 2.77 (dt, *J* = 13.1, 7.8 Hz, 1H), 2.22 – 2.13 (m, 1H), 2.13 – 1.99 (m, 2H). ^13^C NMR (CDCl_3_, 125 MHz): δ 152.0, 140.0, 138.9, 128.7, 127.9 (q, *J* = 291.7 Hz), 127.5, 126.3 (q, *J* = 2.2 Hz), 116.6 (q, *J* = 2.0 Hz), 113.7, 72.7 (q, *J* = 26.0 Hz), 55.5, 52.1, 42.1 (q, *J* = 1.4 Hz), 22.0. ^19^F NMR (CDCl_3_, 377 MHz): δ -65.4. HRMS (ESI) *m/z* calculated for C_18_H_19_F_3_NO^+^, (M+H)^+^ 322.1413; found 322.1411.

**3bn 1-(4-methoxyphenyl)-7-oxa-1-azaspiro[4.4]nonan-6-one:** Following *General Procedure C*, synthesis of **3bn** was conducted on 0.1 mmol scale. After two hours, TLC analysis indicated incomplete conversion of azetidine **1b**, so an additional 1 equiv of diazo compound **2n** was added as a solution in 0.5 mL anhydrous Et_2_O. After an additional hour, the reaction reached full conversion (3 hours total). The reaction was worked up according to *General Procedure D*, followed by silica gel column chromatography (0% to 60% diethyl ether:hexanes) to give **3bn** as a white solid (12.6 mg, 51%). ^1^H NMR (CDCl_3_, 500 MHz): δ 6.81 (d, *J* = 8.9 Hz, 2H), 6.48 (d, *J* = 9.2 Hz, 2H), 4.53 (td, *J* = 9.4, 1.6 Hz, 1H), 4.27 (ddd, *J* = 10.8, 9.4, 7.0 Hz, 1H), 3.73 (s, 3H), 3.57 (dt, *J* = 8.6, 6.8 Hz, 1H), 3.48 (td, *J* = 8.4, 8.1, 4.3 Hz, 1H), 2.87 (dt, *J* = 12.9, 10.0 Hz, 1H), 2.39 – 2.29 (m, 1H), 2.25 – 2.14 (m, 2H), 2.08 – 1.98 (m, 1H), 1.92 (ddd, *J* = 13.1, 7.0, 1.6 Hz, 1H). ^13^C NMR (CDCl_3_, 125 MHz): δ 178.6, 152.0, 138.5, 114.9, 114.9, 66.0, 65.0, 55.8, 50.0, 38.7, 30.1, 23.0. HRMS (ESI) *m/z* calculated for C_14_H_18_NO_3_^+^, (M+H)^+^ 248.1281; found 248.1280.

**3ko 1-benzyl-4',4'-difluoro-1'-(4-methoxyphenyl)spiro[indoline-3,2'-pyrrolidin]-2-one:** Following *General Procedure C*, synthesis of **3ko** was conducted on 0.1 mmol scale. The reaction was concentrated under reduced pressure, followed by basic alumina column chromatography (0% to 20% diethyl ether:hexanes with standard grade Brockmann I activated basic alumina from Sigma-Aldrich) to give **3ko** as a red solid (38.6 mg, 92%). ^1^H NMR (CDCl_3_, 500 MHz): δ 7.50 (d, *J* = 7.3 Hz, 1H), 7.34 – 7.20 (m, 4H), 7.17 – 7.05 (m, 3H), 6.78 (d, *J* = 7.8 Hz, 1H), 6.66 – 6.55 (m, 2H), 6.39 – 6.29 (m, 2H), 5.13 (d, *J* = 15.5 Hz, 1H), 4.62 (d, *J* = 15.4 Hz, 1H), 4.36 (dt, *J* = 20.9, 9.5 Hz, 1H), 3.94 (ddd, *J* = 14.8, 10.1, 1.8 Hz, 1H), 3.71 (s, 3H), 3.08 (ddd, *J* = 23.6, 14.0, 12.0 Hz, 1H), 2.70 (ddt, *J* = 17.8, 14.0, 1.9 Hz, 1H). ^13^C NMR (CDCl_3_, 125 MHz): δ 176.9, 154.0, 141.8, 138.2, 135.2, 130.4, 129.6, 128.7, 127.8, 127.6, 126.2 (dd, *J* = 248.4 Hz, 245.7 Hz), 124.4 (d, *J* = 3.2 Hz), 123.6, 117.4, 114.5, 109.8, 69.0 (d, *J* = 6.2 Hz), 57.5 (dd, *J* = 31.8 Hz, 27.9 Hz), 55.4, 46.9 (dd, *J* = 26.4, 23.4 Hz), 44.0. ^19^F NMR (CDCl_3_, 377 MHz): δ -92.8 (d, *J* = 229.8 Hz), -103.7 (d, *J* = 229.8 Hz). HRMS (ESI) *m/z* calculated for C_25_H_23_F_2_N_2_O_2_^+^, (M+H)^+^ 421.1722; found 421.1724.

**3ca methyl 2-phenyl-1-(p-tolyl)pyrrolidine-2-carboxylate:** Following *General Procedure C*, synthesis of **3ca** was conducted on 0.1 mmol scale. The reaction was worked up according to *General Procedure D* and was sufficiently pure without further purification to yield **3ca** as a white solid (24.0 mg, 81%). ^1^H NMR (CDCl_3_, 500 MHz): δ 7.38 – 7.31 (m, 2H), 7.28 – 7.18 (m, 3H), 6.86 – 6.79 (m, 2H), 6.30 – 6.25 (m, 2H), 3.65 – 3.53 (m, 2H), 3.51 (s, 3H), 2.68 (ddd, *J* = 12.0, 10.6, 6.7 Hz, 1H), 2.23 – 2.18 (m, 1H), 2.12 (s, 3H), 2.01 – 1.86 (m, 1H), 1.87 – 1.69 (m, 1H). ^13^C NMR (CDCl_3_, 125 MHz): δ 175.1, 143.9, 140.7, 129.2, 128.0, 127.6, 126.9, 125.5, 113.2, 73.6, 52.4, 50.2, 44.9, 23.0, 20.3, 20.2. HRMS (ESI) *m/z* calculated for C_19_H_22_NO_2_^+^, (M+H)^+^ 296.1645; found 296.1640.

**3da methyl 2-phenyl-1-(4-(trifluoromethyl)phenyl)pyrrolidine-2-carboxylate:** Following *General Procedure C*, synthesis of **3da** was conducted on 0.1 mmol scale. The reaction was concentrated under reduced pressure, followed by silica gel column chromatography (0% to 20% diethyl ether:pentane) to give **3da** as a white solid (28.3 mg, 81%). ^1^H NMR (CDCl_3_, 400 MHz): δ 7.35 – 7.21 (m, 7H), 6.40 (d, *J* = 8.5 Hz, 2H), 3.71 (td, *J* = 8.3, 7.9, 3.3 Hz, 1H), 3.68 – 3.62 (m, 1H), 3.59 (s, 3H), 2.74 (ddd, *J* = 12.1, 10.2, 6.6 Hz, 1H), 2.28 (ddd, *J* = 12.0, 6.6, 4.1 Hz, 1H), 2.06 – 1.95 (m, 1H), 1.91 – 1.79 (m, 1H). ^13^C NMR (CDCl_3_, 125 MHz): δ 174.2, 148.4, 139.4, 128.0, 127.7, 127.3, 125.9 (q, *J* = 3.8 Hz), 125.0 (q, *J* = 270.3 Hz), 118.2 (q, *J* = 32.5 Hz), 112.9, 73.8, 52.6, 50.3, 44.8, 22.9. ^19^F NMR (CDCl_3_, 377 MHz): δ -60.9. HRMS (ESI) *m/z* calculated for C_19_H_19_F_3_NO_2_^+^, (M+H)^+^ 350.1362; found 350.1356.

**3ea methyl 1-(4-(*tert*-butyl)phenyl)-2-phenylpyrrolidine-2-carboxylate:** Following *General Procedure C*, synthesis of **3ea** was conducted on 0.1 mmol scale. The reaction was concentrated under reduced pressure, followed by silica gel column chromatography (0% to 10% diethyl ether:hexanes) to give **3ea** as a white solid (25.2 mg, 75%). ^1^H NMR (CDCl_3_, 500 MHz): δ 7.44 (d, *J* = 7.4 Hz, 2H), 7.30 (d, *J* = 7.0 Hz, 2H), 7.25 – 7.22 (m, 1H), 7.12 (d, *J* = 8.8 Hz, 2H), 6.39 (d, *J* = 8.8 Hz, 2H), 3.72 (td, *J* = 8.0, 7.9, 2.8 Hz, 1H), 3.64 (td, *J* = 8.8, 8.7, 6.4 Hz, 1H), 3.59 (s, 3H), 2.77 (ddd, *J* = 11.8, 10.4, 6.7 Hz, 1H), 2.28 (ddd, *J* = 11.9, 6.7, 3.6 Hz, 1H), 2.02 (ddq, *J* = 12.7, 6.7, 3.3, 3.2 Hz, 1H), 1.92 – 1.80 (m, 1H), 1.24 (s, 9H). ^13^C NMR (CDCl_3_, 125 MHz): δ 175.1, 143.8, 140.9, 139.1, 128.0, 127.6, 127.1, 126.9, 112.9, 73.7, 52.3, 50.2, 44.9, 33.7, 31.5, 23.0. HRMS (ESI) *m/z* calculated for C_22_H_28_NO_2_^+^, (M+H)^+^ 338.2115; found 338.2111.

**3fa methyl 1-(4-bromophenyl)-2-phenylpyrrolidine-2-carboxylate:** Following *General Procedure C*, synthesis of **3fa** was conducted on 0.1 mmol scale; the reaction was conducted using 1,4-dioxane as solvent instead of diethyl ether to minimize formation of the inseparable solvent C-H insertion byproduct. The reaction was concentrated under reduced pressure, followed by silica gel column chromatography (0% to 10% diethyl ether:hexanes) to give **3fa** as a white solid (29.4 mg, 82%). ^1^H NMR (CDCl_3_, 500 MHz): δ 7.36 – 7.32 (m, 2H), 7.28 – 7.20 (m, 3H), 7.16 – 7.09 (m, 2H), 6.30 – 6.23 (m, 2H), 3.65 (td, *J* = 8.2, 8.0, 3.2 Hz, 1H), 3.61 – 3.54 (m, 4H), 2.73 (ddd, *J* = 11.9, 10.2, 6.6 Hz, 1H), 2.25 (ddd, *J* = 11.5, 6.6, 3.9 Hz, 1H), 2.04 – 1.94 (m, 1H), 1.90 – 1.78 (m, 1H). ^13^C NMR (CDCl_3_, 125 MHz): δ 174.5, 145.1, 139.9, 131.3, 127.8, 127.8, 127.2, 115.0, 108.7, 73.8, 52.5, 50.3, 44.9, 23.0. HRMS (ESI) *m/z* calculated for C_18_H_19_BrNO_2_^+^, (M+H)^+^ 360.0594; found 360.0588.

**3ga methyl 1-(4-fluorophenyl)-2-phenylpyrrolidine-2-carboxylate:** Following *General Procedure C*, synthesis of **3ga** was conducted on 0.1 mmol scale; the reaction was conducted using 1,4-dioxane as solvent instead of diethyl ether to minimize formation of the inseparable solvent C-H insertion byproduct. The reaction was concentrated under reduced pressure, followed by silica gel column chromatography (0% to 5% diethyl ether:hexanes) to give **3ga** as a white solid (14.6 mg, 49%). ^1^H NMR (CDCl_3_, 500 MHz): δ 7.36 (d, *J* = 7.0 Hz, 2H), 7.28 – 7.21 (m, 3H), 6.76 (t, *J* = 8.8 Hz, 2H), 6.35 – 6.27 (m, 2H), 3.65 (td, *J* = 7.9, 7.7, 2.9 Hz, 1H), 3.61 – 3.56 (m, 1H), 3.55 (s, 3H), 2.74 (ddd, *J* = 11.8, 10.3, 6.7 Hz, 1H), 2.23 (ddd, *J* = 11.5, 6.8, 3.9 Hz, 1H), 1.98 (dtt, *J* = 13.4, 6.7, 3.4 Hz, 1H), 1.91 – 1.78 (m, 1H). ^13^C NMR (CDCl_3_, 125 MHz): δ 174.8, 155.3 (d, *J* = 234.9 Hz) 142.6 (d, *J* = 1.8 Hz), 140.5, 127.9, 127.8, 127.8, 127.1, 115.0 (d, *J* = 22.1 Hz), 113.9 (d, *J* = 7.2 Hz), 74.0, 52.4, 50.6, 44.9, 23.1. ^19^F NMR (CDCl_3_, 377 MHz): δ -129.3. HRMS (ESI) *m/z* calculated for C_18_H_19_FNO_2_^+^, (M+H)^+^ 300.1394; found 300.1394.

**3ha methyl 2-phenyl-1-(o-tolyl)pyrrolidine-2-carboxylate:** Following *General Procedure C*, synthesis of **3ha** was conducted on 0.1 mmol scale. The reaction was worked up according to *General Procedure D*, followed by silica gel column chromatography (0% to 10% diethyl ether:petroleum ether) to give **3ha** as a white solid (12.9 mg, 44%). ^1^H NMR (CDCl_3_, 500 MHz): δ 7.42 (d, *J* = 6.2 Hz, 2H), 7.28 – 7.25 (m, 1H), 7.24 – 7.18 (m, 2H), 7.09 (d, *J* = 7.5 Hz, 1H), 6.91 (t, *J* = 7.6, 7.4 Hz, 1H), 6.84 (t, *J* = 7.1, 7.0 Hz, 1H), 6.78 (d, *J* = 8.0 Hz, 1H), 3.78 (td, *J* = 8.7, 5.3 Hz, 1H), 3.61 (s, 3H), 3.60 – 3.52 (m, 1H), 2.75 (ddd, *J* = 13.0, 7.6, 5.6 Hz, 1H), 2.38 – 2.32 (m, 1H), 2.32 (s, 3H), 2.12 – 1.91 (m, 2H). ^13^C NMR (CDCl_3_, 125 MHz): δ 175.0, 146.4, 141.1, 132.7, 131.3, 127.7, 127.5, 126.9, 125.7, 123.5, 122.3, 75.8, 52.8, 51.9, 41.4, 22.8, 20.7. HRMS (ESI) *m/z* calculated for C_19_H_22_NO_2_^+^, (M+H)^+^ 296.1645; found 296.1644.

**3ia methyl 1-(3,5-dimethylphenyl)-2-phenylpyrrolidine-2-carboxylate:** Following *General Procedure C*, synthesis of **3ia** was conducted on 0.1 mmol scale. The reaction was worked up according to *General Procedure D*, followed by silica gel column chromatography (0% to 20% diethyl ether:pentane) to give **3ia** as a colorless oil (25.0 mg, 81%). ^1^H NMR (CDCl_3_, 500 MHz): δ 7.43 – 7.33 (m, 2H), 7.29 – 7.18 (m, 3H), 6.31 (s, 1H), 6.05 (s, 2H), 3.72 – 3.58 (m, 2H), 3.57 (s, 3H), 2.70 (td, *J* = 11.4, 6.6 Hz, 1H), 2.27 (ddd, *J* = 11.8, 6.5, 3.2 Hz, 1H), 2.13 (s, 6H), 1.96 (s, 1H), 1.85 – 1.70 (m, 1H). ^13^C NMR (CDCl_3_, 125 MHz): δ 175.2, 146.3, 140.6, 138.2, 128.1, 127.6, 126.9, 118.8, 111.2, 73.6, 52.4, 50.1, 44.9, 29.7, 22.9, 21.7. HRMS (ESI) *m/z* calculated for C_20_H_24_NO_2_^+^, (M+H)^+^ 310.1802; found 310.1796.

**3jd methyl 2-(4-bromophenyl)-1-(pyridin-2-yl)pyrrolidine-2-carboxylate:** Following *General Procedure C*, synthesis of **3jd** was conducted on 0.1 mmol scale. The reaction was worked up according to *General Procedure D*, followed by silica gel column chromatography (0% to 20% diethyl ether:hexanes) to give **3jd** as a white solid (15.4 mg, 43%). ^1^H NMR (CDCl_3_, 500 MHz): δ 8.00 (d, *J* = 4.6 Hz, 1H), 7.45 (ddd, *J* = 8.7, 7.1, 1.9 Hz, 1H), 7.38 (d, *J* = 8.6 Hz, 2H), 7.21 (d, *J* = 8.4 Hz, 2H), 6.55 (dd, *J* = 7.1, 4.9 Hz, 1H), 6.40 (d, *J* = 8.4 Hz, 1H), 3.78 – 3.65 (m, 2H), 3.58 (s, 3H), 2.66 (td, *J* = 11.9, 6.6 Hz, 1H), 2.33 (ddd, *J* = 12.0, 6.4, 2.4 Hz, 1H), 2.13 – 2.03 (m, 1H), 1.87 – 1.74 (m, 1H). ^13^C NMR (CDCl_3_, 125 MHz): δ 174.2, 155.9, 147.7, 139.6, 137.1, 130.5, 129.9, 121.0, 112.6, 106.5, 72.1, 52.4, 48.4, 42.9, 23.3. HRMS (ESI) *m/z* calculated for C_17_H_18_BrN_2_O_2_^+^, (M+H)^+^ 361.0546; found 361.0546.

**3ka methyl 4,4-difluoro-1-(4-methoxyphenyl)-2-phenylpyrrolidine-2-carboxylate:** Following *General Procedure C*, synthesis of **3ka** was conducted on 0.1 mmol scale. The reaction was concentrated under reduced pressure, followed by basic alumina column chromatography (0% to 20% diethyl ether:hexanes with standard grade Brockmann I activated basic alumina from Sigma-Aldrich) to give **3ka** as a white solid (25.9 mg, 75%). ^1^H NMR (CDCl_3_, 500 MHz): δ 7.46 (d, *J* = 6.8 Hz, 2H), 7.36 – 7.26 (m, 3H), 6.69 (d, *J* = 9.0 Hz, 2H), 6.40 (d, *J* = 8.9 Hz, 2H), 4.08 (ddd, *J* = 14.6, 10.4, 6.1 Hz, 1H), 3.96 (dt, *J* = 17.3, 11.2 Hz, 1H), 3.70 (s, 3H), 3.64 (s, 3H), 3.33 (dt, *J* = 17.1, 13.3 Hz, 1H), 2.70 (td, *J* = 14.3, 8.9 Hz, 1H). ^13^C NMR (CDCl_3_, 125 MHz): δ 172.8, 152.6, 139.6, 139.0, 128.3, 127.7, 127.3, 125.6 (t, *J* = 246.4 Hz), 115.6, 114.2, 73.2 (dd, *J* = 4.3, 1.9 Hz), 57.4 (dd, *J* = 31.9, 29.2 Hz), 55.5, 52.6, 51.3 (dd, *J* = 24.2, 22.9 Hz). ^19^F NMR (CDCl_3_, 377 MHz): δ -95.4 (d, *J* = 231.8 Hz), -101.4 (d, *J* = 231.9 Hz). HRMS (ESI) *m/z* calculated for C_19_H_20_F_2_NO_3_^+^, (M+H)^+^ 348.1406; found 348.1402.

**3la methyl 1-(4-methoxyphenyl)-4-((methylsulfonyl)oxy)-2-phenylpyrrolidine-2-carboxylate:** Following *General Procedure C*, synthesis of **3la** was conducted on 0.1 mmol scale. The reaction was concentrated under reduced pressure, followed by silica gel column chromatography (0% to 40% ethyl acetate:hexanes) to give **3la** as a white solid (33.0 mg, 81%, >20:1 *d.r.*). ^1^H NMR (CDCl_3_, 500 MHz): δ 7.44 – 7.39 (m, 2H), 7.28 – 7.23 (m, 2H), 7.21 – 7.18 (m, 1H), 6.70 – 6.63 (m, 2H), 6.44 – 6.37 (m, 2H), 5.39 (tt, *J* = 5.1, 4.0, 1.6 Hz, 1H), 3.94 (dt, *J* = 11.0, 1.6 Hz, 1H), 3.88 (dd, *J* = 10.9, 4.5 Hz, 1H), 3.65 (s, 3H), 3.49 (s, 3H), 3.08 (dd, *J* = 14.2, 5.1 Hz, 1H), 2.64 (dt, *J* = 14.2, 2.0 Hz, 1H), 2.47 (s, 3H). ^13^C NMR (CDCl_3_, 125 MHz): δ 173.8, 152.3, 140.5, 139.6, 128.0, 127.8, 127.3, 114.9, 114.4, 78.0, 73.3, 56.4, 55.6, 52.6, 49.7, 38.4. HRMS (ESI) *m/z* calculated for C_20_H_24_NO_6_S^+^, (M+H)^+^ 406.1319; found 406.1319.

**3ma Dimethyl 1-(4-methoxyphenyl)-2-phenylpyrrolidine-2,4-dicarboxylate:** Following *General Procedure C*, synthesis of **3ma** was conducted on 0.1 mmol scale. The reaction was worked up according to *General Procedure D*, followed by silica gel column chromatography (0% to 20% diethyl ether:hexanes) to give **3ma** as a tan solid (31.6 mg, 86%, >20:1 *d.r.*). ^1^H NMR (CDCl_3_, 500 MHz): δ 7.55 (d, *J* = 7.5 Hz, 2H), 7.45 (t, *J* = 7.4 Hz, 2H), 7.42 – 7.37 (m, 1H), 6.87 (d, *J* = 9.0 Hz, 2H), 6.61 (d, *J* = 8.8 Hz, 2H), 4.31 (dd, *J* = 8.9, 5.7 Hz, 1H), 3.99 (t, *J* = 8.4 Hz, 1H), 3.88 (s, 3H), 3.83 (s, 3H), 3.69 (s, 3H), 3.53 (qd, *J* = 7.7, 5.6 Hz, 1H), 3.20 (dd, *J* = 12.8, 7.5 Hz, 1H), 2.76 (dd, *J* = 12.8, 7.9 Hz, 1H). ^13^C NMR (CDCl_3_, 125 MHz): δ 174.1, 173.0, 152.0, 151.6, 140.1, 139.9, 127.9, 127.4, 127.2, 117.0, 115.0, 114.1, 73.7, 55.6, 52.7, 52.4, 52.0, 46.7, 41.1. HRMS (ESI) *m/z* calculated for C_21_H_24_NO_5_^+^, (M+H)^+^ 370.1649; found 370.1647.

**3na Methyl 3-cyano-1-(4-methoxyphenyl)-2-phenylpyrrolidine-2-carboxylate:** Following *General Procedure C*, synthesis of **3na** was conducted on 0.1 mmol scale. The reaction was concentrated under reduced pressure, followed by silica gel column chromatography (0% to 30% diethyl ether:hexanes) to give diastereomers **3na** and **3na’** as white solids (total yield 21.1 mg, 63%, >20:1 *r.r.*, 1.2:1 *d.r.*; **3na** 11.7 mg, 35%; **3na’** 9.4 mg, 28%). ^1^H NMR (CDCl_3_, 500 MHz): δ 7.49 – 7.43 (m, 2H), 7.31 – 7.25 (m, 3H), 6.69 – 6.60 (m, 2H), 6.41 – 6.34 (m, 2H), 3.88 (dt, *J* = 9.2, 7.3 Hz, 1H), 3.75 (td, *J* = 8.9, 4.1 Hz, 1H), 3.70 (s, 3H), 3.64 (s, 3H), 3.19 (dd, *J* = 9.7, 7.9 Hz, 1H), 2.58 (dtd, *J* = 12.3, 9.3, 6.9 Hz, 1H), 2.36 (dtd, *J* = 12.0, 7.7, 4.1 Hz, 1H). ^13^C NMR (CDCl_3_, 125 MHz): δ 170.8, 152.1, 139.2, 138.1, 128.4, 128.1, 127.1, 118.2, 115.5, 114.2, 74.8, 55.5, 53.0, 52.8, 50.0, 45.5, 30.3, 27.4. HRMS (ESI) *m/z* calculated for C_20_H_21_N_2_O_3_^+^, (M+H)^+^ 337.1547; found 337.1546.

**3na’ methyl 3-cyano-1-(4-methoxyphenyl)-2-phenylpyrrolidine-2-carboxylate:** ^1^H NMR (CDCl_3_, 500 MHz): δ 7.57 – 7.46 (m, 2H), 7.43 – 7.32 (m, 3H), 6.74 – 6.64 (m, 2H), 6.51 – 6.39 (m, 2H), 4.04 (dd, *J* = 9.4, 6.5 Hz, 1H), 3.84 (td, *J* = 8.3, 8.0, 3.4 Hz, 1H), 3.74 – 3.68 (m, 4H), 3.64 (s, 3H), 2.45 (dtd, *J* = 12.6, 6.5, 3.3 Hz, 1H), 2.31 – 2.21 (m, 1H). ^13^C NMR (CDCl_3_, 125 MHz): δ 171.9, 152.3, 138.7, 134.9, 128.7, 128.0, 117.7, 115.7, 114.1, 75.0, 55.5, 53.0, 49.2, 43.2, 30.3, 29.7, 27.7.

**3oa methyl 1,2-diphenyl-3-((triisopropylsilyl)ethynyl)pyrrolidine-2-carboxylate:** Following *General Procedure C*, synthesis of **3oa** was conducted on 0.1 mmol scale. The reaction was concentrated under reduced pressure, followed by silica gel column chromatography (0% to 5% diethyl ether:petroleum ether) to give a mixture of diastereomers of **3oa** as a white solid (36.1 mg, 78%, >20:1 *r.r.*, 1.4:1 *d.r.*). ^1^H NMR (CDCl_3_, 500 MHz): δ 7.60 – 7.54 (m, 2H), 7.50 – 7.44 (m, 2H), 7.23 – 7.12 (m, 7H), 7.06 – 6.97 (m, 4H), 6.59 (tdt, *J* = 7.1, 4.9, 1.0 Hz, 2H), 6.37 (dd, *J* = 8.9, 1.0 Hz, 2H), 6.33 (dd, *J* = 8.8, 1.0 Hz, 1H), 3.83 – 3.76 (m, 2H), 3.72 (td, *J* = 9.2, 2.2 Hz, 1H), 3.67 – 3.62 (m, 2H), 3.58 (s, 4H), 3.53 (s, 3H), 3.13 (dd, *J* = 11.6, 7.4 Hz, 1H), 2.47 (tt, *J* = 11.8, 9.2 Hz, 1H), 2.30 – 2.20 (m, 2H), 1.97 – 1.86 (m, 1H), 1.05 – 1.00 (m, 25H), 0.92 – 0.83 (m, 19H). ^13^C NMR (CDCl_3_, 125 MHz): δ 173.4, 172.0, 145.9, 145.4, 139.6, 135.2, 129.5, 128.6, 127.6, 127.5, 127.3, 127.3, 127.1, 127.0, 116.9, 116.9, 114.0, 113.4, 104.6, 104.4, 87.8, 85.7, 75.0, 74.9, 52.5, 52.1, 50.0, 48.6, 48.4, 45.4, 33.6, 30.6, 30.3, 18.5, 18.4, 11.2, 11.0. HRMS (ESI) *m/z* calculated for C_29_H_40_NO_2_Si^+^, (M+H)^+^ 462.2823; found 462.2819.

Synthesis of **3oa** was additionally performed on 1.0 mmol scale according to the following procedure. A flame-dried 100 mL round bottom flask with stirbar and septum was evacuated and refilled with nitrogen, then charged with Rh_2_(esp)_2_ (0.2 mol %, 1.5 mg). The solid azetidine (1.0 mmol) was then added, followed by 10 mL anhydrous diethyl ether, and stirring was begun. A solution of the diazo compound (2.0 mmol) in anhydrous diethyl ether (20 mL) was then added via syringe in one portion. The reaction was stirred under positive nitrogen pressure at room temperature for 5 hours. The crude reaction was then concentrated under reduced pressure and purified through silica gel column chromatography (0% to 5% diethyl ether:petroleum ether) to give a mixture of diastereomers of **3oa** as a pale white-yellow solid (370 mg, 80%, >20:1 *r.r.*, 1.3:1 *d.r.*). Characterization matched that reported for the 0.1 mmol scale reaction above.

**3pa methyl 4-(4-(8-chloro-5,6-dihydro-11H-benzo[5,6]cyclohepta[1,2-b]pyridin-11-ylidene)piperidin-1-yl)-1-(4-methoxyphenyl)-2-phenylpyrrolidine-2-carboxylate:** Following *General Procedure C*, synthesis of **3pa** was conducted on 0.1 mmol scale. After eight hours, the reaction had not reached full conversion of **1p** as monitored by TLC, at which point an additional equiv of diazo **2a** was added as a solution in 1 mL anhydrous Et_2_O. The reaction was left to stir overnight, for a total reaction time of 24 h, at which point the reaction had reached full conversion by TLC. The reaction was worked up according to *General Procedure D*, but using dichloromethane instead of ethyl acetate to load the protonated compound onto the silica catch, and using 2.5% methanol:dichloromethane instead of ethyl acetate to elute the deprotonated compound from the silica. The crude residue was purified by silica gel column chromatography (0% to 2.5% methanol:dichloromethane) to give an inseparable mixture of diastereomers of **3pa** as a pink solid (32.1 mg, 52%). Diastereomeric ratio could not be determined from the ^1^H NMR, but diastereomers are observable in the ^13^C spectrum. ^1^H NMR (CDCl_3_, 500 MHz): δ 8.41 (td, *J* = 5.2, 5.1, 1.6 Hz, 1H), 7.44 (dt, *J* = 7.6, 1.8 Hz, 1H), 7.39 – 7.32 (m, 2H), 7.27 – 7.19 (m, 3H), 7.16 (q, *J* = 1.2 Hz, 1H), 7.14 (d, *J* = 1.3 Hz, 1H), 7.13 (d, *J* = 1.2 Hz, 1H), 7.10 (ddd, *J* = 7.9, 4.7, 3.4 Hz, 1H), 6.69 – 6.62 (m, 2H), 6.42 – 6.35 (m, 2H), 3.77 (dd, *J* = 7.7, 2.3 Hz, 2H), 3.74 (d, *J* = 2.4 Hz, 3H), 3.69 (s, 3H), 3.47 – 3.32 (m, 2H), 3.16 – 3.04 (m, 1H), 2.95 (ddd, *J* = 12.4, 6.3, 2.6 Hz, 1H), 2.82 (dddd, *J* = 20.4, 13.1, 9.8, 3.8 Hz, 4H), 2.61 – 2.31 (m, 4H), 2.21 (ddd, *J* = 38.1, 21.2, 9.7 Hz, 3H). ^13^C NMR (CDCl_3_, 125 MHz): δ 174.2, 157.5, 151.2, 146.7, 140.5, 139.7, 139.5, 138.4, 137.7, 137.3, 133.4, 132.8, 132.7, 130.8, 128.9, 128.2, 127.1, 126.8, 126.1, 122.1, 115.3, 113.9, 73.7, 61.3, 55.6, 55.5, 53.9, 53.2, 52.3, 48.9, 31.8, 31.5, 30.8, 30.6. HRMS (ESI) *m/z* calculated for C_38_H_39_ClN_3_O_3_^+^, (M+H)^+^ 620.2674; found 620.2685.

**3qa methyl 1-(4-(4-((1-isopropoxy-2-methyl-1-oxopropan-2-yl)oxy)benzoyl)phenyl)-4-((methylsulfonyl)oxy)-2-phenylpyrrolidine-2-carboxylate:** Following *General Procedure C*, synthesis of **3qa** was conducted on 0.1 mmol scale. After six hours, the reaction had not reached full conversion of **1p** as monitored by TLC, at which point an additional equiv of diazo **2a** was added as a solution in 1 mL anhydrous Et_2_O. The reaction was left to stir overnight, for a total reaction time of 24 h, at which point the reaction had reached full conversion by TLC. The reaction was concentrated under reduced pressure, followed by silica gel column chromatography (0% to 50% ethyl acetate:hexanes) to give an inseparable mixture of diastereomers of **3pa** as a white solid (51.1 mg, 82%, 5.9:1 *d.r.*). ^1^H NMR (CDCl_3_, 500 MHz): δ 7.66 (dd, *J* = 8.7, 4.1 Hz, 5H), 7.57 (d, *J* = 8.4 Hz, 0H), 7.45 – 7.40 (m, 2H), 7.33 (t, *J* = 7.8, 6.8 Hz, 2H), 7.30 – 7.25 (m, 1H), 6.83 (d, *J* = 8.5 Hz, 3H), 6.51 (d, *J* = 8.6 Hz, 2H), 6.41 (d, *J* = 8.5 Hz, 0H), 5.48 (tt, *J* = 4.5, 1.9 Hz, 1H), 5.30 (p, *J* = 4.8, 4.7 Hz, 0H), 5.06 (hept, *J* = 6.2 Hz, 1H), 4.24 (dd, *J* = 11.1, 5.4 Hz, 0H), 4.15 – 4.04 (m, 2H), 3.97 (dd, *J* = 11.0, 3.3 Hz, 0H), 3.77 (s, 1H), 3.62 (s, 3H), 3.32 (dd, *J* = 13.6, 4.1 Hz, 0H), 3.12 (dd, *J* = 14.3, 4.7 Hz, 1H), 3.04 (s, 1H), 2.82 (dt, *J* = 14.2, 1.9 Hz, 1H), 2.61 (dd, *J* = 13.5, 4.9 Hz, 0H), 2.49 (s, 3H), 1.62 (s, 8H), 1.18 (d, *J* = 6.2 Hz, 9H). ^13^C NMR (CDCl_3_, 125 MHz): δ 194.2, 194.2, 173.3, 173.1, 172.4, 158.8, 158.7, 148.7, 148.1, 138.9, 138.4, 132.0, 131.8, 131.6, 131.5, 131.5, 131.4, 128.8, 128.1, 128.0, 127.6, 127.6, 127.0, 126.6, 117.2, 113.6, 113.0, 110.5, 79.3, 79.3, 77.2, 76.1, 73.1, 73.1, 69.2, 56.1, 55.8, 53.1, 52.9, 50.2, 49.7, 38.7, 38.3, 31.5, 29.7, 25.4, 21.5. HRMS (ESI) *m/z* calculated for C_33_H_38_NO_9_S^+^, (M+H)^+^ 624.2262; found 624.2260.

# V. Procedures for Post-Functionalization and Characterization of Products

**4ba methyl 2-phenylpyrrolidine-2-carboxylate**: Secondary pyrrolidine **4ba** was synthesized through the deprotection of PMP-protected pyrrolidine **3ba** by either ceric ammonium nitrate (CAN, from isolated **3ba** or one-pot) or TCCA. The conditions for these three reactions are given separately below. All reactions were purified according to *General Procedure E*.

*General Procedure E*: Upon complete consumption of the starting material, the deprotection reaction was transferred to a separatory funnel, where it was washed with diethyl ether (3 x 10 mL). The aqueous layer was then basified with 1 M NaOH to a pH of ~9. The basified aqueous layer was extracted with dichloromethane (3 x 10 mL). Brine was added to the aqueous layer during these extractions if layers did not form clearly. The dichloromethane extracts were then washed once with brine (10 mL), dried over MgSO_4_, filtered, and concentrated under reduced pressure. The resulting light brown oil was further dried under Schlenk-line high vacuum for ~ 1 h; any longer was found to result in reduced yields and product decomposition. Products were then massed to give an isolated yield, followed by the addition of 10 μL mesitylene and NMR analysis to determine purity and NMR yield. The aqueous workup was found to be sufficient to give purified product, giving **4ba** as a light brown oil.

Isolated pyrrolidine **3ba** (0.1 mmol) was added to a vial under N_2_, followed by a 4:1 solution of MeCN:H_2_O (0.05 M, 1.6 mL:0.4 mL). The reaction was cooled to 0 °C in an ice bath, and ceric ammonium nitrate (CAN, 2.2 equiv) was then added in a single portion. The reaction was stirred for 15 min at 0 °C. The reaction was then worked up according to *General Procedure E* to give product **4** as a light brown oil (12.7 mg, 62%). Note: mesitylene signals appear in the ^1^H and ^13^C NMR spectra because product was not stable for long enough to take another NMR spectrum. **4ba methyl 2-phenylpyrrolidine-2-carboxylate:** ^1^H NMR (CDCl_3_, 500 MHz): δ 7.54 – 7.48 (m, 2H), 7.34 – 7.28 (m, 2H), 7.28 – 7.21 (m, 1H), 3.67 (s, 3H), 3.14 – 3.03 (m, 2H), 2.74 (ddd, *J* = 12.7, 8.0, 4.8 Hz, 1H), 2.66 (br s, 1H), 2.06 (dt, *J* = 12.6, 8.4 Hz, 1H), 1.92 – 1.74 (m, 2H). ^13^C NMR (CDCl_3_, 125 MHz): δ 176.1, 142.8, 128.2, 127.3, 126.1, 72.3, 52.7, 45.8, 36.7, 24.6. HRMS (ESI) *m/z* calculated for C_12_H_16_NO_2_^+^, (M+H)^+^ 206.1176; found 206.1176.

Pyrrolidine **4ba** was synthesized according to the following procedure. Isolated pyrrolidine **3ba** (0.1 mmol) was added to a vial under N_2_, followed by MeCN (1 mL), 3 M HCl (2 equiv, 0.2 mL), and H_2_O (1 mL). Trichloroisocyanuric acid (TCCA, 1.1 equiv) was then added in a single portion, at which point rapid color changes were observed, typically ending in a yellowish color. The reaction was then stirred at room temperature for 16 h. The reaction was then worked up according to *General Procedure E* to give product **4ba** as a light brown oil (13.7 mg, 67%). Characterization matched that reported above.

Pyrrolidine **3ba** was synthesized according to *General Procedure C* at 0.1 mmol scale; upon complete conversion of starting materials, the reaction was concentrated under reduced pressure. The crude residue was redissolved in a 4:1 solution of MeCN:H_2_O (0.05 M) and cooled to 0 °C in an ice bath. Ceric ammonium nitrate (CAN, 2.2 equiv) was then added in a single portion and the reaction was stirred for 15 min at 0 °C. The reaction was then worked up according to *General Procedure E* to give product **4ba** as a light brown oil (14.7 mg, 72%). Characterization matched that reported above.

**4oa 1-methyl 3-ethynyl-1,2-diphenylpyrrolidine-2-carboxylate**: Removal of the TIPS protecting group was performed according to a modified literature procedure.^30^ A flame-dried round bottom flask with stir bar and septum was charged with pyrrolidine **3oa** (0.1 mmol), dissolved in anhydrous THF (0.2 M), and cooled to 0 °C in an ice bath. A solution of TBAF (1 M in anhydrous THF, 1 equiv) was added dropwise. After 24 hours, the reaction reached full conversion according to TLC. The crude product was purified by silica flash chromatography (0 to 5% diethyl ether/hexane) to give product **4oa** as a colorless oil (23 mg, 76%, >20:1 *d.r.*).^30^ ^1^H NMR (CDCl_3_, 500 MHz): δ 7.65 – 7.61 (m, 2H), 7.60 – 7.53 (m, 2H), 7.36 – 7.24 (m, 7H), 7.15 – 7.06 (m, 5H), 6.72 – 6.64 (m, 2H), 6.48 – 6.39 (m, 5H), 3.92 – 3.79 (m, 4H), 3.74 (dd, *J* = 8.9, 4.1 Hz, 2H), 3.69 (s, 4H), 3.62 (s, 3H), 3.19 (ddd, *J* = 10.8, 7.5, 2.5 Hz, 1H), 2.51 (ddt, *J* = 11.9, 10.9, 8.8 Hz, 1H), 2.38 – 2.27 (m, 4H), 2.14 (d, *J* = 2.5 Hz, 1H), 1.99 (tt, *J* = 11.8, 8.9 Hz, 1H). ^13^C NMR (CDCl3, 125 MHz): δ 173.3, 172.3, 145.9, 145.3, 139.6, 135.1, 129.8, 128.7, 128.7, 127.9, 127.7, 127.3, 127.3, 127.0, 117.2, 117.0, 114.1, 113.4, 81.4, 80.9, 75.2, 75.1, 74.9, 73.1, 52.7, 52.3, 49.9, 48.6, 47.2, 44.1, 30.0, 29.9. HRMS (ESI) *m/z* calculated for C_20_H_20_NO_2_^+^, (M+H)^+^ 306.1489; found 306.1487.

**4la 2-methyl 4-azido-1-(4-methoxyphenyl)-2-phenylpyrrolidine-2-carboxylate**: Synthesis of **4la** was performed according to an adapted literature procedure.^31^ To a solution of **3la** (40.5 mg, 0.1 mmol) in N,N-dimethylformamide (2 mL) was added sodium azide (19.5 mg, 0.3 mmol) at room temperature and stirred for 12 h at 70 °C in a bead or sand bath. The reaction mixture was quenched with water and extracted three times with ethyl acetate. The combined organic layers were washed with brine, dried over anhydrous sodium sulfate, filtered, and concentrated in vacuo. The residue was purified by silica flash chromatography (ethyl acetate/hexane, 0−20%) to give **4la** as a white solid (25.8 mg, 73%, >20:1 *d.r.*). ^1^H NMR (CDCl_3_, 500 MHz): δ 7.28 – 7.24 (m, 2H), 7.21 – 7.11 (m, 3H), 6.61 – 6.50 (m, 2H), 6.29 – 6.22 (m, 2H), 4.02 (p, *J* = 7.3, 7.2, 7.0, 6.4 Hz, 1H), 3.82 (dd, *J* = 8.7, 6.5 Hz, 1H), 3.57 (s, 3H), 3.49 (s, 3H), 3.43 (t, *J* = 8.0, 7.6 Hz, 1H), 2.79 (dd, *J* = 12.2, 8.4 Hz, 1H), 2.41 (dd, *J* = 12.2, 6.2 Hz, 1H). ^13^C NMR (CDCl_3_, 125 MHz): δ 173.6, 151.9, 140.1, 139.5, 128.1, 127.5, 127.5, 114.6, 114.5, 114.3, 73.4, 57.1, 55.6, 54.4, 52.6, 48.9. HRMS (ESI) *m/z* calculated for C_19_H_21_N_4_O_3_^+^, (M+H)^+^ 353.1608; found 353.1610.

**4bd (2-(4-bromophenyl)-1-(4-methoxyphenyl)pyrrolidin-2-yl)methanol**: The reduction of **3bd** to product **4bd** was performed according to an adapted literature procedure.^4^ Pyrrolidine **3bd** was added to a flame-dried vial with stir bar under N_2_. Anhydrous THF (0.1 M) was added, and the reaction was cooled to 0 °C in an ice bath. LiAlH_4_ (2 equiv) was then added in a single portion to the reaction, which was stirred at 0 °C for 30 min. Upon complete conversion of the starting material, the reaction was carefully quenched through the dropwise addition of H_2_O. The reaction was then diluted with ethyl acetate, and Na_2_SO_4_ was added until the reaction was dry. The crude solution was then filtered through a silica plug, eluting with ethyl acetate, and concentrated under reduced pressure. The reaction was purified through silica gel column chromatography (0 to 30% ethyl acetate:hexanes) to give reduced product **4bd** as a white solid (30.5 mg, 84%). ^1^H NMR (CDCl_3_, 500 MHz): δ 7.43 (d, *J* = 8.3 Hz, 2H), 7.10 (d, *J* = 8.2 Hz, 2H), 6.75 – 6.69 (m, 2H), 6.47 (d, *J* = 8.7 Hz, 2H), 4.40 (d, *J* = 11.5 Hz, 1H), 4.05 (t, *J* = 10.7 Hz, 1H), 3.71 (s, 3H), 3.68 – 3.53 (m, 2H), 2.72 – 2.61 (m, 1H), 1.99 – 1.82 (m, 3H), 1.37 (d, *J* = 9.4 Hz, 1H). ^13^C NMR (CDCl_3_, 125 MHz): δ 151.7, 144.2, 139.3, 131.6, 127.7, 120.6, 114.9, 114.7, 69.9, 63.3, 55.7, 52.0, 40.9, 21.9. HRMS (ESI) *m/z* calculated for C_18_H_21_BrNO_2_^+^, (M+H)^+^ 362.0750; found 362.0747.

# VI. Chirality Transfer Studies

Enantioenriched azetidine *ent*-**1n** was synthesized from commercially available (*S*)-azetidine-2-carboxylic acid according to the following route.

**S1-***ent***-1n methyl (S)-azetidine-2-carboxylate hydrochloride:** Synthesis of **S1-***ent***-1n** was conducted on 10 mmol scale from azetidine-2-carboxylic acid according to reported literature procedure^9^ to give **S1-***ent***-1n** as a sticky yellow solid. The crude product was used without further purification, and the yield was presumed to be quantitative for the following reaction.

**S2**-*ent-***1n methyl (S)-1-(4-methoxyphenyl)azetidine-2-carboxylate:** Synthesis of **S2**-*ent-***1n** was conducted on 10 mmol scale according to *General Procedure B* to give **S2**-*ent-***1n** as a dark yellow oil (0.63 g, 29%). ^1^H NMR (CDCl_3_, 500 MHz): δ 6.84 – 6.77 (m, 2H), 6.54 – 6.48 (m, 2H), 4.40 (dd, *J* = 8.8, 7.4 Hz, 1H), 3.96 (ddd, *J* = 8.3, 6.5, 3.4 Hz, 1H), 3.81 (s, 3H), 3.74 (s, 3H), 3.67 – 3.58 (m, 1H), 2.62 (dq, *J* = 10.5, 8.0 Hz, 1H), 2.51 (dtd, *J* = 11.9, 8.7, 8.5, 3.4 Hz, 1H). ^13^C NMR (CDCl_3_, 125 MHz): δ 173.1, 153.0, 145.3, 114.6, 113.4, 63.9, 55.7, 52.3, 50.3, 21.6. HRMS (ESI) *m/z* calculated for C_12_H_16_NO_3_^+^ [M+H]^+^ 222.1125; found: 222.1125.

**S3**-*ent-***1n (S)-1-(4-methoxyphenyl)azetidine-2-carboxamide:** Synthesis of **S3**-*ent-***1n** was conducted on 2.85 mmol scale according to an adapted literature procedure^32^ to give **S3**-*ent-***1n** as a white solid (0.49 g, 83%). ^1^H NMR (CDCl_3_, 500 MHz): δ 6.91 (s, 1H), 6.87 – 6.80 (m, 2H), 6.53 – 6.46 (m, 2H), 5.39 (br s, 1H), 4.23 (t, *J* = 8.4, 8.3 Hz, 1H), 3.94 (ddd, *J* = 8.4, 6.8, 3.2 Hz, 1H), 3.76 (s, 3H), 3.67 (td, *J* = 8.5, 6.7 Hz, 1H), 2.61 (dtd, *J* = 12.1, 8.9, 3.2 Hz, 1H), 2.49 (doublet of quadruplets, *J* = 11.0, 8.2 Hz, 1H). ^13^C NMR (CDCl_3_, 125 MHz): δ 175.3, 153.4, 144.9, 114.8, 113.4, 65.3, 55.8, 50.2, 22.8, 22.6. HRMS (ESI) *m/z* calculated for C_11_H_15_N_2_O_2_^+^ [M+H]^+^ 207.1128; found: 207.1127.

*ent-***1n** **(*S*)-1-(4-methoxyphenyl)azetidine-2-carbonitrile:** Synthesis of *ent-***1n** was conducted on 1.5 mmol scale according to an adapted literature procedure.^32^
The compound was additionally purified through recrystallization from Et_2_O and hexanes in a -20 °C freezer to give *ent-***1n** as a yellow solid (40.6 mg, 14%). Characterization of *ent-***1n** matched previously reported characterization for the racemic compound.^10^

HPLC conditions for **1n** and *ent-***1n**: Shimadzu Prominence HPLC equipped with a 5 μm phase Chiralcel AD-H column. Flow rate: 0.7 mL/min.; oven temp: 40 °C. The eluent was begun at a solvent composition of 5% *i*PrOH:hexanes and held for 2 min at this composition, after which point the solvent composition was gradually increased to 80% *i*PrOH:hexanes over a period of 23 min. The eluent composition then returned to 5% *i*PrOH:hexanes over a period of 3 min and held at this composition for the final 2 min of analysis. The detector was set at 254 nm.

**Figure S1-4**. Racemic 1-(4-methoxyphenyl)azetidine-2-carbonitrile **1n** HPLC data.

**Figure S1-5.** Enantioenriched (*S*)-1-(4-methoxyphenyl)azetidine-2-carbonitrile *ent-***1n** HPLC data.

HPLC conditions for **3na** and *ent-***3na**: Shimadzu Prominence HPLC equipped with a 5 μm phase Chiralcel AD-H column. Flow rate: 0.7 mL/min.; oven temp: 40 °C. The eluent was begun at a solvent composition of 10% *i*PrOH:hexanes and held for 20 min at this composition. The detector was set at 310 nm.

**Figure S1-6**. Racemic **methyl 3-cyano-1-(4-methoxyphenyl)-2-phenylpyrrolidine-2-carboxylate** **3na** HPLC data (diastereomer 1).

**Figure S1-7**. Racemic **methyl 3-cyano-1-(4-methoxyphenyl)-2-phenylpyrrolidine-2-carboxylate** **3na’** HPLC data (diastereomer 2).

**Figure S1-8**. Enantioenriched **methyl 3-cyano-1-(4-methoxyphenyl)-2-phenylpyrrolidine-2-carboxylate** *ent-***3na** HPLC data (diastereomer 1).

**Figure S1-9**. Enantioenriched **methyl 3-cyano-1-(4-methoxyphenyl)-2-phenylpyrrolidine-2-carboxylate** *ent-***3na’** HPLC data (diastereomer 1).

# VII. Details of Rh-Azetidine Binding UV-Vis Experiments

Toluene solutions of the protected azetidine (0.03 M) and Rh_2_(esp)_2_ (1.6 mM, 6.08 mg, 0.008 mMol) were prepared the day of the study. Absorption measurements were performed on a TECAN Spark UV-Vis spectrometer in air from 400-1000 nm. Using a 96-well plate, 200.0 µL of the Rh_2_(esp)_2_ solution was added to six wells using a 250 µL micropipettor. Three wells were treated with 10.0 µL of the solution of the protected azetidine using a 10.0 µL syringe and 100.0 µL of the azetidine solution was added to the remaining three wells using a 250.0 µL micropipettor. Each well was mixed before the UV-Vis spectrum was recorded. The UV-Vis of each well was recorded simultaneously and absorbance versus wavelength data was processed using Excel.

*1:1 Adduct*

**Figure S1-10**. UV-Vis of possible 1:1 adducts with various protected azetidines (1.4 mM azetidine, 1.5 mM Rh_2_(esp)_2_). For a more detailed inspection of the data, please see **Supplementary Information 2**.

*2:1 Adduct*

UV-Vis of Rh2(esp)2 and protected azetidine 2:1 adduct

Absorbance

| Wavelenth (nm) | Rh2(esp)2 | Ph | p-methoxyphenyl | p-methylphenyl | p-trifluoromethylphenyl | p-tert-butylphenyl | p-bromophenyl | p-fluorophenyl | 3,5 dimethyl phenyl | 2,6 dimethyl phenyl | Bz | Bn | Ts | Boc |
| --- | --- | --- | --- | --- | --- | --- | --- | --- | --- | --- | --- | --- | --- | --- |
| 400 | 0.2953 | 0.2377 | 0.2564 | 0.2493 | 0.2295 | 0.2772 | 0.2612 | 0.2332 | 0.2195 | 0.2496 | 0.2673 | 0.1719 | 0.2823 | 0.2558 |
| 402 | 0.297 | 0.2319 | 0.2478 | 0.2419 | 0.2253 | 0.2705 | 0.2563 | 0.2284 | 0.2147 | 0.2407 | 0.2688 | 0.1693 | 0.2856 | 0.2585 |
| 404 | 0.2986 | 0.2286 | 0.2402 | 0.2367 | 0.2214 | 0.2639 | 0.2506 | 0.2242 | 0.2091 | 0.2317 | 0.2703 | 0.1665 | 0.2897 | 0.2622 |
| 406 | 0.2997 | 0.225 | 0.2339 | 0.2317 | 0.2182 | 0.2593 | 0.2468 | 0.2208 | 0.2049 | 0.2247 | 0.2704 | 0.1649 | 0.2922 | 0.2646 |
| 408 | 0.3002 | 0.2219 | 0.2293 | 0.2277 | 0.2163 | 0.2546 | 0.2437 | 0.2179 | 0.2019 | 0.2182 | 0.271 | 0.1636 | 0.2944 | 0.2669 |
| 410 | 0.3001 | 0.2181 | 0.224 | 0.2244 | 0.2141 | 0.2511 | 0.2408 | 0.2157 | 0.1987 | 0.2106 | 0.2718 | 0.1622 | 0.2968 | 0.2692 |
| 412 | 0.3005 | 0.2175 | 0.2211 | 0.2219 | 0.2127 | 0.2493 | 0.2392 | 0.2145 | 0.1972 | 0.2068 | 0.2716 | 0.1618 | 0.2985 | 0.2704 |
| 414 | 0.2993 | 0.2158 | 0.2185 | 0.22 | 0.2122 | 0.2474 | 0.2378 | 0.2138 | 0.1956 | 0.2015 | 0.2724 | 0.1609 | 0.299 | 0.2713 |
| 416 | 0.2986 | 0.2151 | 0.2178 | 0.2179 | 0.2112 | 0.2467 | 0.2373 | 0.2135 | 0.1949 | 0.1971 | 0.2718 | 0.1615 | 0.2991 | 0.2719 |
| 418 | 0.2973 | 0.2151 | 0.2166 | 0.2179 | 0.2123 | 0.2463 | 0.2381 | 0.2138 | 0.1949 | 0.1937 | 0.272 | 0.1619 | 0.2991 | 0.2715 |
| 420 | 0.2959 | 0.2152 | 0.2169 | 0.2178 | 0.2128 | 0.2469 | 0.2382 | 0.2151 | 0.1951 | 0.1914 | 0.2713 | 0.1629 | 0.2985 | 0.2708 |
| 422 | 0.2934 | 0.2155 | 0.2169 | 0.2175 | 0.2131 | 0.2477 | 0.2386 | 0.2158 | 0.1955 | 0.1907 | 0.2701 | 0.1636 | 0.2977 | 0.2711 |
| 424 | 0.2901 | 0.2159 | 0.2168 | 0.2178 | 0.2139 | 0.2483 | 0.24 | 0.2168 | 0.1957 | 0.1885 | 0.2693 | 0.1648 | 0.2955 | 0.2691 |
| 426 | 0.2871 | 0.2162 | 0.2174 | 0.2179 | 0.2141 | 0.2496 | 0.2406 | 0.218 | 0.1965 | 0.1872 | 0.2672 | 0.1655 | 0.2932 | 0.2681 |
| 428 | 0.2839 | 0.2168 | 0.2184 | 0.2182 | 0.2146 | 0.2499 | 0.2415 | 0.2183 | 0.1974 | 0.187 | 0.2652 | 0.167 | 0.2903 | 0.2648 |
| 430 | 0.2784 | 0.2165 | 0.2188 | 0.2192 | 0.2152 | 0.2513 | 0.2415 | 0.22 | 0.1978 | 0.1861 | 0.2623 | 0.1684 | 0.2857 | 0.2616 |
| 432 | 0.2739 | 0.2185 | 0.2189 | 0.219 | 0.2145 | 0.2517 | 0.2429 | 0.221 | 0.1983 | 0.1853 | 0.2602 | 0.1701 | 0.2811 | 0.2576 |
| 434 | 0.269 | 0.218 | 0.2194 | 0.2192 | 0.215 | 0.2524 | 0.2427 | 0.2216 | 0.1983 | 0.1852 | 0.2579 | 0.1718 | 0.2764 | 0.2538 |
| 436 | 0.2634 | 0.2184 | 0.2203 | 0.2191 | 0.2147 | 0.2524 | 0.2429 | 0.2222 | 0.1986 | 0.1854 | 0.2539 | 0.1742 | 0.2703 | 0.2493 |
| 438 | 0.2574 | 0.2179 | 0.2198 | 0.2188 | 0.2137 | 0.252 | 0.2421 | 0.2223 | 0.1984 | 0.1848 | 0.2494 | 0.1752 | 0.2646 | 0.2445 |
| 440 | 0.2523 | 0.2178 | 0.2199 | 0.2185 | 0.2131 | 0.2524 | 0.2419 | 0.2228 | 0.1987 | 0.1844 | 0.2468 | 0.1771 | 0.2591 | 0.2393 |
| 442 | 0.2454 | 0.2167 | 0.2192 | 0.2178 | 0.2122 | 0.2508 | 0.2404 | 0.2217 | 0.1979 | 0.1836 | 0.2419 | 0.1788 | 0.2527 | 0.2337 |
| 444 | 0.2395 | 0.215 | 0.2183 | 0.2165 | 0.2096 | 0.2496 | 0.2389 | 0.2209 | 0.1972 | 0.1829 | 0.2371 | 0.1803 | 0.2462 | 0.2281 |
| 446 | 0.2327 | 0.2142 | 0.217 | 0.2153 | 0.2077 | 0.2479 | 0.2366 | 0.2194 | 0.1958 | 0.1822 | 0.2324 | 0.1819 | 0.2399 | 0.2211 |
| 448 | 0.2257 | 0.2121 | 0.2159 | 0.2138 | 0.205 | 0.2461 | 0.2348 | 0.2178 | 0.1941 | 0.1811 | 0.227 | 0.1842 | 0.2316 | 0.2151 |
| 450 | 0.2188 | 0.2098 | 0.2143 | 0.2122 | 0.2026 | 0.2442 | 0.2315 | 0.2165 | 0.1928 | 0.1795 | 0.2208 | 0.1841 | 0.2236 | 0.2086 |
| 452 | 0.2122 | 0.2078 | 0.2121 | 0.21 | 0.1999 | 0.2418 | 0.2286 | 0.2149 | 0.1909 | 0.1779 | 0.2151 | 0.1849 | 0.217 | 0.2012 |
| 454 | 0.203 | 0.2046 | 0.2093 | 0.2067 | 0.1955 | 0.238 | 0.224 | 0.2112 | 0.1884 | 0.1757 | 0.2075 | 0.1854 | 0.2083 | 0.1942 |
| 456 | 0.1959 | 0.2002 | 0.2061 | 0.2039 | 0.1915 | 0.2345 | 0.2206 | 0.2078 | 0.1856 | 0.1732 | 0.2011 | 0.1859 | 0.2004 | 0.1861 |
| 458 | 0.1883 | 0.1964 | 0.2029 | 0.2012 | 0.1873 | 0.2317 | 0.2163 | 0.2047 | 0.182 | 0.1709 | 0.1952 | 0.1854 | 0.1925 | 0.1787 |
| 460 | 0.1828 | 0.1937 | 0.2004 | 0.1979 | 0.183 | 0.2271 | 0.2122 | 0.2004 | 0.1789 | 0.1685 | 0.1886 | 0.1858 | 0.1854 | 0.172 |
| 462 | 0.1764 | 0.1895 | 0.1972 | 0.1953 | 0.1793 | 0.2235 | 0.2078 | 0.1972 | 0.1764 | 0.1656 | 0.1825 | 0.1847 | 0.1793 | 0.1665 |
| 464 | 0.1691 | 0.1853 | 0.1933 | 0.1905 | 0.1732 | 0.2177 | 0.2026 | 0.192 | 0.1723 | 0.1626 | 0.1763 | 0.184 | 0.1708 | 0.1587 |
| 466 | 0.1619 | 0.1797 | 0.1891 | 0.187 | 0.1683 | 0.2132 | 0.1967 | 0.1875 | 0.1683 | 0.1597 | 0.1691 | 0.183 | 0.1637 | 0.1515 |
| 468 | 0.1565 | 0.1748 | 0.185 | 0.1827 | 0.1634 | 0.2095 | 0.192 | 0.1836 | 0.1649 | 0.1564 | 0.1635 | 0.1818 | 0.1574 | 0.1451 |
| 470 | 0.1495 | 0.1697 | 0.181 | 0.1777 | 0.1569 | 0.2029 | 0.1861 | 0.1776 | 0.1599 | 0.1523 | 0.1564 | 0.18 | 0.15 | 0.1382 |
| 472 | 0.1441 | 0.1651 | 0.1769 | 0.1739 | 0.1527 | 0.198 | 0.1802 | 0.1722 | 0.1558 | 0.148 | 0.1505 | 0.1795 | 0.1441 | 0.1323 |
| 474 | 0.1384 | 0.1598 | 0.1724 | 0.1697 | 0.1472 | 0.1929 | 0.1749 | 0.1678 | 0.1519 | 0.1442 | 0.1449 | 0.1777 | 0.137 | 0.1258 |
| 476 | 0.1328 | 0.1552 | 0.1681 | 0.1651 | 0.142 | 0.1888 | 0.17 | 0.1634 | 0.1479 | 0.1409 | 0.1394 | 0.1765 | 0.1326 | 0.1198 |
| 478 | 0.1274 | 0.1505 | 0.165 | 0.1612 | 0.1378 | 0.1841 | 0.1654 | 0.1592 | 0.1451 | 0.137 | 0.1348 | 0.1756 | 0.1267 | 0.1146 |
| 480 | 0.1229 | 0.1465 | 0.1609 | 0.1572 | 0.1334 | 0.179 | 0.1607 | 0.1547 | 0.141 | 0.1324 | 0.1289 | 0.1748 | 0.1212 | 0.1093 |
| 482 | 0.1199 | 0.1436 | 0.1595 | 0.1555 | 0.1306 | 0.1768 | 0.158 | 0.152 | 0.1388 | 0.1298 | 0.126 | 0.175 | 0.118 | 0.1063 |
| 484 | 0.1172 | 0.1419 | 0.1573 | 0.1535 | 0.1288 | 0.1753 | 0.1555 | 0.1504 | 0.1373 | 0.1275 | 0.1228 | 0.1756 | 0.115 | 0.103 |
| 486 | 0.1142 | 0.1398 | 0.1556 | 0.1518 | 0.1269 | 0.1731 | 0.1538 | 0.1482 | 0.136 | 0.125 | 0.1197 | 0.1761 | 0.1121 | 0.1003 |
| 488 | 0.1121 | 0.1387 | 0.1548 | 0.1506 | 0.1255 | 0.1715 | 0.1523 | 0.1467 | 0.1349 | 0.1229 | 0.1168 | 0.1773 | 0.1094 | 0.0971 |
| 490 | 0.1079 | 0.1361 | 0.1539 | 0.1493 | 0.1239 | 0.17 | 0.151 | 0.1458 | 0.1335 | 0.1194 | 0.1129 | 0.1795 | 0.1058 | 0.0939 |
| 492 | 0.1057 | 0.1358 | 0.1538 | 0.1487 | 0.1231 | 0.1702 | 0.1507 | 0.1462 | 0.1331 | 0.1169 | 0.1113 | 0.1819 | 0.1033 | 0.0908 |
| 494 | 0.1032 | 0.1355 | 0.1541 | 0.1495 | 0.1231 | 0.1711 | 0.1507 | 0.1461 | 0.1333 | 0.1149 | 0.1088 | 0.185 | 0.1004 | 0.0883 |
| 496 | 0.1006 | 0.1363 | 0.1561 | 0.1504 | 0.1239 | 0.1719 | 0.1516 | 0.1474 | 0.134 | 0.1134 | 0.1054 | 0.1892 | 0.0981 | 0.0863 |
| 498 | 0.0995 | 0.1382 | 0.1575 | 0.1515 | 0.1249 | 0.1745 | 0.1535 | 0.1503 | 0.1357 | 0.1129 | 0.1043 | 0.193 | 0.0961 | 0.0842 |
| 500 | 0.097 | 0.14 | 0.1613 | 0.1544 | 0.1271 | 0.178 | 0.1563 | 0.1536 | 0.1385 | 0.1121 | 0.1022 | 0.1997 | 0.0943 | 0.082 |
| 502 | 0.0953 | 0.1428 | 0.1645 | 0.1572 | 0.1292 | 0.1819 | 0.1595 | 0.1573 | 0.1405 | 0.1118 | 0.1009 | 0.205 | 0.0927 | 0.0803 |
| 504 | 0.0936 | 0.1459 | 0.1685 | 0.1611 | 0.1325 | 0.1865 | 0.1634 | 0.1617 | 0.1445 | 0.1123 | 0.0993 | 0.2115 | 0.0914 | 0.0789 |
| 506 | 0.0928 | 0.1501 | 0.1733 | 0.1652 | 0.1365 | 0.1921 | 0.1677 | 0.167 | 0.1484 | 0.1132 | 0.0979 | 0.2175 | 0.0898 | 0.0777 |
| 508 | 0.0923 | 0.1548 | 0.1792 | 0.1702 | 0.1405 | 0.1988 | 0.1736 | 0.1729 | 0.1531 | 0.114 | 0.0971 | 0.2251 | 0.0891 | 0.0769 |
| 510 | 0.091 | 0.1601 | 0.1849 | 0.1757 | 0.1453 | 0.2052 | 0.1789 | 0.1787 | 0.1575 | 0.1159 | 0.0964 | 0.2325 | 0.0876 | 0.0758 |
| 512 | 0.0903 | 0.165 | 0.1915 | 0.1807 | 0.1496 | 0.2119 | 0.1851 | 0.1853 | 0.1629 | 0.1186 | 0.0958 | 0.2391 | 0.0871 | 0.0749 |
| 514 | 0.0893 | 0.172 | 0.1992 | 0.1882 | 0.1561 | 0.2207 | 0.1929 | 0.1936 | 0.1696 | 0.1214 | 0.0956 | 0.2472 | 0.0861 | 0.0741 |
| 516 | 0.0887 | 0.1775 | 0.2062 | 0.1939 | 0.1618 | 0.2282 | 0.2003 | 0.2007 | 0.1755 | 0.1251 | 0.0959 | 0.2541 | 0.0859 | 0.0742 |
| 518 | 0.0885 | 0.1847 | 0.2147 | 0.2015 | 0.1683 | 0.2377 | 0.2081 | 0.2098 | 0.1823 | 0.128 | 0.0948 | 0.262 | 0.0857 | 0.0734 |
| 520 | 0.0878 | 0.1915 | 0.2215 | 0.208 | 0.1747 | 0.2459 | 0.2151 | 0.2175 | 0.1883 | 0.1319 | 0.0949 | 0.2673 | 0.0851 | 0.0733 |
| 522 | 0.087 | 0.2002 | 0.2318 | 0.217 | 0.1831 | 0.2567 | 0.2252 | 0.2284 | 0.1964 | 0.137 | 0.0945 | 0.2751 | 0.0848 | 0.0728 |
| 524 | 0.0866 | 0.2079 | 0.24 | 0.2247 | 0.1909 | 0.2665 | 0.2344 | 0.237 | 0.2033 | 0.1419 | 0.0946 | 0.2811 | 0.0842 | 0.0723 |
| 526 | 0.0869 | 0.2158 | 0.2478 | 0.2327 | 0.1987 | 0.2764 | 0.2437 | 0.2468 | 0.211 | 0.1474 | 0.0954 | 0.2862 | 0.0843 | 0.0724 |
| 528 | 0.0867 | 0.2222 | 0.2551 | 0.24 | 0.2059 | 0.2848 | 0.2514 | 0.2545 | 0.2176 | 0.1527 | 0.0958 | 0.2901 | 0.0841 | 0.072 |
| 530 | 0.087 | 0.2297 | 0.2623 | 0.2462 | 0.2127 | 0.2927 | 0.2586 | 0.2625 | 0.224 | 0.1573 | 0.0964 | 0.2936 | 0.0844 | 0.0721 |
| 532 | 0.0863 | 0.2368 | 0.27 | 0.2533 | 0.2201 | 0.3011 | 0.2675 | 0.2708 | 0.2307 | 0.1638 | 0.0962 | 0.2966 | 0.084 | 0.0727 |
| 534 | 0.0871 | 0.2453 | 0.2773 | 0.2609 | 0.2285 | 0.3101 | 0.2769 | 0.28 | 0.238 | 0.1703 | 0.0974 | 0.2987 | 0.0842 | 0.0729 |
| 536 | 0.0867 | 0.2512 | 0.2839 | 0.2675 | 0.2361 | 0.3184 | 0.285 | 0.2872 | 0.2446 | 0.1775 | 0.0977 | 0.2998 | 0.0841 | 0.0725 |
| 538 | 0.0866 | 0.2592 | 0.2907 | 0.2743 | 0.2441 | 0.3266 | 0.2932 | 0.2957 | 0.2515 | 0.185 | 0.0991 | 0.3002 | 0.0845 | 0.0731 |
| 540 | 0.0875 | 0.2657 | 0.2966 | 0.2803 | 0.2517 | 0.3336 | 0.3006 | 0.3032 | 0.2572 | 0.1912 | 0.1001 | 0.2997 | 0.0851 | 0.0737 |
| 542 | 0.0876 | 0.2713 | 0.3016 | 0.2852 | 0.2579 | 0.3392 | 0.3065 | 0.3087 | 0.2618 | 0.1977 | 0.1007 | 0.2985 | 0.0853 | 0.0743 |
| 544 | 0.0878 | 0.2763 | 0.3049 | 0.2892 | 0.2638 | 0.344 | 0.3119 | 0.3134 | 0.2654 | 0.2035 | 0.1019 | 0.2963 | 0.0857 | 0.0749 |
| 546 | 0.0884 | 0.281 | 0.3081 | 0.2936 | 0.2701 | 0.3483 | 0.3178 | 0.3185 | 0.2698 | 0.211 | 0.103 | 0.2928 | 0.0863 | 0.0756 |
| 548 | 0.0887 | 0.2856 | 0.3109 | 0.2967 | 0.2752 | 0.3516 | 0.323 | 0.3218 | 0.2736 | 0.2182 | 0.105 | 0.2892 | 0.0872 | 0.0763 |
| 550 | 0.0896 | 0.2891 | 0.3126 | 0.299 | 0.2799 | 0.354 | 0.3262 | 0.3247 | 0.276 | 0.2243 | 0.1059 | 0.2843 | 0.0874 | 0.0765 |
| 552 | 0.0899 | 0.2922 | 0.3129 | 0.3009 | 0.2841 | 0.3564 | 0.3294 | 0.3273 | 0.2779 | 0.2314 | 0.1082 | 0.279 | 0.0887 | 0.0783 |
| 554 | 0.0907 | 0.2939 | 0.3126 | 0.3012 | 0.2874 | 0.3566 | 0.3316 | 0.3282 | 0.2789 | 0.2366 | 0.1097 | 0.2735 | 0.0889 | 0.079 |
| 556 | 0.0914 | 0.2955 | 0.3113 | 0.3014 | 0.2901 | 0.3563 | 0.3324 | 0.3282 | 0.2792 | 0.2423 | 0.112 | 0.2671 | 0.09 | 0.0802 |
| 558 | 0.0926 | 0.2956 | 0.3092 | 0.2999 | 0.2917 | 0.3539 | 0.3325 | 0.3265 | 0.2785 | 0.2469 | 0.114 | 0.2602 | 0.091 | 0.0818 |
| 560 | 0.0929 | 0.2947 | 0.3061 | 0.2979 | 0.2923 | 0.3514 | 0.3313 | 0.3245 | 0.2776 | 0.2515 | 0.1159 | 0.2531 | 0.0921 | 0.0823 |
| 562 | 0.0946 | 0.2944 | 0.3026 | 0.2952 | 0.2925 | 0.3475 | 0.3296 | 0.3214 | 0.275 | 0.2555 | 0.1187 | 0.2451 | 0.0935 | 0.0847 |
| 564 | 0.0955 | 0.2922 | 0.2986 | 0.2921 | 0.2923 | 0.3433 | 0.3277 | 0.3177 | 0.2724 | 0.2584 | 0.1211 | 0.2381 | 0.0945 | 0.0857 |
| 566 | 0.0969 | 0.2893 | 0.2924 | 0.2877 | 0.2908 | 0.3379 | 0.3235 | 0.3131 | 0.2683 | 0.261 | 0.1241 | 0.2297 | 0.0956 | 0.0869 |
| 568 | 0.0974 | 0.2867 | 0.2875 | 0.2841 | 0.2893 | 0.3322 | 0.3193 | 0.3085 | 0.2651 | 0.2624 | 0.1259 | 0.2225 | 0.0969 | 0.0892 |
| 570 | 0.0989 | 0.2835 | 0.2805 | 0.2787 | 0.2859 | 0.3258 | 0.3142 | 0.3027 | 0.2602 | 0.2638 | 0.1294 | 0.2145 | 0.0982 | 0.0909 |
| 572 | 0.1007 | 0.2799 | 0.274 | 0.273 | 0.282 | 0.3182 | 0.309 | 0.2961 | 0.2559 | 0.2643 | 0.1322 | 0.2081 | 0.0997 | 0.0929 |
| 574 | 0.1022 | 0.2757 | 0.2683 | 0.2675 | 0.2782 | 0.3114 | 0.304 | 0.2896 | 0.2506 | 0.264 | 0.1354 | 0.2011 | 0.1016 | 0.0947 |
| 576 | 0.1033 | 0.2716 | 0.2616 | 0.2618 | 0.2744 | 0.304 | 0.2984 | 0.2823 | 0.2449 | 0.2627 | 0.1385 | 0.1945 | 0.103 | 0.0963 |
| 578 | 0.1044 | 0.2665 | 0.2539 | 0.2557 | 0.2698 | 0.296 | 0.2923 | 0.2763 | 0.2396 | 0.2611 | 0.1424 | 0.1879 | 0.1047 | 0.0989 |
| 580 | 0.1071 | 0.2613 | 0.2461 | 0.2486 | 0.2641 | 0.2864 | 0.2835 | 0.267 | 0.2332 | 0.2587 | 0.1457 | 0.1804 | 0.1064 | 0.1012 |
| 582 | 0.1083 | 0.2555 | 0.2386 | 0.2419 | 0.2584 | 0.2793 | 0.2769 | 0.2601 | 0.2275 | 0.2566 | 0.1494 | 0.1745 | 0.1088 | 0.1037 |
| 584 | 0.1105 | 0.2505 | 0.2308 | 0.2344 | 0.2517 | 0.2704 | 0.2691 | 0.2515 | 0.2202 | 0.2531 | 0.1532 | 0.1677 | 0.1109 | 0.1064 |
| 586 | 0.1129 | 0.2451 | 0.223 | 0.228 | 0.246 | 0.262 | 0.2615 | 0.2438 | 0.214 | 0.2494 | 0.1578 | 0.1618 | 0.1137 | 0.11 |
| 588 | 0.1146 | 0.2385 | 0.216 | 0.2214 | 0.2406 | 0.2536 | 0.2543 | 0.2367 | 0.2072 | 0.2446 | 0.1616 | 0.1558 | 0.1158 | 0.1118 |
| 590 | 0.117 | 0.2332 | 0.2092 | 0.2149 | 0.2342 | 0.2446 | 0.2466 | 0.2287 | 0.2014 | 0.2405 | 0.1654 | 0.1514 | 0.1183 | 0.1152 |
| 592 | 0.1188 | 0.2285 | 0.2019 | 0.2079 | 0.2283 | 0.2374 | 0.2395 | 0.2218 | 0.196 | 0.2362 | 0.1697 | 0.1461 | 0.1207 | 0.1181 |
| 594 | 0.1214 | 0.2225 | 0.1946 | 0.2015 | 0.2211 | 0.2293 | 0.2315 | 0.2136 | 0.1892 | 0.2299 | 0.1747 | 0.1409 | 0.1236 | 0.1213 |
| 596 | 0.1239 | 0.2179 | 0.1883 | 0.1964 | 0.2154 | 0.2217 | 0.2255 | 0.2065 | 0.184 | 0.2251 | 0.1785 | 0.1369 | 0.1257 | 0.1246 |
| 598 | 0.1265 | 0.2128 | 0.1823 | 0.1901 | 0.2099 | 0.2157 | 0.2188 | 0.2002 | 0.1785 | 0.2202 | 0.183 | 0.1324 | 0.1288 | 0.1276 |
| 600 | 0.1294 | 0.208 | 0.1763 | 0.1837 | 0.2028 | 0.2081 | 0.2118 | 0.1934 | 0.1722 | 0.2144 | 0.1882 | 0.1286 | 0.1315 | 0.1318 |
| 602 | 0.1317 | 0.2027 | 0.1695 | 0.1789 | 0.1971 | 0.2013 | 0.2051 | 0.1871 | 0.1669 | 0.2081 | 0.1926 | 0.1239 | 0.1343 | 0.135 |
| 604 | 0.1342 | 0.1978 | 0.1645 | 0.1726 | 0.1909 | 0.1952 | 0.1983 | 0.1809 | 0.1619 | 0.2022 | 0.1975 | 0.1205 | 0.1376 | 0.1388 |
| 606 | 0.1372 | 0.1938 | 0.1603 | 0.1685 | 0.1865 | 0.1898 | 0.1932 | 0.1754 | 0.1579 | 0.1977 | 0.2016 | 0.1181 | 0.1403 | 0.1418 |
| 608 | 0.1389 | 0.1881 | 0.155 | 0.1636 | 0.1811 | 0.1836 | 0.1874 | 0.1702 | 0.1532 | 0.1923 | 0.2059 | 0.1149 | 0.1434 | 0.1454 |
| 610 | 0.1425 | 0.1843 | 0.1498 | 0.1586 | 0.1754 | 0.1774 | 0.1814 | 0.1641 | 0.1488 | 0.1861 | 0.2113 | 0.1115 | 0.1464 | 0.1491 |
| 612 | 0.1449 | 0.1808 | 0.1461 | 0.1547 | 0.1712 | 0.1723 | 0.1769 | 0.1591 | 0.144 | 0.1807 | 0.2155 | 0.109 | 0.1496 | 0.1524 |
| 614 | 0.1482 | 0.1761 | 0.1406 | 0.1499 | 0.166 | 0.1679 | 0.1715 | 0.1549 | 0.14 | 0.1754 | 0.22 | 0.1055 | 0.153 | 0.1562 |
| 616 | 0.1511 | 0.1717 | 0.1367 | 0.1454 | 0.1605 | 0.1622 | 0.1658 | 0.1491 | 0.1358 | 0.1693 | 0.2248 | 0.1024 | 0.1559 | 0.1603 |
| 618 | 0.1537 | 0.1675 | 0.1326 | 0.1415 | 0.1559 | 0.1576 | 0.1613 | 0.1453 | 0.1317 | 0.1644 | 0.2286 | 0.1004 | 0.1592 | 0.1645 |
| 620 | 0.1572 | 0.1645 | 0.1286 | 0.1378 | 0.1508 | 0.153 | 0.1563 | 0.141 | 0.1286 | 0.1598 | 0.233 | 0.0975 | 0.1624 | 0.1679 |
| 622 | 0.1596 | 0.1598 | 0.1249 | 0.1339 | 0.1463 | 0.1477 | 0.1514 | 0.1357 | 0.1249 | 0.1541 | 0.2376 | 0.095 | 0.1658 | 0.1721 |
| 624 | 0.1621 | 0.1571 | 0.1218 | 0.1304 | 0.1427 | 0.1441 | 0.148 | 0.1324 | 0.122 | 0.1501 | 0.2418 | 0.0933 | 0.1692 | 0.1757 |
| 626 | 0.166 | 0.153 | 0.1183 | 0.1276 | 0.1393 | 0.1402 | 0.1432 | 0.1284 | 0.1184 | 0.145 | 0.2449 | 0.0915 | 0.1722 | 0.1793 |
| 628 | 0.1687 | 0.15 | 0.1154 | 0.1242 | 0.1358 | 0.1367 | 0.1404 | 0.1251 | 0.1159 | 0.1411 | 0.2491 | 0.0895 | 0.1749 | 0.183 |
| 630 | 0.1709 | 0.1467 | 0.1128 | 0.1217 | 0.1321 | 0.1342 | 0.1385 | 0.1224 | 0.1132 | 0.1376 | 0.2529 | 0.0876 | 0.1784 | 0.1862 |
| 632 | 0.1741 | 0.1434 | 0.1097 | 0.1194 | 0.1292 | 0.1308 | 0.1334 | 0.119 | 0.1108 | 0.1336 | 0.2555 | 0.0853 | 0.1813 | 0.1901 |
| 634 | 0.1761 | 0.1399 | 0.1065 | 0.1158 | 0.1257 | 0.1276 | 0.1299 | 0.1154 | 0.1074 | 0.1296 | 0.2584 | 0.0833 | 0.1849 | 0.1935 |
| 636 | 0.1786 | 0.1373 | 0.1055 | 0.1133 | 0.1227 | 0.1248 | 0.1268 | 0.1132 | 0.1056 | 0.1266 | 0.2608 | 0.0822 | 0.187 | 0.196 |
| 638 | 0.1814 | 0.1346 | 0.1028 | 0.1116 | 0.1199 | 0.1213 | 0.1248 | 0.1096 | 0.1036 | 0.1234 | 0.2631 | 0.0813 | 0.1903 | 0.1998 |
| 640 | 0.1846 | 0.1319 | 0.0999 | 0.1086 | 0.1164 | 0.1194 | 0.1215 | 0.1074 | 0.1015 | 0.1192 | 0.2658 | 0.0794 | 0.1931 | 0.203 |
| 642 | 0.1868 | 0.128 | 0.0983 | 0.1059 | 0.1133 | 0.1163 | 0.119 | 0.105 | 0.0988 | 0.1155 | 0.2669 | 0.0786 | 0.196 | 0.2063 |
| 644 | 0.1889 | 0.1255 | 0.0961 | 0.1041 | 0.1112 | 0.1136 | 0.1166 | 0.1024 | 0.0976 | 0.1132 | 0.2684 | 0.077 | 0.1991 | 0.208 |
| 646 | 0.1906 | 0.1228 | 0.0954 | 0.1031 | 0.1093 | 0.1124 | 0.114 | 0.1 | 0.0958 | 0.1115 | 0.2701 | 0.0769 | 0.2022 | 0.2113 |
| 648 | 0.1933 | 0.1195 | 0.0926 | 0.1005 | 0.1066 | 0.1099 | 0.1123 | 0.0979 | 0.0937 | 0.1085 | 0.2705 | 0.0755 | 0.2043 | 0.2137 |
| 650 | 0.1956 | 0.1171 | 0.0906 | 0.0988 | 0.104 | 0.1075 | 0.1091 | 0.0952 | 0.092 | 0.1053 | 0.2706 | 0.0733 | 0.2076 | 0.2169 |
| 652 | 0.1967 | 0.1149 | 0.0897 | 0.0968 | 0.1017 | 0.1049 | 0.1067 | 0.094 | 0.0907 | 0.1033 | 0.2705 | 0.0736 | 0.2091 | 0.2188 |
| 654 | 0.1989 | 0.1127 | 0.0877 | 0.0961 | 0.1002 | 0.1037 | 0.1056 | 0.0922 | 0.0886 | 0.1007 | 0.2714 | 0.0723 | 0.2119 | 0.2202 |
| 656 | 0.2006 | 0.1095 | 0.0856 | 0.0944 | 0.0977 | 0.1012 | 0.1036 | 0.0895 | 0.0877 | 0.0996 | 0.271 | 0.0711 | 0.2147 | 0.2229 |
| 658 | 0.2026 | 0.1074 | 0.0843 | 0.0928 | 0.0958 | 0.0995 | 0.101 | 0.0882 | 0.0864 | 0.0971 | 0.2699 | 0.0704 | 0.2167 | 0.2239 |
| 660 | 0.2039 | 0.1057 | 0.0835 | 0.0908 | 0.0941 | 0.099 | 0.0992 | 0.0876 | 0.0855 | 0.0958 | 0.2686 | 0.0705 | 0.2196 | 0.226 |
| 662 | 0.2061 | 0.1037 | 0.0827 | 0.09 | 0.0923 | 0.0981 | 0.0985 | 0.0855 | 0.0839 | 0.0941 | 0.2684 | 0.07 | 0.2212 | 0.2268 |
| 664 | 0.2078 | 0.1021 | 0.0812 | 0.0888 | 0.0909 | 0.0963 | 0.0968 | 0.0851 | 0.0829 | 0.0926 | 0.2671 | 0.0686 | 0.2243 | 0.2289 |
| 666 | 0.2093 | 0.1003 | 0.0802 | 0.087 | 0.0892 | 0.0947 | 0.094 | 0.083 | 0.0817 | 0.0901 | 0.2648 | 0.068 | 0.2255 | 0.2306 |
| 668 | 0.2111 | 0.0974 | 0.0799 | 0.0861 | 0.088 | 0.0931 | 0.0942 | 0.0811 | 0.0799 | 0.0886 | 0.2639 | 0.0679 | 0.2283 | 0.2322 |
| 670 | 0.2126 | 0.0955 | 0.0786 | 0.085 | 0.0866 | 0.0924 | 0.0924 | 0.081 | 0.0787 | 0.0874 | 0.2615 | 0.0677 | 0.2304 | 0.2322 |
| 672 | 0.2145 | 0.0943 | 0.076 | 0.0842 | 0.085 | 0.0904 | 0.0914 | 0.0792 | 0.0781 | 0.0852 | 0.2597 | 0.0661 | 0.2334 | 0.2338 |
| 674 | 0.2163 | 0.0926 | 0.0755 | 0.0834 | 0.0835 | 0.0898 | 0.0891 | 0.0778 | 0.0783 | 0.0845 | 0.2572 | 0.0658 | 0.2357 | 0.2355 |
| 676 | 0.218 | 0.0911 | 0.0751 | 0.0819 | 0.0822 | 0.0883 | 0.0873 | 0.0766 | 0.0766 | 0.0832 | 0.255 | 0.0652 | 0.2383 | 0.2358 |
| 678 | 0.2197 | 0.0891 | 0.0748 | 0.0808 | 0.0804 | 0.0867 | 0.0863 | 0.075 | 0.0754 | 0.0821 | 0.2525 | 0.065 | 0.2415 | 0.2379 |
| 680 | 0.2221 | 0.0878 | 0.0731 | 0.0809 | 0.0796 | 0.0867 | 0.0855 | 0.0755 | 0.0752 | 0.0809 | 0.2514 | 0.0648 | 0.2437 | 0.238 |
| 682 | 0.2238 | 0.0872 | 0.0729 | 0.0794 | 0.0777 | 0.0859 | 0.084 | 0.0738 | 0.074 | 0.0802 | 0.2489 | 0.064 | 0.2461 | 0.239 |
| 684 | 0.2256 | 0.0862 | 0.0723 | 0.0784 | 0.0769 | 0.085 | 0.0836 | 0.0732 | 0.0725 | 0.0787 | 0.2464 | 0.0638 | 0.249 | 0.2407 |
| 686 | 0.2273 | 0.0834 | 0.0715 | 0.0776 | 0.0759 | 0.0842 | 0.0825 | 0.0721 | 0.0715 | 0.0772 | 0.2447 | 0.0632 | 0.2517 | 0.2416 |
| 688 | 0.2303 | 0.0835 | 0.071 | 0.0775 | 0.0754 | 0.083 | 0.0812 | 0.0708 | 0.0722 | 0.077 | 0.2419 | 0.063 | 0.2551 | 0.243 |
| 690 | 0.2326 | 0.0829 | 0.07 | 0.0775 | 0.075 | 0.0828 | 0.0798 | 0.0708 | 0.0708 | 0.0753 | 0.2388 | 0.0631 | 0.2575 | 0.2435 |
| 692 | 0.2337 | 0.0815 | 0.0694 | 0.0764 | 0.0739 | 0.0814 | 0.0798 | 0.0701 | 0.0713 | 0.0751 | 0.237 | 0.0624 | 0.2596 | 0.2452 |
| 694 | 0.236 | 0.0797 | 0.0688 | 0.0747 | 0.0717 | 0.081 | 0.0787 | 0.0694 | 0.0706 | 0.0751 | 0.2362 | 0.0627 | 0.2627 | 0.2459 |
| 696 | 0.237 | 0.0792 | 0.0688 | 0.0746 | 0.0718 | 0.0806 | 0.0774 | 0.0691 | 0.0694 | 0.0728 | 0.2322 | 0.0615 | 0.2655 | 0.2469 |
| 698 | 0.2402 | 0.0788 | 0.0679 | 0.0746 | 0.0713 | 0.0804 | 0.0777 | 0.0684 | 0.0689 | 0.0724 | 0.231 | 0.0627 | 0.2676 | 0.2475 |
| 700 | 0.2408 | 0.0776 | 0.0678 | 0.0744 | 0.0706 | 0.0792 | 0.0763 | 0.0671 | 0.0687 | 0.0718 | 0.2287 | 0.0612 | 0.271 | 0.2492 |
| 702 | 0.2427 | 0.0771 | 0.0676 | 0.0743 | 0.0702 | 0.0787 | 0.0758 | 0.0669 | 0.0685 | 0.0713 | 0.2273 | 0.0616 | 0.2732 | 0.2502 |
| 704 | 0.2443 | 0.0768 | 0.0677 | 0.0739 | 0.0692 | 0.0779 | 0.076 | 0.0667 | 0.0682 | 0.0705 | 0.2249 | 0.0621 | 0.2753 | 0.2503 |
| 706 | 0.2461 | 0.0759 | 0.0671 | 0.0746 | 0.0696 | 0.0784 | 0.0755 | 0.0674 | 0.0682 | 0.0704 | 0.2226 | 0.0621 | 0.2775 | 0.2515 |
| 708 | 0.2471 | 0.0755 | 0.0672 | 0.0743 | 0.0693 | 0.0784 | 0.0753 | 0.0665 | 0.0691 | 0.0708 | 0.2225 | 0.0618 | 0.2802 | 0.2522 |
| 710 | 0.249 | 0.0753 | 0.0675 | 0.0748 | 0.0692 | 0.0789 | 0.0756 | 0.0673 | 0.069 | 0.0703 | 0.2196 | 0.0623 | 0.2816 | 0.2524 |
| 712 | 0.2496 | 0.0751 | 0.0681 | 0.0754 | 0.0693 | 0.0785 | 0.0761 | 0.0674 | 0.0684 | 0.0696 | 0.2187 | 0.0635 | 0.2831 | 0.252 |
| 714 | 0.2514 | 0.0766 | 0.0682 | 0.075 | 0.07 | 0.0779 | 0.0756 | 0.0667 | 0.0688 | 0.0699 | 0.2166 | 0.063 | 0.2838 | 0.2515 |
| 716 | 0.2516 | 0.0759 | 0.0677 | 0.0742 | 0.0694 | 0.0792 | 0.0762 | 0.0667 | 0.0683 | 0.0706 | 0.2158 | 0.0631 | 0.2846 | 0.2517 |
| 718 | 0.25 | 0.074 | 0.067 | 0.0743 | 0.0684 | 0.0764 | 0.0758 | 0.0657 | 0.0674 | 0.0694 | 0.2108 | 0.062 | 0.2838 | 0.2506 |
| 720 | 0.2491 | 0.0736 | 0.0677 | 0.0732 | 0.0662 | 0.0763 | 0.0732 | 0.0654 | 0.0664 | 0.0678 | 0.2082 | 0.0633 | 0.283 | 0.2483 |
| 722 | 0.2475 | 0.072 | 0.0651 | 0.0715 | 0.0664 | 0.0761 | 0.0727 | 0.0644 | 0.0657 | 0.0666 | 0.2041 | 0.061 | 0.2813 | 0.2465 |
| 724 | 0.2463 | 0.0718 | 0.0642 | 0.0713 | 0.0657 | 0.0754 | 0.0716 | 0.0641 | 0.0656 | 0.0664 | 0.1993 | 0.0603 | 0.2788 | 0.2434 |
| 726 | 0.2442 | 0.0707 | 0.0645 | 0.07 | 0.0634 | 0.0732 | 0.0709 | 0.0625 | 0.0652 | 0.0656 | 0.1972 | 0.0596 | 0.2758 | 0.24 |
| 728 | 0.2421 | 0.069 | 0.0629 | 0.0701 | 0.0636 | 0.073 | 0.0701 | 0.0619 | 0.0642 | 0.0659 | 0.195 | 0.0597 | 0.2733 | 0.2365 |
| 730 | 0.2388 | 0.0695 | 0.0632 | 0.0711 | 0.0636 | 0.0731 | 0.0697 | 0.0617 | 0.0646 | 0.0633 | 0.1923 | 0.0591 | 0.2723 | 0.2355 |
| 732 | 0.237 | 0.068 | 0.0638 | 0.0701 | 0.0623 | 0.0732 | 0.0693 | 0.0627 | 0.0639 | 0.0642 | 0.1886 | 0.0599 | 0.2691 | 0.2318 |
| 734 | 0.2336 | 0.0674 | 0.0634 | 0.0697 | 0.0632 | 0.0723 | 0.07 | 0.061 | 0.0645 | 0.0636 | 0.1863 | 0.0603 | 0.2664 | 0.2297 |
| 736 | 0.2305 | 0.0675 | 0.0625 | 0.0699 | 0.0628 | 0.0719 | 0.0687 | 0.0615 | 0.0643 | 0.0636 | 0.1836 | 0.0592 | 0.2622 | 0.2253 |
| 738 | 0.2275 | 0.0671 | 0.0626 | 0.0699 | 0.0627 | 0.0732 | 0.0697 | 0.0621 | 0.0632 | 0.0623 | 0.1808 | 0.0592 | 0.2589 | 0.2214 |
| 740 | 0.2228 | 0.0658 | 0.0622 | 0.0683 | 0.0613 | 0.0722 | 0.0688 | 0.0613 | 0.0631 | 0.0627 | 0.1775 | 0.0589 | 0.2543 | 0.2185 |
| 742 | 0.2196 | 0.0661 | 0.0617 | 0.0694 | 0.0623 | 0.0721 | 0.0679 | 0.0606 | 0.0634 | 0.0631 | 0.1734 | 0.0591 | 0.2499 | 0.2136 |
| 744 | 0.2155 | 0.0664 | 0.0632 | 0.068 | 0.0609 | 0.0719 | 0.068 | 0.0609 | 0.063 | 0.062 | 0.1703 | 0.0613 | 0.2435 | 0.2097 |
| 746 | 0.211 | 0.0652 | 0.062 | 0.0698 | 0.062 | 0.0711 | 0.067 | 0.06 | 0.0631 | 0.0617 | 0.1671 | 0.059 | 0.2396 | 0.2056 |
| 748 | 0.2064 | 0.0652 | 0.0608 | 0.068 | 0.0612 | 0.0709 | 0.0671 | 0.0603 | 0.0634 | 0.0613 | 0.1644 | 0.0593 | 0.2344 | 0.2011 |
| 750 | 0.2018 | 0.0646 | 0.0625 | 0.0683 | 0.0617 | 0.071 | 0.067 | 0.0591 | 0.0625 | 0.0608 | 0.1598 | 0.0592 | 0.2291 | 0.196 |
| 752 | 0.1975 | 0.0653 | 0.0618 | 0.0688 | 0.0616 | 0.0703 | 0.0667 | 0.0598 | 0.0626 | 0.061 | 0.158 | 0.0589 | 0.2241 | 0.1921 |
| 754 | 0.1926 | 0.0645 | 0.0614 | 0.0683 | 0.0614 | 0.0706 | 0.0665 | 0.0602 | 0.0623 | 0.0595 | 0.1549 | 0.059 | 0.2183 | 0.1869 |
| 756 | 0.1893 | 0.0638 | 0.0607 | 0.0671 | 0.0604 | 0.0697 | 0.0655 | 0.0588 | 0.0611 | 0.0591 | 0.1516 | 0.0586 | 0.214 | 0.1843 |
| 758 | 0.1837 | 0.0643 | 0.0601 | 0.0665 | 0.0601 | 0.0688 | 0.067 | 0.0589 | 0.0624 | 0.06 | 0.147 | 0.0588 | 0.2065 | 0.1786 |
| 760 | 0.179 | 0.064 | 0.0604 | 0.0669 | 0.0599 | 0.069 | 0.0658 | 0.0581 | 0.0602 | 0.0583 | 0.1443 | 0.0583 | 0.2021 | 0.1727 |
| 762 | 0.1745 | 0.0638 | 0.0609 | 0.0666 | 0.0585 | 0.0687 | 0.0659 | 0.0573 | 0.0604 | 0.0588 | 0.1411 | 0.0575 | 0.1972 | 0.168 |
| 764 | 0.1705 | 0.0626 | 0.0609 | 0.0673 | 0.0584 | 0.068 | 0.0643 | 0.058 | 0.0608 | 0.0598 | 0.1365 | 0.0584 | 0.1909 | 0.1644 |
| 766 | 0.1647 | 0.0614 | 0.0599 | 0.0676 | 0.0606 | 0.0682 | 0.0651 | 0.0588 | 0.0609 | 0.0583 | 0.1361 | 0.0574 | 0.1869 | 0.1589 |
| 768 | 0.1626 | 0.0622 | 0.0597 | 0.0662 | 0.0581 | 0.0678 | 0.0645 | 0.0589 | 0.0591 | 0.0576 | 0.1332 | 0.0564 | 0.1822 | 0.1552 |
| 770 | 0.1579 | 0.0611 | 0.0594 | 0.0663 | 0.0588 | 0.0679 | 0.0637 | 0.0578 | 0.0601 | 0.0575 | 0.1304 | 0.0573 | 0.176 | 0.1503 |
| 772 | 0.1532 | 0.0624 | 0.06 | 0.0661 | 0.0583 | 0.0684 | 0.0637 | 0.0583 | 0.0588 | 0.0569 | 0.1276 | 0.057 | 0.1725 | 0.1481 |
| 774 | 0.1505 | 0.0612 | 0.0593 | 0.0649 | 0.0577 | 0.0687 | 0.0627 | 0.0578 | 0.059 | 0.057 | 0.1248 | 0.058 | 0.1678 | 0.144 |
| 776 | 0.1469 | 0.0614 | 0.059 | 0.0651 | 0.0576 | 0.0683 | 0.064 | 0.0581 | 0.0587 | 0.0575 | 0.1229 | 0.0571 | 0.1628 | 0.1397 |
| 778 | 0.144 | 0.061 | 0.0589 | 0.066 | 0.0579 | 0.0676 | 0.0635 | 0.0564 | 0.0597 | 0.0576 | 0.1212 | 0.0578 | 0.1589 | 0.1358 |
| 780 | 0.1398 | 0.06 | 0.0594 | 0.0643 | 0.0573 | 0.0677 | 0.0627 | 0.0579 | 0.059 | 0.0557 | 0.1191 | 0.0569 | 0.1547 | 0.1344 |
| 782 | 0.1366 | 0.0603 | 0.0592 | 0.0649 | 0.0582 | 0.0667 | 0.0634 | 0.0566 | 0.0586 | 0.056 | 0.1154 | 0.0574 | 0.1512 | 0.1298 |
| 784 | 0.133 | 0.061 | 0.0585 | 0.0656 | 0.0591 | 0.067 | 0.0628 | 0.0558 | 0.0579 | 0.0558 | 0.1138 | 0.0562 | 0.1492 | 0.128 |
| 786 | 0.1314 | 0.0602 | 0.0585 | 0.0643 | 0.0574 | 0.0672 | 0.0629 | 0.0581 | 0.0583 | 0.0572 | 0.1133 | 0.0571 | 0.1446 | 0.1236 |
| 788 | 0.1276 | 0.0596 | 0.0581 | 0.0657 | 0.0581 | 0.0666 | 0.0619 | 0.0566 | 0.0577 | 0.0563 | 0.1089 | 0.057 | 0.1418 | 0.1209 |
| 790 | 0.1265 | 0.06 | 0.0575 | 0.0642 | 0.0571 | 0.0668 | 0.0622 | 0.0571 | 0.0589 | 0.0552 | 0.1091 | 0.0554 | 0.1395 | 0.1195 |
| 792 | 0.1239 | 0.0604 | 0.0569 | 0.0652 | 0.0577 | 0.0676 | 0.0626 | 0.0579 | 0.0577 | 0.0559 | 0.1082 | 0.0565 | 0.1367 | 0.1171 |
| 794 | 0.1203 | 0.0591 | 0.0586 | 0.0638 | 0.0575 | 0.0672 | 0.0624 | 0.0576 | 0.0574 | 0.0561 | 0.1058 | 0.0567 | 0.1342 | 0.1157 |
| 796 | 0.1194 | 0.0595 | 0.0595 | 0.0643 | 0.0564 | 0.0666 | 0.0628 | 0.0567 | 0.0564 | 0.0558 | 0.1043 | 0.0565 | 0.1324 | 0.1137 |
| 798 | 0.1182 | 0.06 | 0.0587 | 0.0646 | 0.0573 | 0.066 | 0.0626 | 0.0564 | 0.0581 | 0.0567 | 0.1027 | 0.0579 | 0.1289 | 0.1096 |

**Figure S1-11**. UV-Vis of possible 2:1 adducts with various protected azetidines (10.0 mM azetidine, 1.1 mM Rh_2_(esp)_2_). For a more detailed inspection of the data, please see **Supplementary Information 2**.

*Discussion of UV-Vis Adduct Study*

The extent of azetidine binding to the dirhodium catalyst greatly impacts the reaction outcome. The electron-withdrawing (EW) nature of the Boc-, tosyl-, and benzoyl-protecting groups all reduce the basicity of the lone pair of electrons on nitrogen, suppressing the azetidine’s ability to coordinate to the catalyst. The suppressed coordination is evident in **Figure S1-10,11**, where the UV-Vis spectra of these azetidines closely match that of unbound Rh₂(esp)₂, indicating minimal interaction. Notably, a benzoyl-protected azetidine does bind to the catalyst in 10x excess (**Figure S1-11**), yet only in the 1:1 adduct. This result indicates there is still likely a large amount of free catalyst present at equilibrium, which enables the diazoacetate dimerization to persist due to unoccupied axial sites (**Table S1-10,11**). For all three electron-withdrawing protected azetidines, the lack of formation of a 2:1 azetidine:catalyst adduct rationalizes why rapid diazoacetate dimerization is observed, as free catalyst leads to the kinetically preferred dimerization of the diazoacetate. In contrast, the strongly electron-donating ability of the benzyl-protecting group enhances the basicity of the nitrogen lone pair, facilitating a more favorable binding interaction with the catalyst. This is evident in the significant decrease in wavelength of benzyl-azetidine in **Figure S1-10** and **S1-11** compared to the standard. The benzyl-azetidine adduct effectively blocks diazo coordination to the catalyst, suppressing any productive ring expansion or dimerization.

The *N*-aryl protected azetidines strike a balance where the binding of the heterocycle to the catalyst does not shut down the reaction completely but is still able to prevent rapid dimerization of the diazoacetate, ultimately promoting the desired pyrrolidine formation. Interestingly, the electronic environment of the phenyl derivative has seemingly little impact on its ability to bind to rhodium. The most strongly electron-donating derivative, *p*-methoxyphenyl, exhibits the same wavelength shift in the 1:1 adduct as other phenyl-substituted azetidines, with only minor variations observed in the 2:1 complex. The only outlier is *p*-trifluoromethyl phenyl azetidine, bearing a strong electron-withdrawing group; however, its modest decrease in binding affinity does not impact the outcome of the pyrrolidine formation.

To probe the potential steric effects on catalyst binding, azetidines bearing 3,5-dimethylphenyl and 2,6-dimethylphenyl groups on the nitrogen were examined. Both derivatives exhibit significant interaction with the dirhodium catalyst; however, distinct reactivity was observed. The 3,5-dimethylphenyl azetidine undergoes productive ring expansion, whereas the 2,6-dimethylphenyl-protected azetidine furnishes only slow diazoacetate dimerization and fails to form the pyrrolidine (**Figure S1-10,11**). These results suggest that steric hindrance plays a limited role in binding of the azetidine to the catalyst to inhibit diazo dimerization; rather, effective formation of the azetidinium ylide intermediate necessary for ring expansion is disrupted.

# VIII. Equilibrium Constant Determination for Rh_2_(esp)_2_ and Protected Azetidines

Toluene solutions of the protected azetidine (0.2 M) and Rh_2_(esp)_2_ (1.6 mM, 6.08 mg, 0.008 mMol) were prepared the day of the study. Absorption measurements were performed on an Agilent Cary 60 spectrometer in air. Prior to the addition of the azetidine, the UV-Vis (350-1000 nm) spectrum of 3.0 mL of the Rh_2_(esp)_2_ solution was recorded. Then, 5.0 µL of the azetidine solution was added to the cell using a 10.0 µL syringe. The cell was inverted twice to ensure thorough mixing before the UV-vis spectrum was recorded. Nineteen additional aliquots of 5.0 µL and five 10.0 µL aliquots of the ligand solution were sequentially added to the cell over ~20 min. The total change in volume was less than 5%. Upon sequential addition of the azetidine solution, the color of the mixture turned from green to teal, and finally to purple if the azetidine coordinated well to Rh_2_(esp)_2_. The UV-Vis spectrum was recorded after the addition of each aliquot. Measurements were performed in duplicate. Equilibrium constant K_1_ was determined from a plot of 1/Δabsorbance versus 1/[azetidine], which yielded a straight line (R^2^>0.99 with the Y-intercept/slope equal to *k*_1_.^33^ The equilibrium constant *k*_2_ was not determined.

**Figure S1-12**. UV-Vis titration result for Rh_2_(esp)_2_ with **1a**. **A**) Plot of titration curves with increasing concentrations of **1a**. **B**), **C**), and **D**) Association constants calculated at 720, 725, and 715 nm respectively. 720 nm is the wavelength associated with the free acid. For a more detailed inspection of the data, please see **Supplementary Information 2**.

**Figure S1-12-A** is referenced in the main text of the manuscript and exhibits titration curves of Rh_2_(esp)_2_ with increasing concentrations of **1a**. This spectrum shows an isobestic point for a 1:1 complex at 665 nm and low concentrations of **1a**. At high [**1a**], a second isosbestic point at 585 nm was observed for the 2:1 adduct. At intermediate concentrations of **1a**, neither isosbestic point is intersected, suggesting all three adducts coexist in solution.

**Figure S1-13**. UV-Vis titration result for Rh_2_(esp)_2_ with **1-Bn**. **A**) Plot of titration curves with increasing concentrations of **1a**. **B**), **C**), and **D**) Association constants calculated at 725, 720, and 715 nm respectively. 725 nm is the wavelength associated with the free acid. For a more detailed inspection of the data, please see **Supplementary Information 2**.

**Figure S1-14**. UV-Vis titration result for Rh_2_(esp)_2_ with **1-Boc**. **A**) Plot of titration curves with increasing concentrations of **1a**. **B**), **C**), and **D**) Association constants calculated at 720, 710, and 715 nm respectively. 715 nm is the wavelength associated with the free acid. For a more detailed inspection of the data, please see **Supplementary Information 2**.

# IX. Detailed Proposed Mechanism

**Figure S1-15**. A proposed comprehensive mechanism.

A potential mechanism was proposed that accounts for the observed experimental results (**Figure S1-15**). Rh_2_(esp)_2_ (**I**) undergoes a binding equilibrium with *N*-aryl azetidine **1** and/or diethyl ether that favors bound states **II** and **III** due to **1**’s stronger binding affinity to the catalyst than diethyl ether. **IV**, **V**, and **VI** likely contribute more significantly at reduced **1** concentration to continue inhibiting **2** from dimerization. The catalyst pre-equilibrium acts as the *in-situ* “protection” of the catalyst. Slow release of catalyst to the acid state **II** or **IV** permits coordination with carbene precursor **2**, which undergoes N_2_ extrusion to the carbenoid species **VIII**. The low concentration of catalyst states **II** and **IV** prevents dimerization pathways, avoidance of which typically necessitates a slow addition. Nucleophilic attack by azetidine **1** on carbenoid **VIII** and dissociation of catalyst gives ylide intermediate **IX**. From this intermediate, ring expansion occurs to give pyrrolidine **3**. Carbenoid species **VIII** can also participate in an unproductive catalytic cycle, leading to products **X** and **XII**, following coordination of a second diazo species to form its dimer **IX**, and reactivity with the diethyl ether solvent to form the C-H insertion by-product **XI**.

# X. References

(1)	Armarego, W. L. F. Purification of Laboratory Chemicals, 6th ed.; Chai, C. L. L., Series Ed.; Boston: Elsevier/Butterworth-Heinemann, 2009.

(2)	Still, W. C.; Kahn, M.; Mitra, A. Rapid Chromatographic Technique for Preparative Separations with Moderate Resolution. *J. Org. Chem.* **1978**, *43* (14), 2923–2925.

(3)	Chi, W.; Qiao, Q.; Wang, C.; Zheng, J.; Zhou, W.; Xu, N.; Wu, X.; Jiang, X.; Tan, D.; Xu, Z.; Liu, X. Descriptor ΔG_C-O_ Enables the Quantitative Design of Spontaneously Blinking Rhodamines for Live-Cell Super-Resolution Imaging. *Angew. Chem. Int. Ed.* **2020**, *59* (45), 20215–20223.

(4)	Wang, K.; Yang, L.; Li, Y.; Li, H.; Liu, Z.; Ning, L.; Liu, X.; Feng, X. Asymmetric Catalytic Ring-Expansion of 3-Methyleneazetidines with α-Diazo Pyrazoamides towards Proline-Derivatives. *Angew. Chem.* **2023**, *135* (32), e202307249.

(5)	Cai, W.; Wang, S.; Jalani, H. B.; Wu, J.; Lu, H.; Li, G. Oxidative Cascade Reaction of N-Aryl-3-Alkylideneazetidines and Carboxylic Acids: Access to Fused Pyridines. *Org. Lett.* **2018**, *20* (13), 3833–3837.

(6)	Hoque, M. E.; Hassan, M. M. M.; Chattopadhyay, B. Remarkably Efficient Iridium Catalysts for Directed C(sp^2^)–H and C(sp^3^)–H Borylation of Diverse Classes of Substrates. *J. Am. Chem. Soc.* **2021**, *143* (13), 5022–5037.

(7)	Mato, R.; Bournez, C.; Lefebvre, Q. Accessing a Medicinal-Chemistry-Relevant Chemical Space with Sp^2^–Sp^3^ Hybrid Heterocyclic Fragments. *Synthesis* **2024**, *56* (22), 3450–3458.

(8)	Denis, C.; Dubois, M. A. J.; Voisin-Chiret, A. S.; Bureau, R.; Choi, C.; Mousseau, J. J.; Bull, J. A. Synthesis of 3,3-Diarylazetidines by Calcium(II)-Catalyzed Friedel–Crafts Reaction of Azetidinols with Unexpected Cbz Enhanced Reactivity. *Org. Lett.* **2019**, *21* (1), 300–304.

(9)	Miller, D. C.; Lal, R. G.; Marchetti, L. A.; Arnold, F. H. Biocatalytic One-Carbon Ring Expansion of Aziridines to Azetidines via a Highly Enantioselective [1,2]-Stevens Rearrangement. *J. Am. Chem. Soc.* **2022**, *144* (11), 4739–4745.

(10)	Quinodoz, P.; Drouillat, B.; Wright, K.; Marrot, J.; Couty, F. N-Arylazetidines: Preparation through Anionic Ring Closure. *J. Org. Chem.* **2016**, *81* (7), 2899–2910.

(11)	Brito, G. A.; Della-Felice, F.; Luo, G.; Burns, A. S.; Pilli, R. A.; Rychnovsky, S. D.; Krische, M. J. Catalytic Enantioselective Allylations of Acetylenic Aldehydes via 2-Propanol-Mediated Reductive Coupling. *Org. Lett.* **2018**, *20* (13), 4144–4147.

(12)	Kern, N.; Hoffmann, M.; Weibel, J.-M.; Pale, P.; Blanc, A. Short and Efficient Route toward α-Substituted *n*-Arylazetidines from Acetanilides via Mitsunobu Reaction. *Tetrahedron* **2014**, *70* (35), 5519–5531.

(13)	Wang, B. J.; Duncton, M. A. J. A Single-Step Synthesis of Azetidine-3-Amines. *J. Org. Chem.* **2020**, *85* (20), 13317–13323.

(14)	Malojcic, G.; Daniels, M. H.; Williams, B. D.; Yu, M.; Ledeboer, M. W.; Harmange, J.-C. P.; Wang, J. L. Substituted 1,6-Naphthyridine Inhibitors of Cdk5. WO2022011274A1, 2022.

(15)	Hodgson, D. M.; Mortimer, C. L.; McKenna, J. M. Amine Protection/α-Activation with the Tert-Butoxythiocarbonyl Group: Application to Azetidine Lithiation–Electrophilic Substitution. *Org. Lett.* **2015**, *17* (2), 330–333.

(16)	Li, D.; Shen, C.; Si, Z.; Liu, L. Palladium-Catalyzed Fluorinative Bifunctionalization of Aziridines and Azetidines with Gem-Difluorocyclopropanes. *Angew. Chem. Int. Ed.* **2023**, *62* (42), e202310283.

(17)	Lai, G. A Facile and Efficient Synthesis of N-Benzylazetidine. *Synth. Commun.* **2001**, *31* (4), 565–568.

(18)	Pertschi, R.; Weibel, J.-M.; Pale, P.; Blanc, A. Benzosultam Synthesis by Gold(I)-Catalyzed Ammonium Formation/Nucleophilic Substitution. *Org. Lett.* **2019**, *21* (14), 5616–5620.

(19)	Otani, Y.; Nagae, O.; Naruse, Y.; Inagaki, S.; Ohno, M.; Yamaguchi, K.; Yamamoto, G.; Uchiyama, M.; Ohwada, T. An Evaluation of Amide Group Planarity in 7-Azabicyclo[2.2.1]Heptane Amides. Low Amide Bond Rotation Barrier in Solution. *J. Am. Chem. Soc.* **2003**, *125* (49), 15191–15199.

(20)	Keipour, H.; Jalba, A.; Delage-Laurin, L.; Ollevier, T. Copper-Catalyzed Carbenoid Insertion Reactions of α-Diazoesters and α-Diazoketones into Si–H and S–H Bonds. *J. Org. Chem.* **2017**, *82* (6), 3000–3010.

(21)	Nicolle, S. M.; Lewis, W.; Hayes, C. J.; Moody, C. J. Stereoselective Synthesis of Highly Substituted Tetrahydrofurans through Diverted Carbene O-H Insertion Reaction. *Angew. Chem. Int. Ed.***2015**, *54* (29), 8485–8489.

(22)	Peng, Q.; Huang, M.; Xu, G.; Zhu, Y.; Shao, Y.; Tang, S.; Zhang, X.; Sun, J. Asymmetric N-Alkylation of 1H-Indoles via Carbene Insertion Reaction. *Angew. Chem. Int. Ed.***2023**, *62* (47), e202313091.

(23)	De Angelis, L.; Zheng, H.; Perz, M. T.; Arman, H.; Doyle, M. P. Intermolecular [5 + 1]-Cycloaddition between Vinyl Diazo Compounds and Tert-Butyl Nitrite to 1,2,3-Triazine 1-Oxides and Their Further Transformation to Isoxazoles. *Org. Lett.* **2021**, *23* (16), 6542–6546.

(24)	Huang, L.; Wulff, W. D. Catalytic Asymmetric Synthesis of Trisubstituted Aziridines. *J. Am. Chem. Soc.* **2011**, *133* (23), 8892–8895.

(25)	Yang, J.; Ke, C.; Zhang, D.; Liu, X.; Feng, X. Enantioselective Synthesis of 2,2,3-Trisubstituted Indolines via Bimetallic Relay Catalysis of α-Diazoketones with Enones. *Org. Lett.* **2018**, *20* (15), 4536–4539.

(26)	Chen, K.; Zhang, S.-Q.; Brandenberg, O. F.; Hong, X.; Arnold, F. H. Alternate Heme Ligation Steers Activity and Selectivity in Engineered Cytochrome P450-Catalyzed Carbene-Transfer Reactions. *J. Am. Chem. Soc.* **2018**, *140* (48), 16402–16407.

(27)	Zhai, C.; Xing, D.; Jing, C.; Zhou, J.; Wang, C.; Wang, D.; Hu, W. Facile Synthesis of 3-Aryloxindoles via Brønsted Acid Catalyzed Friedel–Crafts Alkylation of Electron-Rich Arenes with 3-Diazooxindoles. *Org. Lett.* **2014**, *16* (11), 2934–2937.

(28)	Denton, J. R.; Sukumaran, D.; Davies, H. M. L. Enantioselective Synthesis of Trifluoromethyl-Substituted Cyclopropanes. *Org. Lett.* **2007**, *9* (14), 2625–2628.

(29)	Fawcett, A.; Murtaza, A.; Gregson, C. H. U.; Aggarwal, V. K. Strain-Release-Driven Homologation of Boronic Esters: Application to the Modular Synthesis of Azetidines. *J. Am. Chem. Soc.* **2019**, *141* (11), 4573–4578.

(30)	Dube, H.; Gommermann, N.; Knochel, P. Synthesis of Chiral α-Aminoalkylpyrimidines Using an Enantioselective Three-Component Reaction. *Synthesis* **2004**, *2004* (12), 2015–2025.

(31)	Braun, M.-G.; Ashkenazi, A.; Beveridge, R. E.; Castanedo, G.; Wallweber, H. A.; Beresini, M. H.; Clark, K. R.; De Bruyn, T.; Fu, L.; Gibbons, P.; Jiang, F.; Kaufman, S.; Kan, D.; Kiefer, J. R.; Leclerc, J.-P.; Lemire, A.; Ly, C.; Segal, E.; Sims, J.; Wang, W.; Wei, W.; Zhao, L.; Schwarz, J. B.; Rudolph, J. Discovery of Potent, Selective, and Orally Available IRE1α Inhibitors Demonstrating Comparable PD Modulation to IRE1 Knockdown in a Multiple Myeloma Model. *J. Med. Chem.* **2024**, *67* (11), 8708–8729.

(32)	Tayama, E.; Nakanome, N. Synthesis of Optically Active 2-Substituted Azetidine-2-Carbonitriles from Chiral 1-Arylethylamine via α-Alkylation of N-Borane Complexes. *RSC Adv.* **2021**, *11* (38), 23825–23837.

(33)	Doyle, M. P.; Winchester, W. R.; Hoorn, J. A. A.; Lynch, V.; Simonsen, S. H.; Ghosh, R. Dirhodium(II) Tetrakis(Carboxamidates) with Chiral Ligands. Structure and Selectivity in Catalytic Metal-Carbene Transformations. *J. Am. Chem. Soc.* **1993**, *115*, 9968–9978.

# XII. NMR

^1^H NMR (500 MHz, CDCl_3_) for **1a 1-phenylazetidine**

^13^C NMR (126 MHz, CDCl_3_) for **1a 1-phenylazetidine**

^1^H NMR (500 MHz, CDCl_3_) for **1b 1-(4-methoxyphenyl)azetidine**

^13^C NMR (126 MHz, CDCl_3_) for **1b 1-(4-methoxyphenyl)azetidine**

^1^H NMR (500 MHz, CDCl_3_) for **1c 1-(p-tolyl)azetidine**

^13^C NMR (126 MHz, CDCl_3_) for **1c 1-(p-tolyl)azetidine**

^1^H NMR (500 MHz, CDCl_3_) for **1d 1-(4-(trifluoromethyl)phenyl)azetidine**

^13^C NMR (126 MHz, CDCl_3_) for **1d 1-(4-(trifluoromethyl)phenyl)azetidine**

^19^F NMR (377 MHz, CDCl_3_) for **1d 1-(4-(trifluoromethyl)phenyl)azetidine**

^1^H NMR (500 MHz, CDCl_3_) for **1e 1-(4-(tert-butyl)phenyl)azetidine**

^13^C NMR (126 MHz, CDCl_3_) for **1e 1-(4-(tert-butyl)phenyl)azetidine**

^1^H NMR (500 MHz, CDCl_3_) for **1f 1-(4-bromophenyl)azetidine**

^13^C NMR (126 MHz, CDCl_3_) for **1f 1-(4-bromophenyl)azetidine**

^1^H NMR (500 MHz, CDCl_3_) for **1g 1-(4-fluorophenyl)azetidine**

^13^C NMR (126 MHz, CDCl_3_) for **1g 1-(4-fluorophenyl)azetidine**

^19^F NMR (377 MHz, CDCl_3_) for **1g 1-(4-fluorophenyl)azetidine**

^1^H NMR (500 MHz, CDCl_3_) for **1h 1-(o-tolyl)azetidine**

^13^C NMR (126 MHz, CDCl_3_) for **1h 1-(o-tolyl)azetidine**

^1^H NMR (500 MHz, CDCl_3_) for **1i 1-(3,5-dimethylphenyl)azetidine**

^13^C NMR (126 MHz, CDCl_3_) for **1i 1-(3,5-dimethylphenyl)azetidine**

^1^H NMR (500 MHz, CDCl_3_) for **1j 2-(azetidin-1-yl)pyridine**

^13^C NMR (126 MHz, CDCl_3_) for **1j 2-(azetidin-1-yl)pyridine**

^1^H NMR (500 MHz, CDCl_3_) for **1k 3,3-difluoro-1-(4-methoxyphenyl)azetidine**

^13^C NMR (126 MHz, CDCl_3_) for **1k 3,3-difluoro-1-(4-methoxyphenyl)azetidine**

^19^F NMR (377 MHz, CDCl_3_) for **1k 3,3-difluoro-1-(4-methoxyphenyl)azetidine**

^1^H NMR (500 MHz, CDCl_3_) for **S1-1l 1-(4-methoxyphenyl)azetidin-3-ol**

^13^C NMR (126 MHz, CDCl_3_) for **S1-1l 1-(4-methoxyphenyl)azetidin-3-ol**

^1^H NMR (500 MHz, CDCl_3_) for **1l 1-(4-methoxyphenyl)azetidin-3-yl methanesulfonate**

^13^C NMR (126 MHz, CDCl_3_) for **1l 1-(4-methoxyphenyl)azetidin-3-yl methanesulfonate**

^1^H NMR (500 MHz, CDCl_3_) for **1m methyl 1-(4-methoxyphenyl)azetidine-3-carboxylate**

^13^C NMR (126 MHz, CDCl_3_) for **1m methyl 1-(4-methoxyphenyl)azetidine-3-carboxylate**

^1^H NMR (500 MHz, CDCl_3_) for **S2-1o 3-hydroxy-N-phenyl-5-(triisopropylsilyl)pent-4-ynamide**

^13^C NMR (126 MHz, CDCl_3_) for **S2-1o 3-hydroxy-N-phenyl-5-(triisopropylsilyl)pent-4-ynamide**

^1^H NMR (500 MHz, CDCl_3_) for **S3-1o 1-phenyl-4-((triisopropylsilyl)ethynyl)azetidin-2-one**

^13^C NMR (126 MHz, CDCl_3_) for **S3-1o 1-phenyl-4-((triisopropylsilyl)ethynyl)azetidin-2-one**

^1^H NMR (500 MHz, CDCl_3_) for **1o 1-phenyl-2-((triisopropylsilyl)ethynyl)azetidine**

^13^C NMR (126 MHz, CDCl_3_) for **1o 1-phenyl-2-((triisopropylsilyl)ethynyl)azetidine**

^1^H NMR (500 MHz, CDCl_3_) for **1p 8-chloro-11-(1-(1-(4-methoxyphenyl)azetidin-3-yl)piperidin-4-ylidene)-6,11-dihydro-5H-benzo[5,6]cyclohepta[1,2-b]pyridine**

^13^C NMR (126 MHz, CDCl_3_) for **1p 8-chloro-11-(1-(1-(4-methoxyphenyl)azetidin-3-yl)piperidin-4-ylidene)-6,11-dihydro-5H-benzo[5,6]cyclohepta[1,2-b]pyridine**

^1^H NMR (500 MHz, CDCl_3_) for **S1-1q isopropyl 2-(4-(4-(3-hydroxyazetidin-1-yl)benzoyl)phenoxy)-2-methylpropanoate**

^13^C NMR (126 MHz, CDCl_3_) for **S1-1q isopropyl 2-(4-(4-(3-hydroxyazetidin-1-yl)benzoyl)phenoxy)-2-methylpropanoate**

^1^H NMR (500 MHz, CDCl_3_) for **1q isopropyl 2-methyl-2-(4-(4-(3-((methylsulfonyl)oxy)azetidin-1-yl)benzoyl)phenoxy)propanoate**

^13^C NMR (126 MHz, CDCl_3_) for **1q isopropyl 2-methyl-2-(4-(4-(3-((methylsulfonyl)oxy)azetidin-1-yl)benzoyl)phenoxy)propanoate**

^1^H NMR (500 MHz, CDCl_3_) for **1r 1-(2,6-dimethylphenyl)azetidine**

^13^C NMR (126 MHz, CDCl_3_) for **1r 1-(2,6-dimethylphenyl)azetidine**

^1^H NMR (500 MHz, CDCl_3_) for **3aa methyl 1,2-diphenylpyrrolidine-2-carboxylate**

^13^C NMR (126 MHz, CDCl_3_) for **3aa methyl 1,2-diphenylpyrrolidine-2-carboxylate**

^1^H NMR (500 MHz, CDCl_3_) for **3ba methyl 1-(4-methoxyphenyl)-2-phenylpyrrolidine-2-carboxylate**

^13^C NMR (126 MHz, CDCl_3_) for **3ba methyl 1-(4-methoxyphenyl)-2-phenylpyrrolidine-2-carboxylate**

^1^H NMR (500 MHz, CDCl_3_) for **3bb methyl 2-(4-fluorophenyl)-1-(4-methoxyphenyl)pyrrolidine-2-carboxylate**

^13^C NMR (126 MHz, CDCl_3_) for **3bb methyl 2-(4-fluorophenyl)-1-(4-methoxyphenyl)pyrrolidine-2-carboxylate**

^19^F NMR (377 MHz, CDCl_3_) for **3bb methyl 2-(4-fluorophenyl)-1-(4-methoxyphenyl)pyrrolidine-2-carboxylate**

^1^H NMR (500 MHz, CDCl_3_) for **3bc methyl 1,2-bis(4-methoxyphenyl)pyrrolidine-2-carboxylate**

^13^C NMR (126 MHz, CDCl_3_) for **3bc methyl 1,2-bis(4-methoxyphenyl)pyrrolidine-2-carboxylate**

^1^H NMR (500 MHz, CDCl_3_) for **3bd methyl 2-(4-bromophenyl)-1-(4-methoxyphenyl)pyrrolidine-2-carboxylate**

^13^C NMR (126 MHz, CDCl_3_) for **3bd methyl 2-(4-bromophenyl)-1-(4-methoxyphenyl)pyrrolidine-2-carboxylate**

^1^H NMR (500 MHz, CDCl_3_) for **3be methyl 1-(4-methoxyphenyl)-2-(p-tolyl)pyrrolidine-2-carboxylate**

^13^C NMR (126 MHz, CDCl_3_) for **3be methyl 1-(4-methoxyphenyl)-2-(p-tolyl)pyrrolidine-2-carboxylate**

^1^H NMR (500 MHz, CDCl_3_) for **3bf methyl 1-(4-methoxyphenyl)-2-(4-(trifluoromethyl)phenyl)pyrrolidine-2-carboxylate**

^13^C NMR (126 MHz, CDCl_3_) for **3bf methyl 1-(4-methoxyphenyl)-2-(4-(trifluoromethyl)phenyl)pyrrolidine-2-carboxylate**

^19^F NMR (377 MHz, CDCl_3_) for **3bf methyl 1-(4-methoxyphenyl)-2-(4-(trifluoromethyl)phenyl)pyrrolidine-2-carboxylate**

^1^H NMR (500 MHz, CDCl_3_) for **3bg methyl 2-(4-(*tert*-butyl)phenyl)-1-(4-methoxyphenyl)pyrrolidine-2-carboxylate**

^13^C NMR (126 MHz, CDCl_3_) for **3bg methyl 2-(4-(*tert*-butyl)phenyl)-1-(4-methoxyphenyl)pyrrolidine-2-carboxylate**

^1^H NMR (500 MHz, CDCl_3_) for **3bh ethyl 1-(4-methoxyphenyl)-2-(thiophen-3-yl)pyrrolidine-2-carboxylate**

^13^C NMR (126 MHz, CDCl_3_) for **3bh ethyl 1-(4-methoxyphenyl)-2-(thiophen-3-yl)pyrrolidine-2-carboxylate**

^1^H NMR (500 MHz, CDCl_3_) for **3bi methyl 1-(4-methoxyphenyl)-2-(1-tosyl-1H-indol-3-yl)pyrrolidine-2-carboxylate**

^13^C NMR (126 MHz, CDCl_3_) for **3bi methyl 1-(4-methoxyphenyl)-2-(1-tosyl-1H-indol-3-yl)pyrrolidine-2-carboxylate**

^1^H NMR (500 MHz, CDCl_3_) for **3bj tert-butyl 4-(2-(ethoxycarbonyl)-1-(4-methoxyphenyl)pyrrolidin-2-yl)-3,6-dihydropyridine-1(2H)-carboxylate**

^13^C NMR (126 MHz, CDCl_3_) for **3bj tert-butyl 4-(2-(ethoxycarbonyl)-1-(4-methoxyphenyl)pyrrolidin-2-yl)-3,6-dihydropyridine-1(2H)-carboxylate**

^1^H NMR (500 MHz, CDCl_3_) for **3bk ethyl 1-(4-methoxyphenyl)-2-methylpyrrolidine-2-carboxylate**

^13^C NMR (126 MHz, CDCl_3_) for **3bk ethyl 1-(4-methoxyphenyl)-2-methylpyrrolidine-2-carboxylate**

^1^H NMR (500 MHz, CDCl_3_) for **3bl 1-(1-(4-methoxyphenyl)-2-phenylpyrrolidin-2-yl)ethan-1-one**

^13^C NMR (126 MHz, CDCl_3_) for **3bl 1-(1-(4-methoxyphenyl)-2-phenylpyrrolidin-2-yl)ethan-1-one**

^1^H NMR (500 MHz, CDCl_3_) for **3bm 1-(4-methoxyphenyl)-2-phenyl-2-(trifluoromethyl)pyrrolidine**

^13^C NMR (126 MHz, CDCl_3_) for **3bm 1-(4-methoxyphenyl)-2-phenyl-2-(trifluoromethyl)pyrrolidine**

^19^F NMR (377 MHz, CDCl_3_) for **3bm 1-(4-methoxyphenyl)-2-phenyl-2-(trifluoromethyl)pyrrolidine**

^1^H NMR (500 MHz, CDCl_3_) for **3bn 1-(4-methoxyphenyl)-7-oxa-1-azaspiro[4.4]nonan-6-one**

^13^C NMR (126 MHz, CDCl_3_) for **3bn 1-(4-methoxyphenyl)-7-oxa-1-azaspiro[4.4]nonan-6-one**

^1^H NMR (500 MHz, CDCl_3_) for **3ko 1-benzyl-4',4'-difluoro-1'-(4-methoxyphenyl)spiro[indoline-3,2'-pyrrolidin]-2-one**

^13^C NMR (126 MHz, CDCl_3_) for **3ko 1-benzyl-4',4'-difluoro-1'-(4-methoxyphenyl)spiro[indoline-3,2'-pyrrolidin]-2-one**

^19^F NMR (377 MHz, CDCl_3_) for **3ko 1-benzyl-4',4'-difluoro-1'-(4-methoxyphenyl)spiro[indoline-3,2'-pyrrolidin]-2-one**

^1^H NMR (500 MHz, CDCl_3_) for **3ca methyl 2-phenyl-1-(p-tolyl)pyrrolidine-2-carboxylate**

^13^C NMR (126 MHz, CDCl_3_) for **3ca methyl 2-phenyl-1-(p-tolyl)pyrrolidine-2-carboxylate**

^1^H NMR (500 MHz, CDCl_3_) for **3da methyl 2-phenyl-1-(4-(trifluoromethyl)phenyl)pyrrolidine-2-carboxylate**

^13^C NMR (126 MHz, CDCl_3_) for **3da methyl 2-phenyl-1-(4-(trifluoromethyl)phenyl)pyrrolidine-2-carboxylate**

^19^F NMR (377 MHz, CDCl_3_) for **3da methyl 2-phenyl-1-(4-(trifluoromethyl)phenyl)pyrrolidine-2-carboxylate**

^1^H NMR (500 MHz, CDCl_3_) for **3ea methyl 1-(4-(*tert*-butyl)phenyl)-2-phenylpyrrolidine-2-carboxylate**

^13^C NMR (126 MHz, CDCl_3_) for **3ea methyl 1-(4-(*tert*-butyl)phenyl)-2-phenylpyrrolidine-2-carboxylate**

^1^H NMR (500 MHz, CDCl_3_) for **3fa methyl 1-(4-bromophenyl)-2-phenylpyrrolidine-2-carboxylate**

^13^C NMR (126 MHz, CDCl_3_) for **3fa methyl 1-(4-bromophenyl)-2-phenylpyrrolidine-2-carboxylate**

^1^H NMR (500 MHz, CDCl_3_) for **3ga methyl 1-(4-fluorophenyl)-2-phenylpyrrolidine-2-carboxylate**

^13^C NMR (126 MHz, CDCl_3_) for **3ga methyl 1-(4-fluorophenyl)-2-phenylpyrrolidine-2-carboxylate**

^19^F NMR (377 MHz, CDCl_3_) for **3ga methyl 1-(4-fluorophenyl)-2-phenylpyrrolidine-2-carboxylate**

^1^H NMR (500 MHz, CDCl_3_) for **3ha methyl 2-phenyl-1-(o-tolyl)pyrrolidine-2-carboxylate**

^13^C NMR (126 MHz, CDCl_3_) for **3ha methyl 2-phenyl-1-(o-tolyl)pyrrolidine-2-carboxylate**

^1^H NMR (500 MHz, CDCl_3_) for **3ia methyl 1-(3,5-dimethylphenyl)-2-phenylpyrrolidine-2-carboxylate**

^13^C NMR (126 MHz, CDCl_3_) for **3ia methyl 1-(3,5-dimethylphenyl)-2-phenylpyrrolidine-2-carboxylate**

^1^H NMR (500 MHz, CDCl_3_) for **3jd methyl 2-(4-bromophenyl)-1-(pyridin-2-yl)pyrrolidine-2-carboxylate**

^13^C NMR (126 MHz, CDCl_3_) for **3jd methyl 2-(4-bromophenyl)-1-(pyridin-2-yl)pyrrolidine-2-carboxylate**

^1^H NMR (500 MHz, CDCl_3_) for **3ka methyl 4,4-difluoro-1-(4-methoxyphenyl)-2-phenylpyrrolidine-2-carboxylate**

^13^C NMR (126 MHz, CDCl_3_) for **3ka methyl 4,4-difluoro-1-(4-methoxyphenyl)-2-phenylpyrrolidine-2-carboxylate**

^19^F NMR (377 MHz, CDCl_3_) for **3ka methyl 4,4-difluoro-1-(4-methoxyphenyl)-2-phenylpyrrolidine-2-carboxylate**

^1^H NMR (500 MHz, CDCl_3_) for **3la methyl 1-(4-methoxyphenyl)-4-((methylsulfonyl)oxy)-2-phenylpyrrolidine-2-carboxylate**

^13^C NMR (126 MHz, CDCl_3_) for **3la methyl 1-(4-methoxyphenyl)-4-((methylsulfonyl)oxy)-2-phenylpyrrolidine-2-carboxylate**

^1^H NMR (500 MHz, CDCl_3_) for **3ma dimethyl 1-(4-methoxyphenyl)-2-phenylpyrrolidine-2,4-dicarboxylate**

^13^C NMR (126 MHz, CDCl_3_) for **3ma dimethyl 1-(4-methoxyphenyl)-2-phenylpyrrolidine-2,4-dicarboxylate**

^1^H NMR (500 MHz, CDCl_3_) for **3na methyl 3-cyano-1-(4-methoxyphenyl)-2-phenylpyrrolidine-2-carboxylate**

^13^C NMR (126 MHz, CDCl_3_) for **3na methyl 3-cyano-1-(4-methoxyphenyl)-2-phenylpyrrolidine-2-carboxylate**

^1^H NMR (500 MHz, CDCl_3_) for **3na’ methyl 3-cyano-1-(4-methoxyphenyl)-2-phenylpyrrolidine-2-carboxylate**

^13^C NMR (126 MHz, CDCl_3_) for **3na’ methyl 3-cyano-1-(4-methoxyphenyl)-2-phenylpyrrolidine-2-carboxylate_2**

^1^H NMR (500 MHz, CDCl_3_) for **3oa methyl 1,2-diphenyl-3-((triisopropylsilyl)ethynyl)pyrrolidine-2-carboxylate**

^13^C NMR (126 MHz, CDCl_3_) for **3oa methyl 1,2-diphenyl-3-((triisopropylsilyl)ethynyl)pyrrolidine-2-carboxylate**

^1^H NMR (500 MHz, CDCl_3_) for **3pa methyl 4-(4-(8-chloro-5,6-dihydro-11H-benzo[5,6]cyclohepta[1,2-b]pyridin-11-ylidene)piperidin-1-yl)-1-(4-methoxyphenyl)-2-phenylpyrrolidine-2-carboxylate**

^13^C NMR (126 MHz, CDCl_3_) for **3pa methyl 4-(4-(8-chloro-5,6-dihydro-11H-benzo[5,6]cyclohepta[1,2-b]pyridin-11-ylidene)piperidin-1-yl)-1-(4-methoxyphenyl)-2-phenylpyrrolidine-2-carboxylate**

^1^H NMR (500 MHz, CDCl_3_) for **3qa methyl 1-(4-(4-((1-isopropoxy-2-methyl-1-oxopropan-2-yl)oxy)benzoyl)phenyl)-4-((methylsulfonyl)oxy)-2-phenylpyrrolidine-2-carboxylate**

^13^C NMR (126 MHz, CDCl_3_) for **3qa methyl 1-(4-(4-((1-isopropoxy-2-methyl-1-oxopropan-2-yl)oxy)benzoyl)phenyl)-4-((methylsulfonyl)oxy)-2-phenylpyrrolidine-2-carboxylate**

^1^H NMR (500 MHz, CDCl_3_) for **4ba methyl 2-phenylpyrrolidine-2-carboxylate**

^13^C NMR (126 MHz, CDCl_3_) for **4ba methyl 2-phenylpyrrolidine-2-carboxylate**

^1^H NMR (500 MHz, CDCl_3_) for **4oa 1 methyl 3-ethynyl-1,2-diphenylpyrrolidine-2-carboxylate**

^13^C NMR (126 MHz, CDCl_3_) for **4oa 1 methyl 3-ethynyl-1,2-diphenylpyrrolidine-2-carboxylate**

^1^H NMR (500 MHz, CDCl_3_) for **4la-2 methyl 4-azido-1-(4-methoxyphenyl)-2-phenylpyrrolidine-2-carboxylate**

^13^C NMR (126 MHz, CDCl_3_) for **4la 2 methyl 4-azido-1-(4-methoxyphenyl)-2-phenylpyrrolidine-2-carboxylate**

^1^H NMR (500 MHz, CDCl_3_) for **4bd (2-(4-bromophenyl)-1-(4-methoxyphenyl)pyrrolidin-2-yl)methanol**

^13^C NMR (126 MHz, CDCl_3_) for **4bd (2-(4-bromophenyl)-1-(4-methoxyphenyl)pyrrolidin-2-yl)methanol**

^1^H NMR (500 MHz, CDCl_3_) for **S2-***ent-***1n methyl (S)-1-(4-methoxyphenyl)azetidine-2-carboxylate**

^13^C NMR (126 MHz, CDCl_3_) for **S2-***ent-***1n methyl (S)-1-(4-methoxyphenyl)azetidine-2-carboxylate**

^1^H NMR (500 MHz, CDCl_3_) for **S3-***ent-***1n 1-(4-methoxyphenyl)azetidine-2-carboxamide**

^13^C NMR (126 MHz, CDCl_3_) for **S3-***ent-***1n 1-(4-methoxyphenyl)azetidine-2-carboxamide**
